# Sjögren’s Syndrome Minor Salivary Gland CD4^+^ Memory T Cells Associate with Glandular Disease Features and Have a Germinal Center T Follicular Helper Transcriptional Profile

**DOI:** 10.3390/jcm9072164

**Published:** 2020-07-08

**Authors:** Michelle L. Joachims, Kerry M. Leehan, Mikhail G. Dozmorov, Constantin Georgescu, Zijian Pan, Christina Lawrence, M. Caleb Marlin, Susan Macwana, Astrid Rasmussen, Lida Radfar, David M. Lewis, Donald U. Stone, Kiely Grundahl, R. Hal Scofield, Christopher J. Lessard, Jonathan D. Wren, Linda F. Thompson, Joel M. Guthridge, Kathy L. Sivils, Jacen S. Moore, A. Darise Farris

**Affiliations:** 1 Oklahoma Medical Research Foundation, Arthritis & Clinical Immunology Program, 825 NE 13th Street, Oklahoma City, OK 73104, USA; michelle-joachims@omrf.org (M.L.J.); drkmleehan@gmail.com (K.M.L.); mikhail.dozmorov@vcuhealth.org (M.G.D.); constantin-georgescu@omrf.org (C.G.); zijian-pan@omrf.org (Z.P.); christina-lawrence@omrf.org (C.L.); caleb-marlin@omrf.org (M.C.M.); susan-macwana@omrf.org (S.M.); astrid-rasmussen@omrf.org (A.R.); kiely-grundahl@omrf.org (K.G.); hal-scofield@omrf.org (R.H.S.); chris-lessard@omrf.org (C.J.L.); jonathan-wren@omrf.org (J.D.W.); linda-thompson@omrf.org (L.F.T.); joel-guthridge@omrf.org (J.M.G.); kathy-sivils@omrf.org (K.L.S.); jsmaiermoore@gmail.com (J.S.M.); 2 College of Dentistry, University of Oklahoma Health Sciences Center, 1201 N Stonewall Avenue, Oklahoma City, OK 73117, USA; lida-radfar@ouhsc.edu (L.R.); david-lewis@ouhsc.edu (D.M.L.); 3 Dean McGee Eye Institute, University of Oklahoma Health Sciences Center, 608 Stanton L. Young Boulevard, Oklahoma City, OK 73104, USA; donstone13@gmail.com; 4 Department of Medicine, University of Oklahoma Health Sciences Center, 1100 N Lindsay Avenue, Oklahoma City, OK 73104, USA; 5 Department of Veteran’s Affairs Medical Center, 931 NE 13th Street, Oklahoma City, OK 73104, USA

**Keywords:** sjögren’s syndrome, salivary gland, T lymphocytes, transcriptome

## Abstract

To assess the types of salivary gland (SG) T cells contributing to Sjögren’s syndrome (SS), we evaluated SG T cell subtypes for association with disease features and compared the SG CD4^+^ memory T cell transcriptomes of subjects with either primary SS (pSS) or non-SS sicca (nSS). SG biopsies were evaluated for proportions and absolute numbers of CD4^+^ and CD8^+^ T cells. SG memory CD4^+^ T cells were evaluated for gene expression by microarray. Differentially-expressed genes were identified, and gene set enrichment and pathways analyses were performed. CD4^+^CD45RA^−^ T cells were increased in pSS compared to nSS subjects (33.2% vs. 22.2%, *p* < 0.0001), while CD8^+^CD45RA^−^ T cells were decreased (38.5% vs. 46.0%, *p* = 0.0014). SG fibrosis positively correlated with numbers of memory T cells. Proportions of SG CD4^+^CD45RA^−^ T cells correlated with focus score (r = 0.43, *p* < 0.0001), corneal damage (r = 0.43, *p* < 0.0001), and serum Ro antibodies (r = 0.40, *p* < 0.0001). Differentially-expressed genes in CD4^+^CD45RA^−^ cells indicated a T follicular helper (Tfh) profile, increased homing and increased cellular interactions. Predicted upstream drivers of the Tfh signature included TCR, TNF, TGF-β1, IL-4, and IL-21. In conclusion, the proportions and numbers of SG memory CD4^+^ T cells associate with key SS features, consistent with a central role in disease pathogenesis.

## 1. Introduction

Sjögren’s syndrome (SS) is a systemic autoimmune disease featuring focal lymphocytic infiltration of salivary and lacrimal glands, antibodies to Ro/SS-A and La/SS-B antigens, and chronic dry eyes and mouth [1]. Though the presence of CD4^+^ T cells in focal salivary gland (SG) lesions is well documented [2,3], their effector role(s) and transcriptional profiles have not been established.

Several lines of evidence implicate CD4^+^ T cells in SS pathology. First, the genetic loci most strongly associated with SS risk are *HLA-DR*, *HLA-DQA1*, and *HLA-DQB1* [4], which encode class II MHC molecules that present antigens to CD4^+^ T cells. Second, CD4^+^ T cells have been shown to predominate in SG lymphocytic foci [5], particularly at earlier time points [6]. Third, SG lesions of 25%–30% of patients contain ectopic, germinal center (GC)-like structures [7,8], the formation and maintenance of which require the activity of CD4^+^ T follicular helper (Tfh) cells [9]. Further, SG plasmablasts produce class-switched, somatically-mutated, clonally-related antibodies in situ [10], underscoring the likelihood of T-helper cell-dependent, ectopic immune reactions in glandular tissue. Finally, single-cell T cell receptor (TCR) analysis demonstrated that SG CD4^+^ T cell clonal expansions are antigen-driven and are associated with reduced salivary flow and increased SG fibrosis [3]. A more recent immunophenotyping study highlighted the presence of both CD4^+^ and CD8^+^ T cells in glandular lesions, elevated HLA-DR expression by glandular CD8^+^ T cells and prominence of plasma cells in the SG [11].

There is no consensus regarding the types of CD4^+^ T cells infiltrating the SG of SS patients. In one study, CD4^+^ T cell clones isolated from cells migrating out of SG tissue in vitro produced interferon (IFN)-γ, IL-2, and IL-10 after stimulation, but not IL-4, consistent with Th1 and possibly T regulatory (Treg) cells, but not Th2 cells [12]. However, cloning of cells can introduce bias, as all glandular T cells may not migrate out of tissue and survive as clones. Another study provided immunohistochemical evidence showing co-expression of CD3 and Bcl-6, suggesting the presence of Tfh cells in SG infiltrates, but only one example was presented [13]. Immunohistological evidence of SG IL-17 expression occurred in SG CD4^+^ T cells in primary SS cases but not in healthy controls or subjects with graft vs. host disease in a study including 10 SS cases and 3 healthy controls [14]. However, the number of subjects exhibiting this result was unclear. Two studies reported increasing Treg infiltration as disease severity increased [15,16]. In contrast, Maehara et al. failed to observe association of Treg gene expression with either the severity of infiltration or with GC-like structures [17].

Unbiased, global gene expression studies are an attractive avenue for exploring the functional state of SG CD4^+^ T cells in SS. Several global gene expression studies have been conducted with whole SG tissue [18,19,20,21,22], but the assignment of differentially-expressed (DE) transcripts to T lymphocytes (much less to CD4^+^ vs. CD8^+^ T cells) is problematic for many genes. Though laser capture microdissection is a powerful approach that can identify SS-associated gene expression patterns in lymphocytic infiltrates [23], assignment of transcripts to CD4^+^ T cells, CD8^+^ T cells, or other lymphocytic lineage cells remains challenging. Further, bulk transcriptome data may primarily reflect cell frequency, making it difficult to assess differences between cases and controls at the cellular level.

In the present study, flow cytometry and microarray analyses of highly purified SG memory CD4^+^ T cells from well-characterized primary SS (pSS) cases and matched sicca controls (nSS) were used to assess disease associations and effector cell phenotypes. Proportions of SG CD4^+^ but not CD8^+^ T cells associated with increasing focus score, corneal damage, and serum antibody levels, while the overall numbers of memory T cells correlated with SG fibrosis. The transcriptomes of SG memory CD4^+^ T cells of SS patients were enriched for genes characteristic of germinal center Tfh cells. Candidate drivers of SS-specific CD4^+^ T-cell gene expression patterns included a dominant role for type II interferon, followed by roles for type I interferons and TNFRSF8. Predicted drivers of the Tfh signature included TCR and CD4 signaling, and the cytokines TNF, TGF-β, IL-4, and IL-21. Taken together, our results strengthen the argument that SG memory CD4^+^ T cells play a prominent role in SS disease pathogenesis.

## 2. Materials and Methods

### 2.1. Participants

All biological samples and clinical and laboratory test values were obtained from the Oklahoma Medical Research Foundation (OMRF) Sjögren’s Research Clinic (OSRC) [24]. Clinic participants were self- or physician-referred, underwent pre-clinic screening regarding oral and ocular symptoms [25], and had at least one ocular and one oral dryness complaint. Participants were classified using the 2002 revised American European Consensus Group (AECG) criteria [26]. Non-SS sicca (nSS) subjects are those who failed to meet AECG criteria for primary SS but had dry eye and/or dry mouth complaints. All participants gave fully informed consent in compliance with the Declaration of Helsinki, and the study was approved by the OMRF Institutional Review Board. Clinical measures and minor SG lip biopsies were taken as described [24]. Percent area of SG fibrosis was determined by morphologic criteria as described [3,27]. Serum autoantibodies were measured using the Bio-Rad BioPlex 2200 ANA system (Bio-Rad, Hercules, CA, USA) [24].

Clinical and demographic data are presented in Table 1, using positive/negative values for clinical tests. Median values for clinical characteristics are presented in Table S1. Unless otherwise noted, continuous variables were used for all other comparisons. The flow cytometry cohort included 51 pSS and 69 nSS subjects. SG weights for calculation of absolute numbers/mg tissue and data for SG area of fibrosis based on morphology [27] were also available from a subset of these subjects (absolute numbers/mg tissue: pSS n = 35, nSS n = 57; SG fibrosis: pSS n = 32, nSS n = 30). The microarray study cohort was a subset of the flow cytometry cohort and included pSS cases with SG focus scores ≥1 (n = 17) and nSS controls without focal lymphocytic sialadenitis or antibodies to Ro or La (n = 15).

### 2.2. Isolation of SG Memory CD4^+^ T Cells and cDNA Preparation

SG biopsy tissue (average of five minor SG/subject) was minced, digested with 750 U/mL Collagenase I (Sigma, St. Louis, MO, USA), 500 U/mL Hyaluronidase IV (Sigma), and 0.1 mg/mL DNAse I (Roche, Basel, Switzerland) in RPMI/10 mM Hepes/5% fetal calf serum (FCS, Gold Coast, Australia) for 1 h at 37 °C, then dissociated with program “B” on a gentleMACS instrument (Miltenyi Biotec, Bergisch Gladbach, Germany) or processed as previously described [28]. Cells were stained with monoclonal antibodies (CD3-PE, CD4-PECy5, CD8-Alexa 488, and CD45RA-V450, Becton Dickinson, Franklin Lakes, NJ, USA) as described [3]. CD3^+^CD4^+^CD45RA^−^ cells excluding propidium iodide were first bulk sorted at high purity using doublet discrimination on a FACSAria (Becton Dickinson). Then 200 cells in ~1 µL were sorted into 6.7 µL SuperAMP buffer (Miltenyi Biotec, Auburn, CA, USA) using a MoFlo-XDP (Beckman-Coulter, Indianapolis, IN, USA) and stored at −80 °C. Samples were shipped on dry ice to Miltenyi Biotec where mRNA isolated with paramagnetic oligo (dT) microbeads underwent proprietary bead-bound cDNA synthesis, 3′-end tailing and labeling, followed by single-primer amplification of cDNA and cDNA purification. Samples showing products of 200–1000 bp were hybridized to Agilent Whole Genome 8 × 60 K Oligo Microarrays, using the SuperAMP service.

### 2.3. Flow Cytometry Analysis

Cytometric data (10^6^ events/subject) from SG single cell suspensions were collected on a FACSAria and analyzed using FlowJo software (FlowJo, LLC, Ashland, OR, USA). SG collected after 7 Dec 2011 were weighed following blotting on sterile gauze to remove excess medium. For these samples, absolute numbers/mg SG tissue of each evaluated T cell subset were calculated.

### 2.4. Microarray Data Analysis

Amplified cDNA samples from 17 pSS and 15 nSS subjects were hybridized to Agilent Whole Human Genome 8 × 60 K microarrays in three batches. All data were pooled to assess potential batch effects by principal components analysis, and gene expression data were quality checked using the *arrayQualityMetrics* R package [29]. Batch effects were equalized via ComBat analysis (sva *R* package v 3.8.0; manual specification of batches). Low-variability genes (defined as <50% of the overall variability distribution) were filtered using the function varFilter in R to reduce the false positive rate. The data were quantile normalized, and differentially-expressed genes (DEG) were detected using the *limma* R package v3.3 [30]. P-values were corrected for multiple testing using the Benjamin–Hochberg procedure. The significance threshold was set at a false discovery rate (FDR) of ≤0.1 resulting in adjusted *p*-values ≤ 0.1. Genes with log fold-change values of at least 1.5 were included in the list of DEG. Data from multiple probes for the same gene were collapsed to one by maximum expression level. DEG by these criteria were subjected to Ingenuity Pathways Analysis (IPA September 2017 Release). Gene set enrichment analysis (GSEA) [31] was used to assess Th1, Th2, Th17, Tfh, Treg, T central memory (Tcm), T effector memory (Tem), and T resident memory (Trm) signatures. The normalized microarray data were submitted to the Gene Expression Omnibus under accession GSE143153.

### 2.5. Salivary Gland Imaging

SG tissues were frozen in OCT compound (Tissue Tek, Sakura Finetek USA, Torrance, CA, USA) in dry ice-cooled 2-methylbutane, then shipped to Zellkraftwerk Gmb H (Hannover, Germany) on dry ice. Cryosections (7 μm) were mounted onto coverslips, attached to Zell-Safe Tissue chips (Zellkrafterk Gmb H, Hannover, Germany) and fixed. Prior to the first stain, autofluorescence was recorded and bleached for 10 s. Antibody staining was performed in consecutive rounds of the following steps: (i) 5 min. incubation with mAb conjugate solution, (ii) wash with Zellkraftwerk wash buffer, (iii) imaging of all positions using ZellScanner One, and (iv) quenching by exposure to HBO^®^ light for 20 s. PE-conjugated mAbs used included CD4 (RPA-T4, Biolgend, San Diego, CA, USA), CD8a (RPA-T8, Biolegend, San Diego, CA), CD20 (LT20, Miltenyi Biotec, Bergisch Gladbach, Germany), and CD21 (Bu32, Biolegend). Nuclei were visualized using Hoescht 33,342 (Invitrogen, Carlsbad, CA, USA).

### 2.6. Other Statistical Analyses

All flow cytometry data comparisons are expressed as mean ± SEM. Data were first tested for normal distributions using the D’Agostino and Pearson normality test in GraphPad Prism 7 (GraphPad Software, La Jolla, CA, USA). Differences between groups were assessed by two-tailed unpaired student’s t- or Mann–Whitney U tests using the continuous variable values unless otherwise noted. Correlations were assessed with Pearson’s (normally distributed) or Spearman’s (non-normally distributed) two-tailed analyses, and contingency analyses were performed by Fisher’s exact test (GraphPad Prism). Multiple regression models adjusted for age were generated using a gamma distribution method (glm via ‘stat’, R [32]), and the outcomes were compared by a two-way ANOVA, with the gamma distribution variable coefficient *p* values reported.

## 3. Results

### 3.1. Memory CD4^+^ T Cells Are Increased in pSS SG

We first characterized SG immune cells in both pSS (n = 51) and nSS (n = 69) subjects using flow cytometry to identify CD3^+^ T cells which were CD4^+^, CD8^+^, double positive, or double negative, as well as proportions of those that were antigen-experienced (CD45RA^−^) memory cells. The gating strategy is shown in Figure S1. Absolute numbers of T cell subsets/mg of SG tissue were calculated for those subjects whose biopsy weights were available (n = 35 pSS, n = 57 nSS).

**Figure 1:**
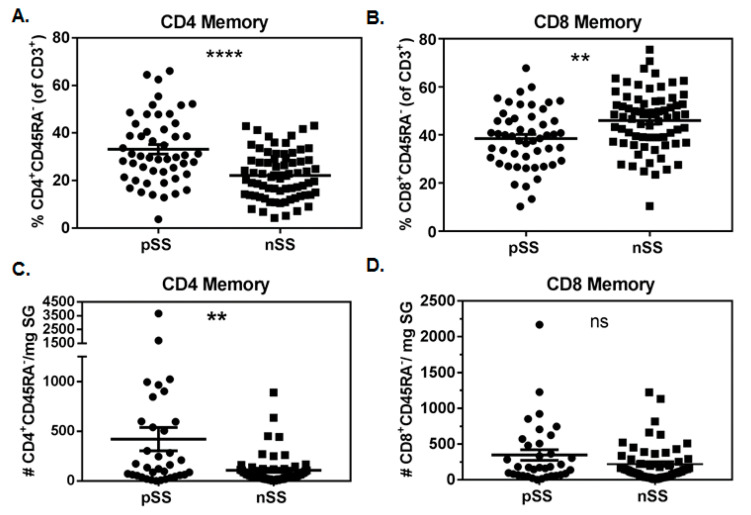
Memory CD4^+^ but not CD8^+^ T cells are increased in salivary glands of Sjögren’s syndrome (SS) cases compared to non-SS controls. Proportions of salivary gland memory CD4 (**A**) and CD8 (**B**) T cells in primary SS cases (pSS, n = 51) and subjects with sicca symptoms not meeting criteria for SS (nSS, n = 69). Absolute numbers of salivary gland memory CD4 (**C**) and CD8 (**D**) T cells/mg of biopsy tissue in pSS (n = 35) and nSS (n = 57). Salivary gland tissue weights were available from only a subset of SS cases and non-SS controls. Data in (**A**,**B**) were normally distributed and evaluated by 2-tailed unpaired student’s *t*-tests. Data in (**C**,**D**) were not normally distributed and were evaluated by 2-tailed Mann–Whitney U tests. Significance is notated: ns = *p* ≥ 0.05, ** = *p* ≤ 0.01, and **** = *p* ≤ 0.0001.

SG of pSS subjects contained a higher proportion of memory CD4^+^ T cells compared to nSS controls (33.2% ± 2.0 vs. 22.2% ± 1.2; *p* < 0.0001, Figure 1A). Conversely, proportions of memory SG CD8^+^ T cells were reduced in pSS cases compared to nSS controls (38.5% ± 1.7 vs. 46.0% ± 1.5; *p* = 0.0014, Figure 1B). No significant differences were observed for proportions of CD3^+^CD4^−^CD8^−^ cells, but pSS subjects had lower proportions of CD3^+^CD4^+^CD8^+^ cells in SG compared to nSS subjects (6.1% ± 1.0 vs. 9.3% ± 1.0; *p* = 0.016, not shown). The vast majority of SG CD4^+^ and CD8^+^ T cells in both pSS (CD4: 93.8% ± 1.0, CD8: 92.1% ± 1.1) and nSS (CD4: 93.8% ± 1.1, CD8: 93.7% ± 1.2) subjects lacked CD45RA expression, and a similar predominance of CD4^+^ T cells was observed in pSS SG when the CD45RA marker was not considered (not shown). The absolute numbers of CD4^+^ memory T cells/mg of SG tissue (Figure 1C) were increased in pSS cases compared to nSS controls (421 ± 117 vs. 108 ± 21; *p* = 0.0025), while numbers of CD8^+^ memory T cells/mg did not differ between the two groups (pSS: 347 cells ± 74, nSS: 220 cells ± 34; *p* = 0.18, Figure 1D). Matched peripheral blood samples were also analyzed in a similar fashion, but no significant differences were observed in any T cell populations comparing pSS and nSS subjects (not shown).

### 3.2. Numbers of SG CD4^+^ Antigen-Experienced T Cells Associate with Biopsy Focus Scores

**Figure 2:**
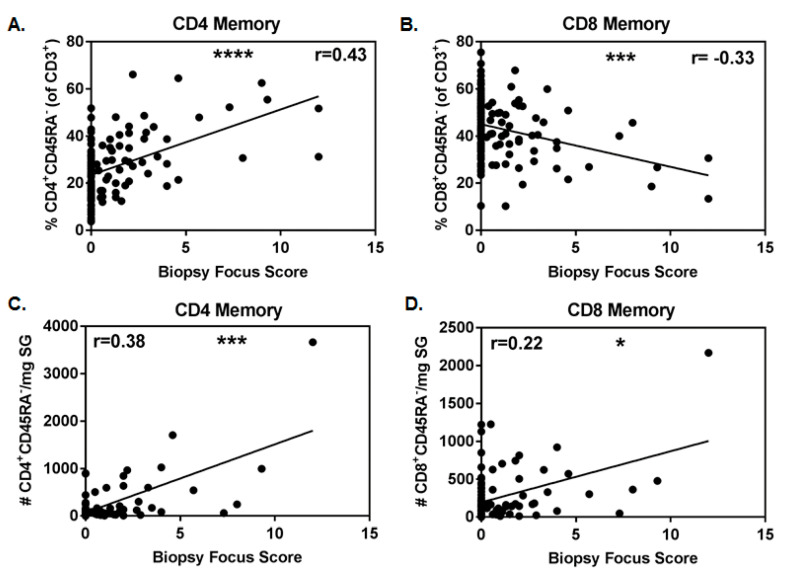
Proportion and number of memory CD4^+^ T cells positively associates with focus score. Correlation of proportions of memory CD4 (**A**) and CD8 (**B**) salivary gland T cells with biopsy focus scores (primary SS (pSS), n = 51, and non-SS sicca (nSS), n = 69). Correlations of absolute numbers of memory CD4 (**C**) and CD8 (**D**) salivary gland T cells/mg biopsy tissue with biopsy focus scores (pSS, n = 35, and nSS, n = 57). Non-normal data in (**A**,**C**,**D**) were evaluated with Spearman’s 2-tailed tests, while normally distributed data in B were evaluated with the Pearson’s 2-tailed test. Significance is notated: ns = *p* ≥ 0.05, * = *p* < 0.05, *** = *p* ≤ 0.001, and **** = *p* ≤ 0.0001.

The diagnosis of SS often requires examination of labial SG biopsy tissue for the presence of focal lymphocytic infiltrates (≥50 lymphocytes/4 mm^2^) adjacent to normal tissue. The number of infiltrates has been linked to disease severity. We found that the proportion of CD4^+^CD45RA^−^ T cells (of CD3^+^ cells) positively associated with increasing focus score (Figure 2A, r = 0.43, *p* < 0.0001), while the inverse was true for the proportion of CD8^+^CD45RA^−^ T cells (Figure 2B, r = −0.33, *p* = 0.0003). In the subset of patients with biopsy tissue weights available, the absolute numbers of CD4^+^CD45RA^−^ T cells positively associated with biopsy focus score (Figure 2C, r = 0.38, *p* = 0.0002). Absolute numbers of CD8^+^CD45RA^−^ T cells also positively associated with biopsy focus score (Figure 2D, r = 0.22, *p* = 0.037), although the association was less robust and was driven by one subject with extremely high cell numbers, as the significance was lost when this data point was removed.

### 3.3. Absolute Numbers of SG Antigen-Experienced T Cells Associate with the Extent of Salivary Gland Fibrosis

As the extent of minor SG fibrosis associates with biopsy focus score and is elevated in pSS patients [27], we examined whether there was a relationship between the composition of the T lymphocyte population in the gland and fibrosis of the tissue. For this analysis, we used existing morphologic fibrosis data from 32 pSS and 30 nSS subjects and cells/mg of tissue data on a subset of these subjects (pSS = 22, nSS = 27). The proportions of neither T cell subset (CD4^+^CD45RA^−^ or CD8^+^CD45RA^−^ T cells) correlated with the degree of minor SG fibrosis (Figure S2). Interestingly, the absolute numbers of both CD4^+^CD45RA^−^ and CD8^+^CD45RA^−^ T cells significantly correlated with the degree of SG fibrosis (r = 0.36 and 0.31, respectively, Figure S2); the precise mechanisms behind this association remain to be defined.

### 3.4. Proportions of SG CD4^+^ Memory T Cells Correlate with Clinical Features of SS

We next asked whether the proportions of SG memory T cells associated with clinical features of SS. Among all subjects in the FACS study, we found that the proportion of memory CD4^+^ cells positively associated with van Bijsterveld (vBS) score (r = 0.43, *p* = 7.2 × 10^−7^), focus score (r = 0.43, *p* = 7.3 × 10^−7^), Ro60 autoantibody positivity (r = 0.4, *p* = 2.6 × 10^−5^), and serum IgG levels (r = 0.38, *p* = 1.7 × 10^−5^) (Table 2-Spearman’s Correlation). As SS patients are usually diagnosed in the fourth decade or later, and loss of salivary flow and SG fibrosis correlate with patient age [27,33], we next asked whether age could explain any associations observed between the proportion of SG CD4^+^CD45RA^−^ T cells and features of SS. We constructed generalized linear models with a gamma distribution to allow the addition of age as a variable to the model. If the addition of age increased the significance of the model, the outcomes were compared by two-way ANOVA tests. With the exception of serum IgG levels, the addition of age did not improve or otherwise alter the model, indicating that the association was not driven by patient age. The association between serum IgG levels and proportion of CD4^+^CD45RA^−^ T cells was improved by adding age into the model (*p* = 0.0014), though there was no direct relationship between age and serum IgG levels. A multivariate gamma regression model showed that both serum IgG levels (*p* = 0.04) and Ro antibody status (*p* = 0.002) independently associated with the proportion of CD4^+^CD45RA^−^ cells (not shown). No significant association was detected between the proportion of memory T cells and Schirmer’s tear flow test results or whole unstimulated salivary flow (WUSF).

Interestingly, when considering only pSS cases (Table S2), the same measures (vBS, focus score, anti-Ro60 titer, and serum IgG) were significantly associated with proportions of CD4^+^ memory T cells in SG. None of the associations in this analysis was impacted by the addition of age to the model. Because higher serum IgG levels are likely a consequence of the autoimmune disease process that generates autoantibodies [34], we compared pSS anti-Ro^+^ and pSS anti-Ro^−^ patients directly and found that anti-Ro^+^ subjects had a significantly higher proportion of SG CD3^+^CD4^+^CD45RA^−^ T cells than anti-Ro^−^ pSS subjects (anti-Ro^+^: 38.3 ± 2.5 vs. anti-Ro^−^: 26.9 ± 2.7; *p* = 0.0027).

### 3.5. Characterization of the pSS CD4^+^CD45RA^−^ SG T Cell Transcriptome

Our analysis of T cell populations demonstrated that CD4^+^CD45RA^−^ T cells are more prevalent in the SG of subjects with pSS and associate with focal lymphocytic infiltrates, corneal damage, serum IgG, and SG fibrosis. In light of these associations, we examined the gene expression profiles of SG CD4^+^CD45RA^−^ T cells from a subset of the pSS cases (n = 17) and nSS sicca controls (n = 15). A total of 506 DEG were identified at a log fold change (FC) threshold of at least 1.5 and FDR of 0.1 (389 upregulated and 117 down-regulated transcripts in pSS compared to nSS subjects). The DEG are listed by significance in Tables S3 and S4.

**Figure 3:**
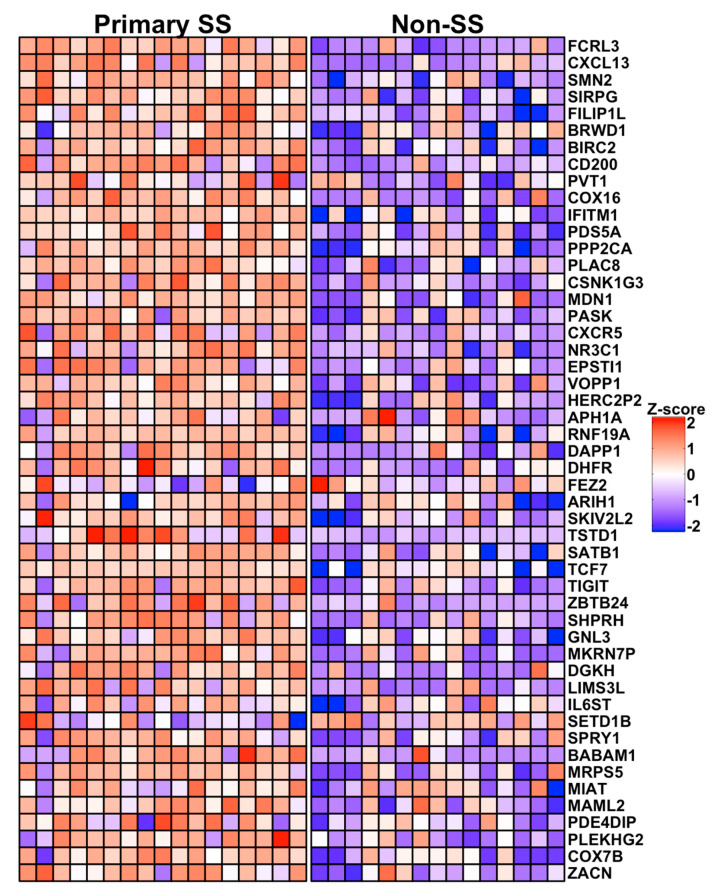
Heat map showing relative expression of the 50 most DEG in salivary gland CD4^+^CD45RA^−^ T cells from pSS cases vs. nSS sicca controls. Columns indicate individual subjects, with pSS sicca cases (Primary SS, n = 17) shown at left and nSS sicca controls (Non-SS, n = 15) shown at right. Rows indicate genes listed in order of significance by adjusted *p*-value. Colors indicate relative expression, with red and blue depicting higher and lower expression, respectively.

A heatmap showing relative expression of the 50 most significant differences (~top 10%) in pSS cases compared to nSS controls is shown in Figure 3. Prominent among this list are transcripts expressed by Tfh cells (CXCL13: logFC = 7.2, Adj *p* = 2.2 × 10^−4^; CD200: logFC = 4.9, Adj *p* = 5.8 × 10^−3^; TCF7: logFC = 4.2, Adj *p* = 2.2 × 10^−2^; CXCR5: logFC = 4.3, Adj *p* = 1.8 × 10^−2^; and TIGIT: logFC = 3.9, Adj *p* = 2.2 × 10^−2^). CXCL13 and CD200 classify germinal center Tfh cells [35,36], while TIGIT is expressed by circulating Tfh with strong B-cell help functions [37]. Other notable transcripts include the IFN-regulated genes IFITM1 (logFC = 4.4, Adj *p* = 1.0 × 10^−2^) and EPSTI1 (logFC = 4.7, Adj *p* = 1.8 × 10^−2^). The top 50 DEG include genes with variants previously associated with SS (CXCR5 [4]), SLE (CXCR5 [38], PPP2CA [39], and TCF7 [40]), and rheumatoid arthritis (FCRL3 [41]).

### 3.6. The Transcriptomes of pSS SG Memory CD4^+^ T Cells Include DEG for Homing, Interaction and Survival Functions

To identify pathways and functions indicative of the transcriptional state of SG CD4^+^ T cells from the pSS cases, expression data from the 506 DEG were subjected to Ingenuity Pathways Analysis. At an IPA significance level of *p* < 0.0001, nine functions had activation Z-scores >2.0, predicting increased pathway activity, including functions related to cellular proliferation, homing, interaction, trafficking, and binding (Figure S3; Table S5). Only two items in the diseases and functions category, lymphoproliferative disorder and cell death, showed predicted decreased activity (activation Z-score <2.0) (Figure S3; Table S5). Significant canonical pathways are listed in Table S6. All pathways with predicted directionality were increased and included signaling through PI3K (−log_10_ *p* = 2.87, z-score 2.33), IL-1 (−log_10_ *p* = 2.49, z-score 2.65), protein kinase A (−log_10_ *p* = 2.31, z = 2.31), RANK (−log_10_ *p* = 1.73, z-score = 2.24), and EIF2 (−log_10_ *p* = 1.49, z-score = 2.24).

Ingenuity Upstream Regulator Analysis predicted 13 protein-encoding upstream regulators of the DEG at *p* < 0.05 and activation Z-scores indicative of positive or negative influence of the predicted regulator (Figure S3; Table S7). These included cytokines (type I interferons, type II interferon), SET phosphatase, hepatocyte growth factor (HGF), MAPK, two transmembrane receptors (TNFRSF8/CD30 and prostaglandin E receptor 4, or PTGER4), and several transcriptional regulators (TRIM24, CREB1, NUPR1, and NKX2-3). Notably, TNFRSF8/CD30 is the receptor for CD30L, encoded by the upregulated DEG TNFSF8.

To understand which enriched IPA functions might be influenced by upstream regulators predicted by IPA, we calculated the percentage of the DEG contributing to each significant IPA function that are known targets of each upstream regulator and displayed the results as a heatmap (Figure S3). This analysis showed that IFNG had the greatest predicted effect by impacting multiple functions, followed by type I interferons, HGF, PTGER4, TNFRSF8, MAPK1, and CREB1. Trafficking and homing of cells was predicted to be particularly influenced by PTGER4, while TNFRSF8/CD30 was predicted to influence lymphocyte interactions, binding, and trafficking.

### 3.7. pSS SG Memory CD4^+^ T Cells Display a Germinal Center Tfh Cell Gene Signature

**Figure 4:**
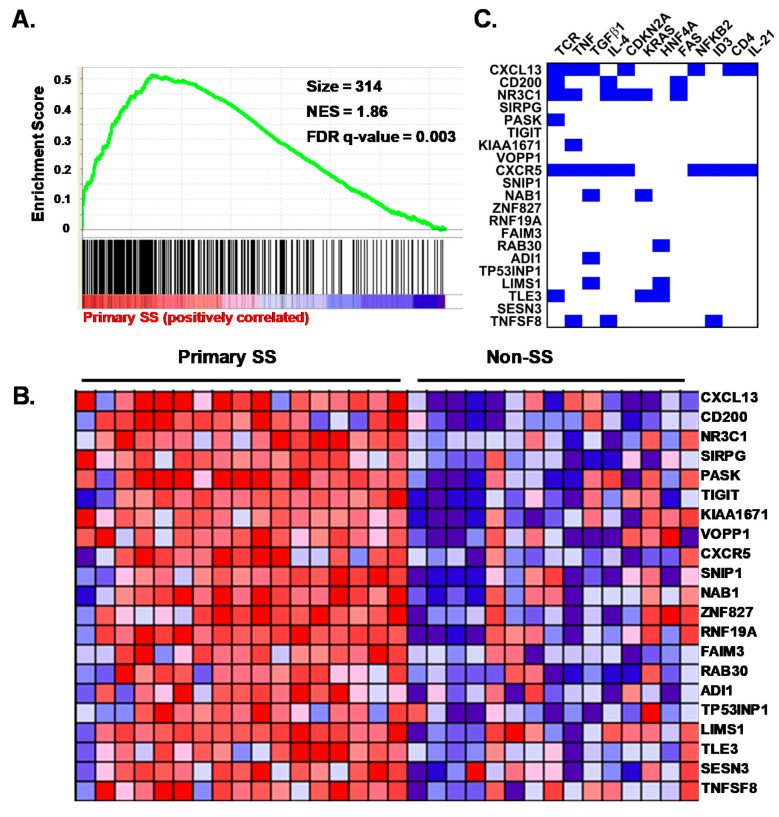
Expressed genes in salivary gland CD4^+^CD45RA^−^ T cells from primary SS cases are enriched for a germinal center T follicular helper (Tfh) cell profile predicted to be regulated by T cell receptor (TCR) and cytokines. (**A**) gene set enrichment analysis (GSEA) plot showing association of the Tfh gene set (Chtanova et al., J Immunol. 173: 68–78, 2004) with pSS. Vertical lines depict the positions of gene set members on the GSEA rank ordered list of all genes differentially expressed between pSS cases and nSS controls. Green line depicts the running enrichment score. (**B**) heat map of the top 21 leading edge genes contributing to enrichment of the Tfh gene set among pSS cases. Columns indicate individual subjects, with pSS cases (Primary SS, n = 17) shown at left and nSS controls (Non-SS, n = 15) shown at right. Rows indicate genes. Colors indicate relative expression, with red and blue depicting higher and lower expression, respectively. (**C**) significant (*p* < 0.05) upstream regulators predicted by ingenuity are listed across the top of the panel, and the most significant leading edge genes from the GSEA analysis shown in Panel A are listed at the left. Blue squares indicate known target genes of the upstream regulators shown based on the Ingenuity knowledge base. Only regulators with the potential to impact more than one of the leading edge genes shown are listed.

To gain further insight into whether any known CD4^+^ T cell differentiation states were enriched among the SG CD4^+^ T cell transcriptomes of the pSS cases compared to the nSS sicca controls, we performed GSEA using published Th1, Th2, Th17, Tfh, Treg, central (Tcm), effector (Tem), and resident memory (Trm) gene sets. In two independent analyses, only the Tfh gene set [35,42] demonstrated significant effects after correction for multiple comparisons (Figure 4A,B; Table S8). Non-significant trends were observed for genes downregulated in Th17 compared to Th0 cells, genes characteristic of Tcm, and genes more highly expressed in activated Treg compared to CD4^+^CD45RA^−^ memory T cells (Table S8). No enrichment in genes characteristic of Th1, Th2, Tem, or Trm cells was detected among the pSS cases (Table S8). Further, no enrichment of any of the tested gene sets was identified among the nSS group. These data indicate a clear Tfh signature among CD4^+^CD45RA^−^ memory T cells from the SG of pSS cases and provide suggestive evidence of enhanced activated Treg and Tcm signatures and a reduced Th17 signature in those with pSS compared to non-SS sicca. To determine whether potential drivers of the Tfh signature could be predicted by integrating the available IPA and GSEA data, we identified overlaps among genes present in the top 21 leading edge genes responsible for Tfh gene set enrichment with DEG that were known targets of predicted upstream drivers identified by IPA in Table S7. As shown in Figure 4C, the predicted regulators affecting the largest number of the top Tfh leading edge genes, ranging from five to six genes each, included TCR signaling, TNF, and TGF-β. Other predicted drivers included IL-4, IL-21, and CD4.

### 3.8. SG biopsies from pSS Subjects Showing SG Memory T Cell Tfh Gene Signatures Display Atypical Ectopic Lymphoid Structures

**Figure 5:**
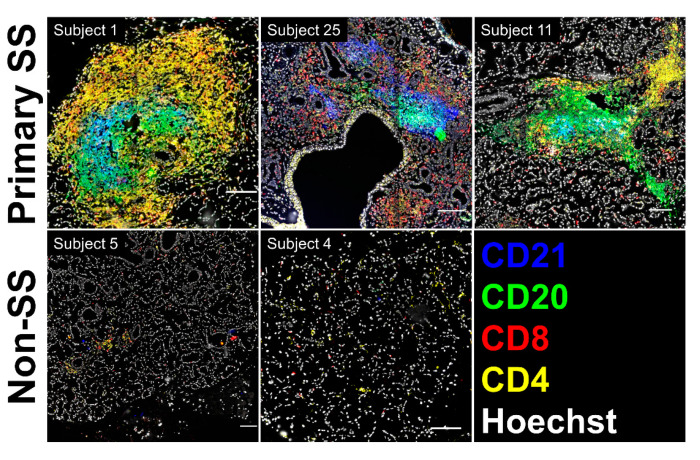
Salivary gland (SG) biopsies from subjects showing SG memory T cell germinal center Tfh transcriptional profiles display atypical ectopic lymphoid structures. Scanned images showing multiplex staining of fixed cryosections for CD4, CD8, CD20, and CD21 in pSS (Primary SS, upper panels) and nSS (Non-SS, lower panels) subjects from the microarray cohort. Scale bars indicate 100 microns.

Although the germinal center Tfh profile was observed in nearly all pSS cases (Figure 3 and Figure 4C), no well-formed germinal center structures were identified in hematoxylin- and eosin-stained salivary gland biopsy cross-sections. To determine whether atypical ectopic germinal center structures could be detected in these individuals, frozen SG sections from three representative pSS and two representative non-SS subjects were evaluated for the presence of lymphocyte aggregates displaying separation of T and B cell areas with coincident CD21^+^ follicular dendritic cell (FDC) networks [43]. As expected, SG sections from the nSS subjects lacking lymphocytic foci showed diffuse and scattered individual CD4^+^ and CD8^+^ cells (Figure 5). In contrast, all three SGs from pSS subjects displayed lymphoid aggregates with detectable underlying networks of CD21^+^ cells (blue staining, Figure 5). In two pSS cases (Subject 1 and Subject 11), these networks were associated with well-segregated T and B cell areas with a dominant presence of CD4^+^ T cells. The CD21^+^ FDC network observed in the SG of the third pSS case (Subject 25) was associated with a mixed CD4^+^/CD8^+^ infiltrate adjacent to a CD20^+^ B cell aggregate.

## 4. Discussion

Here we demonstrate that the proportions and absolute numbers of CD4^+^CD45RA^−^ T cells are increased in the SG of pSS patients compared to nSS sicca controls, are associated with several clinical features of Sjögren’s syndrome, and have a transcriptional profile enriched in genes expressed by germinal center Tfh cells.

Flow cytometry data showed that the primary difference between pSS cases with SG focus score ≥1 and nSS sicca controls is the proportion and number of CD4^+^ memory T cells, consistent with prior immunohistochemical studies showing a prevalence of CD4^+^ T cells in lymphocytic foci [2]. Several additional clinical features correlated with the prevalence of salivary gland memory CD4^+^ T cells, including van Bijsterveld corneal damage score, serum Ro antibody titers, and serum IgG levels. These relationships were observed within the combined pSS/nSS group, as well as within the pSS-only group. In contrast, absolute numbers of both memory CD4^+^ and CD8^+^ T cells correlated with area of salivary gland tissue exhibiting a fibrotic appearance, suggesting that both T cell types could contribute to fibrosis. Neither proportions nor numbers of SG CD4^+^ or CD8^+^ T cells correlated with the European Sjögren’s syndrome Disease Activity Index (ESSDAI), in line with our prior observations showing that the degree of CD4^+^ T cell clonal expansion associated with reduced salivary flow but not ESSDAI [3].

Historically, studies of SG leukocyte populations of SS patients have been limited by the available approaches for evaluating small cell numbers in scant tissue from patient biopsies and have thus focused primarily on whole tissue analyses. To understand the role of individual cell types in disease pathogenesis, separation and characterization of individual subsets is required. Given the association of CD4^+^ memory T cells with disease features we describe here, as well as our prior observation relating salivary gland CD4^+^ T cell clonal expansion with reduced salivary flow and increased SG fibrosis [3], we focused on this population as likely effector cells of glandular disease. We used purified cells and molecular methods to gain new insight into their functional state. These cells showed enrichment of a Tfh gene signature in two independent GSEA analyses. Our observation that SG memory CD4^+^ T cell abundance associated with both serum Ro antibody titers and serum IgG levels lends further support to the identification of these cells as Tfh. As the association between the proportion of SG CD4^+^ T cells and anti-Ro antibodies is not completely explained by serum IgG levels, SG Tfh cells likely help SG B cells produce additional antibody specificities. The detection of *CXCR5*, *CD200*, and *CXCL13* transcripts among the most highly DEG strongly suggests that SG CD4^+^ T cells include a prominent population of germinal center Tfh [36]. Strong expression of *CXCR5* transcripts, in particular, distinguish the profile of these cells from CXCR5^−^ peripheral type Th cells recently found to predominate in the synovial tissue of patients with RA [44].

Although none of the biopsied SGs studied in the microarray cohort displayed well-formed germinal centers, three representative SGs from pSS subjects displayed evidence of atypical ectopic lymphoid structures, defined by the presence of segregated T and B cell areas with underlying CD21^+^ FDC networks [43]. The presence of elevated *CXCL13* transcripts within the SG memory CD4^+^ T cells of these subjects is consistent with an active role for CXCL13 production by the infiltrating lymphoid cells in the organization of ectopic lymphoid structures within the SG of SS patients as has been previously observed [45]. Our findings further contribute to understanding of SG lymphoid infiltrates in SS by revealing specific transcripts responsible for enrichment of the Tfh profile and by identifying potential drivers of this profile.

Suggestive GSEA results that did not pass correction for multiple comparison testing showed trends toward a dearth of Th17 cells and enrichment of Tcm and activated Treg populations in SS SG biopsies. The lack of detection of a Th17 gene signature among salivary gland CD4^+^ T cells is consistent with a prior report finding that CD4^+^ T cells with IL-17 production potential occur at low frequency (<0.2%) among CD4^+^ T cells in SS SG tissue [46]. Our study, which sampled 200 sorted memory CD4^+^ T cells per subject, was designed to detect prevalent differentiation states and to ensure adequate sampling from the non-SS control group. Trends toward enrichment of Tcm and activated Treg phenotypes among SG CD4^+^ T cells will require confirmation in future single cell data sets.

Predominant themes emerging from the pathways analysis included increased cell survival, homing/trafficking and cell-cell interactions, all of which may contribute to the persistence of inflammation. Type I and II interferons, which are dysregulated in SS, were the strongest predicted drivers of these functions. Other potential drivers emerging from our data include TNFRSF8/CD30 and PTGER4. The *TNFSF8* transcript encoding CD30L/CD153 was among the DEG driving enrichment of the Tfh gene signature. CD30L is expressed on activated T cells and germinal center B cells, and has been proposed to play a role in humoral immunity [47]. PTGER4 is expressed on T cells, and its stimulation by prostaglandin E2 is essential for stable T cell-DC interactions and optimal T cell priming in mice [48]. Integration of IPA and GSEA data revealed predicted drivers of the Tfh phenotype in the SS subjects. These included TCR/CD4 co-receptor signaling, TNF, TGF-β, IL-4, and IL-21. Of these, TCR signaling, TNF, TGF-β, and IL-21 have all been shown to promote human Tfh cell differentiation [49,50,51,52]. Although not a known driver of Tfh per se, IL-4 has been shown to cause robust expression of CD40L by T cells and promotion of antibody-secreting B cells [53].

We conclude that SG CD4^+^ T cells are a major cell type in SG focal lymphocytic infiltrates, associating with increased corneal damage and serum antibody levels. This is the first study to interrogate the transcriptomes of purified SG CD4^+^ T cells of pSS cases and nSS sicca controls, revealing differences at the cellular level and enrichment for a germinal center Tfh profile.

## Figures and Tables

**Table 1:** Patient clinical and demographic information.

	FACS Study			Microarray Study		
Demographics	pSS (n = 51)	nSS (n = 69)	*p*-Value	pSS (n = 17)	nSS (n = 15)	*p*-Value
Age (years)	51 ± 1.9	49 ± 1.4	0.24 ^a^	51.8 ± 3.2	42.1 ± 2.2	0.022 ^a^
Gender (% Fem)	50/51 (98)	64/69 (92.8)	0.24 ^b^	16/17 (94)	13/15 (87)	0.58 ^b^
Race (%)						
White	30/51 (59)	35/69 (51)	0.58 ^d^	11/17 (65)	4/15 (24)	0.09 ^d^
NatAm/> one ^c^	19/51 (37)	29/69 (42)		5/17 (29)	10/15 (66)	
Black/Asian	2/51 (4)	5/69 (7)		1/17 (6)	1/15 (7)	
Ethnicity						
Hispanic	2/51 (4)	4/69 (6)	1.0 ^b^	6/17 (35)	7/15 (47)	1.0 ^b^
Clinical Features	pSS	nSS	*p*-value ^b^	pSS	nSS	*p*-value ^b^
FS+ (%) ^e^	32/51 (64)	4/69 (6)	<0.0001	17/17 (100)	0/15 (0)	<0.0001
Anti-Ro+ (%) ^f^	28/51 (55)	4/69 (6)	<0.0001	13/17 (76)	0/15 (0)	<0.0001
Anti-La+ (% +) ^f^	20/51 (39)	1/69 (1)	<0.0001	4/17 (24)	0/15 (0)	0.104
WUSF+ (% +) ^g^	30/51 (59)	32/69 (46)	0.19	11/17 (65)	3/15 (20)	0.016
Schirmer’s+ (%) ^h^	19/51 (37)	18/69 (26)	0.23	6/17 (35)	4/15 (27)	0.712
vBS+ (%) ^i^	35/51 (69)	26/69 (38)	0.0009	12/17 (71)	3/15 (20)	0.006

^a^ unpaired *t*-test; ^b^ Fisher’s exact test; ^c^ Native American and more than one race; ^d^ chi-square test; ^e^ focus score ≥1; ^f^ bioplex value >1.0; ^g^ whole unstimulated salivary flow <1.5 mL/15 min; ^h^ Schirmer’s test <5 mm/5 min; and ^i^ van Bijsterveld score, maximum value ≥4.

**Table 2:** Proportion of CD4^+^CD45RA^−^ cells in CD3^+^ T cells associates with clinical features of disease ^a^.

Clinical Feature	Spearman’s Correlation ^a^		Gamma Regression Model	
	r	*p*-Value	Model	Variable *p*-Value
vBS ^b^	0.43	**7.2 × 10^−7^**	x + age	**6.7 × 10^−5^**
			x	**1.5 × 10^−5^**
Schirmer’s test ^c^	−0.087	0.34	x + age	0.73
			x	0.39
WUSF ^d^	−0.09	0.31	x + age	0.77
			x	0.67
Biopsy Focus Score	0.43	**7.3 × 10^−7^**	x + age	**9.0 × 10^−7^**
			x	**2.3 × 10^−8^**
Anti-Ro Ab ^e^	0.40	**2.6 × 10^−5^**	x + age	**1.1 × 10^−5^**
			x	**3.0 × 10^−6^**
Serum IgG	0.38	**1.7 × 10^−5^**	x + age ^f^	**9.0 × 10^−7^**
			x	**1.4 × 10^−5^**

^a^ Spearman’s 2-tailed test, pSS (n = 51) and nSS (n = 69), values with significance of *p* < 0.05 are shown in bold; ^b^ van Bijsterveld score, maximum value; ^c^ Shirmer’s test, minimal score; ^d^ whole unstimulated salivary flow (mL/15 min); ^e^ bioplex test score for Spearman correlation; bioplex positive (>1.0) status for gamma regression model; ^f^ serum IgG, x + age, significance of ANOVA *p* = 0.0014.

## Supplementary Materials

The following are available online at https://www.mdpi.com/2077-0383/9/7/2164/s1, Figure S1: Gating strategy for CD3^+^CD4^+^CD45RA^−^ and CD3^+^CD8^+^CD45RA^−^ T cells, Figure S2: Salivary gland fibrosis is correlated with numbers, but not proportions of SG tissue resident T cells, Figure S3: Functions and upstream drivers predicted by Ingenuity Pathways Analysis (IPA), Table S1: Clinical Exam Values, Table S2: SG Memory T cell proportion associations with clinical features in pSS (n = 51), Table S3: Genes up-regulated in pSS salivary gland CD3^+^CD4^+^CD45RA^−^ T cells, Table S4: Genes down-regulated in pSS salivary gland CD3^+^CD4^+^CD45RA^−^ T cells, Table S5: Ingenuity Diseases and Functions enriched among DEGs in SG CD4^+^CD45RA^−^ T cells of pSS cases vs. nSS controls, Table S6: Ingenuity Canonical Pathways enriched among DEGs in SG CD4^+^CD45RA^−^ T cells of pSS cases vs. nSS controls, Table S7: Ingenuity Predicted Upstream Regulators of DEGs in SG CD4^+^CD45RA^−^ T cells of pSS cases vs. nSS controls, Table S8: Gene Set Enrichment Analyses.
