# Supplementary material for: Sjögren’s Syndrome Minor Salivary Gland CD4+ Memory T Cells Associate with Glandular Disease Features and Have a Germinal Center T Follicular Helper Transcriptional Profile
Source: J Clin Med. 2020 Jul 8;9(7):2164. doi: 10.3390/jcm9072164 (PMC7408878; doi:10.3390/jcm9072164)
Supplement: Supplementary file 1 [file jcm-09-02164-s001.pdf]

Supplementary Figures & Tables

Sjögren's syndrome minor salivary gland CD4⁺ memory T cells associate with glandular disease features and have a germinal center T follicular helper transcriptional profile

Michelle L. Joachims [1], Kerry M. Leehan [1], Mikhail G. Dozmorov [1,◊], Constantin Georgescu [1,∞], Zijian Pan [1], Christina Lawrence [1], M. Caleb Marlin [1], Susan Macwana [1], Astrid Rasmussen [1,∞], Lida Radfar [2], David M. Lewis [2], Donald U. Stone [3,†], Kiely Grundahl [1,∞], R. Hal Scofield [1,4,5], Christopher J. Lessard [1,∞], Jonathan D. Wren [1,∞], Linda F. Thompson [1], Joel M. Guthridge [1], Kathy L. Sivils [1], Jacen S. Moore [1,‡], and A. Darise Farris [1,*]

[1] Oklahoma Medical Research Foundation, Arthritis & Clinical Immunology Program, 825 NE 13th Street, Oklahoma City, Oklahoma, USA 73104; michelle-joachims@omrf.org (M.L.J.); drkmleehan@gmail.com (K.M.L.); mikhail.dozmorov@vcuhealth.org (M.G.D.); constantin-georgescu@omrf.org (C.G.); zijian-pan@omrf.org (Z.P.); christina-lawrence@omrf.org (C.L.); caleb-marlin@omrf.org (M.C.M.); susan-macwana@omrf.org (S.M.); astrid-rasmussen@omrf.org (A.R.); kiely-grundahl@omrf.org (K.G.); hal-scofield@omrf.org (R.H.S.); chris-lessard@omrf.org (C.J.L.); jonathan-wren@omrf.org (J.D.W.); linda-thompson@omrf.org (L.F.T.); joel-guthridge@omrf.org (J.M.G.); kathy-sivils@omrf.org (K.L.S.); jsmaiermoore@gmail.com (J.S.M.); darise-farris@omrf.org (A.D.F.)

[2] University of Oklahoma Health Sciences Center, College of Dentistry, 1201 N Stonewall Avenue, Oklahoma City, Oklahoma, USA 73117; lida-radfar@ouhsc.edu (L.R.); david-lewis@ouhsc.edu (D.M.L.)

[3] University of Oklahoma Health Sciences Center, Dean McGee Eye Institute, 608 Stanton L. Young Boulevard, Oklahoma City, Oklahoma, USA 73104; donstone13@gmail.com (D.U.S.)

[4] University of Oklahoma Health Sciences Center, Department of Medicine, 1100 N Lindsay Avenue, Oklahoma City, Oklahoma, USA 73104; hal-scofield@omrf.org (R.H.S.)

[5] Department of Veteran's Affairs Medical Center, 931 NE 13th Street, Oklahoma City, Oklahoma, USA 73104; hal-scofield@omrf.org (R.H.S.)

[◊] Current Affiliation: Virginia Commonwealth University, Department of Biostatistics, Department of Pathology, 830 E Main Street, Richmond, Virginia, USA 23298; mikhail.dozmorov@vcuhealth.org (M.G.D.)

[∞] Current Affiliation: Oklahoma Medical Research Foundation, Genes and Human Disease Research Program, 825 NE 13th Street, Oklahoma City, Oklahoma, USA 73104; constantin-georgescu@omrf.org (C.G.); astrid-rasmussen@omrf.org (A.R.); kiely-grundahl@omrf.org (K.G.); chris-lessard@omrf.org (C.J.L.); jonathan-wren@omrf.org (J.D.W.)

[†] Current Affiliation: Spokane Eye Clinic, 427 S Bernard Street, Spokane, Washington, USA 99204; donstone13@gmail.com (D.U.S.)

[‡] Current Affiliation: University of Tennessee Health Science Center, Clinical Laboratory Science Program, Department of Diagnostic and Health Sciences, 910 Madison Avenue, Memphis, Tennessee, USA 38163; jsmaiermoore@gmail.com (J.S.M.)

[*]**Corresponding Author:** A. Darise Farris, PhD, Arthritis and Clinical Immunology Research Program, Oklahoma Medical Research Foundation; Address: 825 NE 13th Street, MS 24, Oklahoma City, OK, USA 73104; Phone: 405-271-7389; Fax: 405-271-4110; Email: darise-farris@omrf.org

Running Title: Sjögren's syndrome minor salivary gland CD4⁺ T cells

Financial Support: This research was funded by NATIONAL INSTITUTES OF HEALTH (NIH) Oklahoma Sjögren's Syndrome Center of Research Translation grant number AR060804 (A.D.F., K.L.S., R.H.S.), NIH grant number AR074310 (A.D.F.), NIH grant number GM110766 (J.S.M.), by an Innovative Research

Award from the RHEUMATOLOGY RESEARCH FOUNDATION (A.D.F.), by an unrestricted grant from RESEARCH TO PREVENT BLINDNESS (University of Oklahoma Health Sciences Center Department of Ophthalmology), and by the NIH Oklahoma Shared Clinical and Translational Resource grant number GM104938 (J.M.G.), NIH Oklahoma Rheumatic Disease Research Cores Center grant number AR073750 (J.M.G.), NIH Autoimmunity Centers of Excellence grant number AI144292 (J.M.G.), and the PRESBYTERIAN HEALTH FOUNDATION (J.M.G.).

COI Disclosures: None

Supplementary Figures: 3

Supplementary Tables: 8

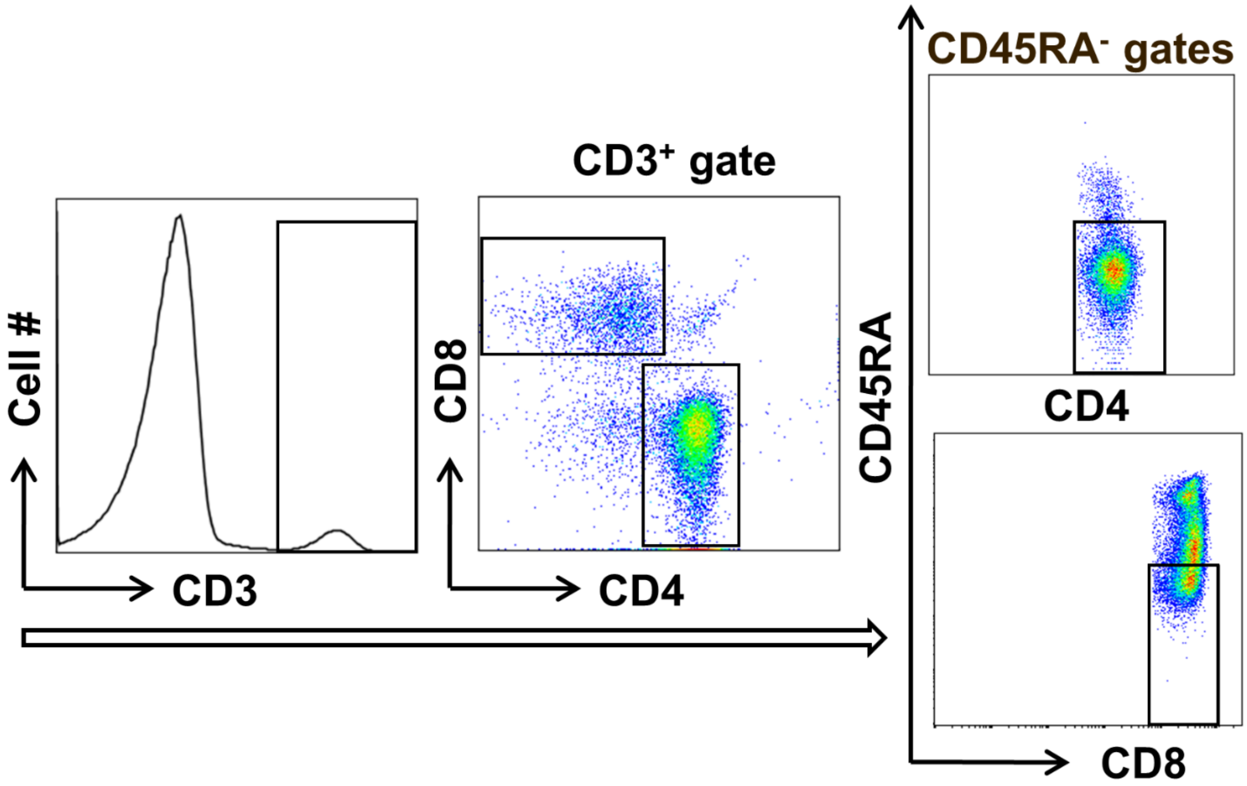

Figure S1. Gating strategy for CD3$^+$CD4$^+$CD45RA$^-$ and CD3$^+$CD8$^+$CD45RA$^-$ T cells.

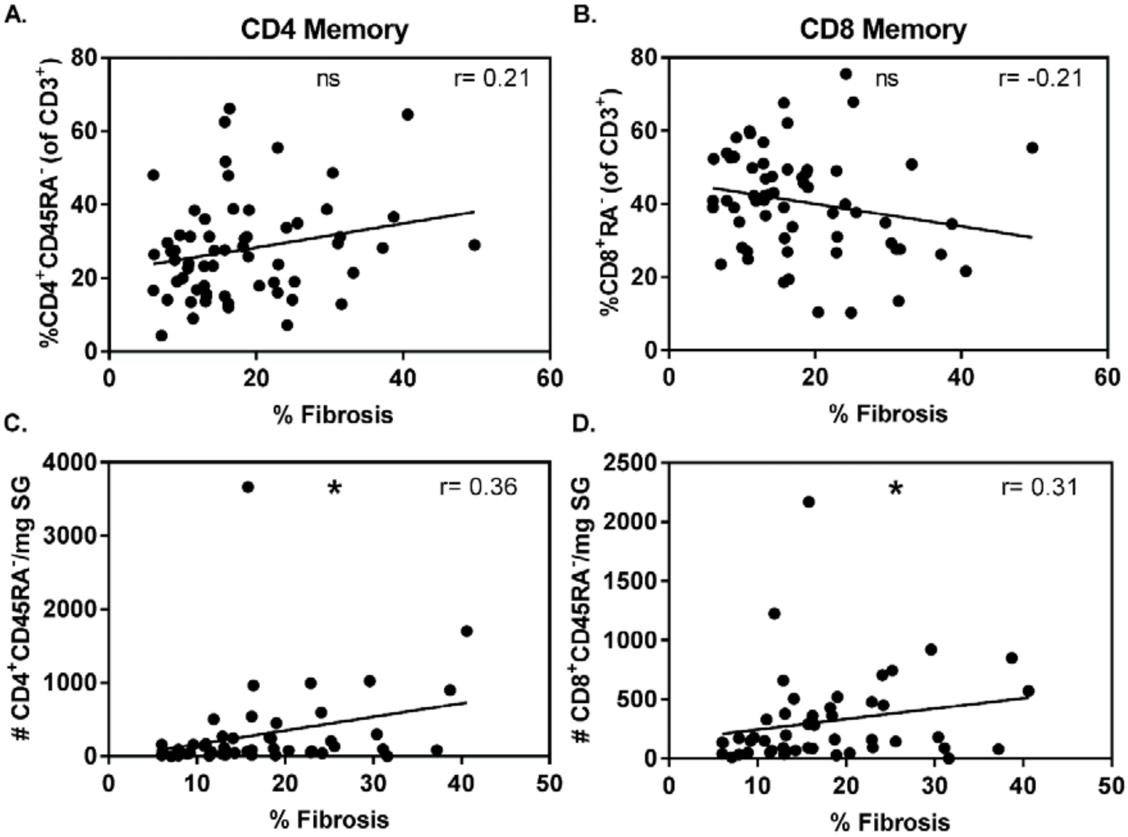

Figure S2. Salivary gland fibrosis is correlated with numbers, but not proportions of SG tissue resident T cells. Correlation of proportions of memory CD4 (**A**) and CD8 (**B**) salivary gland T cells with degree of salivary grand fibrosis (pSS, n=32 and nSS, n=30). Correlations of absolute numbers of memory CD4 (**C**) and CD8 (**D**) salivary gland T cells/mg biopsy tissue with degree of salivary gland fibrosis (pSS, n=22 and nSS, n=27). Spearman's two-tailed tests were used for all analyses. *p<0.05, ns = p ≥0.5.

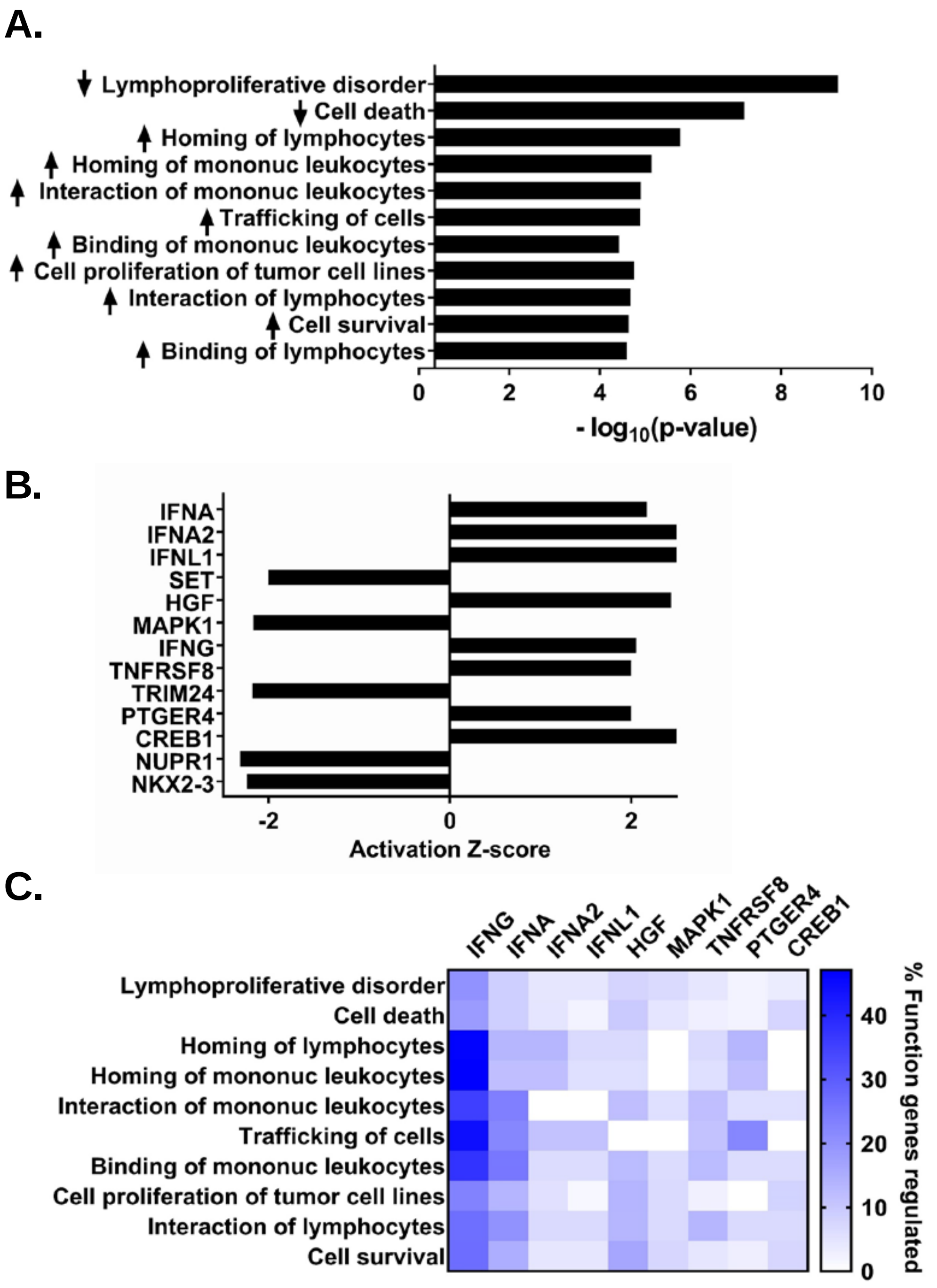

Figure S3. Functions and upstream drivers predicted by Ingenuity Pathways Analysis (IPA). (**A**) IPA predicted Diseases and Functions at p<0.0001 significance. Up and down arrows denote activation Z scores >2.0 or <-2.0, indicating predicted up- or down-regulated activity, respectively. (**B**) Activation Z-scores of Ingenuity protein-encoding predicted upstream regulators in order of significance (top to bottom). (**C**) Heatmap showing the percentage of differentially expressed Ingenuity Diseases and Function genes known to be regulated by the predicted upstream regulators listed at the top of the panel. Regulators influencing at least 5% of genes in any pathway are shown.

Table S1. Clinical Exam Values

Clinical Test		FACS Study pSS (n=51)	FACS Study nSS (n=69)	FACS Study p-value[a]	Microarray Study pSS (n=17)	Microarray Study nSS (n=15)	Microarray Study p-value[a]
Focus Score	Score, Med[b] (Range)	1.8 (0-12)	0 (0-2)	**<0.0001**	2.8 (1.1-12)	0 (0-0)	**<0.0001**
Anti-Ro[c]	Avg ± SEM	3.8 ± 0.52	0.39 ± 0.12	**<0.0001**	4.5 ± 0.87	0.2 ± 0	**<0.0001**
Anti-La[c]	Avg ± SEM	1.4 ± 0.32	0.28 ± 0.07	**<0.0001**	0.96 ± 0.49	0.2 ± 0	**0.019**
WUSF[d]	mL, Med (Range)	1.2 (0.001-11.8)	1.6 (0.005-16.9)	0.19	1.3 (0.01-11.7)	2.5 (0.74-7.1)	0.062
Avg %[e] Fibrosis	% area, Med (Range)	22.6 (6.0-49.7)	13.2 (6.0-31.1)	**0.0005[f]**	16.4 (6.0-40.6)	12.9 (6.0-18.9)	**0.019**
Schirmer's score	mm, Med (Range)	9 (0-35)	15 (0-35)	**0.04**	15 (2-35)	15 (0-35)	**0.69[f]**
vBS[g]	Score, Med (Range)	6 (0-9)	2 (0-9)	**<0.0001**	5 (0-9)	2 (0-8)	**0.011[f]**

[a] Mann Whitney U-test, 2-tailed unless otherwise indicated

[b] Med = median

[c] Bio-Rad Bioplex 2200 result

[d] WUSF = whole unstimulated salivary flow (mL/15 min)

[e] Fibrosis data available from n=32 pSS, n=30 nSS (FACS study) and n=15 pSS, n=14 nSS (microarray study)

[f] Unpaired Student's t test

[g] vBS = van Bijsterveld score, maximum value

Table S2. SG Memory T cell proportion associations with clinical features in pSS (n=51)

	$CD4^+CD45RA^-$ (% of $CD3^+$)			
	Pearson's correlation[a]		Gamma regression model	
Clinical Feature	r	p-value	Model	Variable p-value
vBS[b]	**0.3**	**0.034**	x + age	0.072
			x	0.043
Schirmer's test[c]	0.056	0.7	x + age	0.45
			x	0.7
WUSF[d]	0.042	0.77	x + age	0.37
			x	0.76
Biopsy Focus Score	**0.44**	**0.0013**	x + age	**0.0054**
			x	**0.0019**
Ro Ab Status[e]	**0.43**	**0.0017**	x + age	**0.011**
			x	**0.0055**
Serum IgG	**0.37**	**0.007**	x + age	**0.004**
			x	**0.0078**

[a] Pearson's two-tailed test

[b] vBS = van Bijsterveld score, maximum value

[c] Shirmer's test, minimal score

[d] WUSF = whole unstimulated salivary flow (mL/15 min)

[e] Bio-Rad Bioplex 2200 test outcome (positive/negative)

Table S3. Genes up-regulated in pSS salivary gland $CD3^+CD4^+CD45RA^-$ T cells

Gene Symbol	Log FC pSS/nSS	p-value	Adj p-value	Description
FCRL3	5.68	2.83E-09	1.25E-05	Fc receptor-like 3 (FCRL3)
CXCL13	7.18	6.86E-08	2.15E-04	chemokine (C-X-C motif) ligand 13 (CXCL13)
SMN2	3.67	1.29E-06	1.87E-03	survival of motor neuron 2, centromeric (SMN2)
SIRPG	3.44	1.36E-06	1.87E-03	signal-regulatory protein gamma (SIRPG)
C18orf25	3.53	2.13E-06	2.79E-03	chromosome 18 open reading frame 25 (C18orf25), transcript variant 1
FILIP1L	3.70	2.57E-06	3.23E-03	filamin A interacting protein 1-like (FILIP1L), transcript variant 1
C9orf156	4.45	2.66E-06	3.23E-03	chromosome 9 open reading frame 156 (C9orf156)
BRWD1	3.44	5.05E-06	5.68E-03	bromodomain and WD repeat domain containing 1 (BRWD1), transcript variant 3
BIRC2	4.22	5.27E-06	5.72E-03	baculoviral IAP repeat containing 2 (BIRC2)
CD200	4.87	5.51E-06	5.78E-03	CD200 molecule (CD200), transcript variant 2
LOC100129936	4.42	7.39E-06	7.50E-03	cDNA FLJ42830 fis, clone BRCAN2017905
PVT1	3.74	9.19E-06	8.82E-03	Pvt1 oncogene (non-protein coding) (PVT1)
FLJ44715	4.15	9.45E-06	8.82E-03	cDNA FLJ44715 fis, clone BRACE3021430
COX16	3.67	9.52E-06	8.82E-03	COX16 cytochrome c oxidase assembly homolog (S. cerevisiae) (COX16), nuclear gene encoding mitochondrial protein, transcript variant 1
lincRNA:chr4:89637527-89646627_R	3.98	1.00E-05	9.03E-03	lincRNA:chr4:89637527-89646627 reverse strand
IFITM1	4.37	1.15E-05	1.00E-02	interferon induced transmembrane protein 1 (9-27) (IFITM1)
lincRNA:chr3:32508871-32518521_R	4.57	1.32E-05	1.10E-02	lincRNA:chr3:32508871-32518521 reverse strand
PDS5A	3.80	1.33E-05	1.10E-02	PDS5, regulator of cohesion maintenance, homolog A (S. cerevisiae) (PDS5A), transcript variant 1
PPP2CA	4.33	1.61E-05	1.27E-02	protein phosphatase 2, catalytic subunit, alpha isozyme (PPP2CA)
PLAC8	3.91	1.79E-05	1.28E-02	placenta-specific 8 (PLAC8), transcript variant 2
CSNK1G3	3.79	2.13E-05	1.40E-02	casein kinase 1, gamma 3 (CSNK1G3), transcript variant 4
MDN1	3.46	2.37E-05	1.52E-02	MDN1, midasin homolog (yeast) (MDN1)
ENST00000422938	3.32	2.54E-05	1.60E-02	TESTI2 Homo sapiens cDNA clone TESTI2044585 5'
PASK	4.92	2.96E-05	1.77E-02	PAS domain containing serine/threonine kinase (PASK), transcript variant 2
CXCR5	4.28	2.98E-05	1.77E-02	chemokine (C-X-C motif) receptor 5 (CXCR5), transcript variant 2
NR3C1	3.72	3.24E-05	1.77E-02	nuclear receptor subfamily 3, group C, member 1 (glucocorticoid receptor) (NR3C1), transcript variant 5
EPSTI1	4.70	3.24E-05	1.77E-02	epithelial stromal interaction 1 (breast) (EPSTI1), transcript variant 2
VOPP1	4.41	3.26E-05	1.77E-02	vesicular, overexpressed in cancer, prosurvival protein 1 (VOPP1)
HERC2P2	3.53	3.60E-05	1.89E-02	hect domain and RLD 2 pseudogene 2 (HERC2P2)
APH1A	4.10	3.71E-05	1.89E-02	anterior pharynx defective 1 homolog A (C. elegans) (APH1A), transcript variant 2
RNF19A	3.72	3.78E-05	1.89E-02	ring finger protein 19A (RNF19A), transcript variant 1
DAPP1	4.09	4.00E-05	1.93E-02	dual adaptor of phosphotyrosine and 3-phosphoinositides (DAPP1)
LOC100509498	3.06	4.04E-05	1.93E-02	hypothetical LOC100509498 (LOC100509498)
DHFR	3.66	4.06E-05	1.93E-02	dihydrofolate reductase (DHFR)
FEZ2	4.05	4.44E-05	2.00E-02	fasciculation and elongation protein zeta 2 (zygin II) (FEZ2), transcript variant 2
ARIH1	3.65	4.92E-05	2.13E-02	ariadne homolog, ubiquitin-conjugating enzyme E2 binding protein, 1 (Drosophila) (ARIH1)
SKIV2L2	3.54	4.94E-05	2.13E-02	superkiller viralicidic activity 2-like 2 (S. cerevisiae) (SKIV2L2)
lincRNA:chr6:158658627-158666616_F	3.82	5.08E-05	2.16E-02	lincRNA:chr6:158658627-158666616 forward strand
TSTD1	3.57	5.28E-05	2.22E-02	thiosulfate sulfurtransferase (rhodanese)-like domain containing 1 (TSTD1), transcript variant 1
lincRNA:chr8:129108594-129113502_R	3.85	5.65E-05	2.23E-02	lincRNA:chr8:129108594-129113502 reverse strand
SATB1	4.18	5.68E-05	2.23E-02	SATB homeobox 1 (SATB1), transcript variant 1
TCF7	4.20	5.74E-05	2.23E-02	transcription factor 7 (T-cell specific, HMG-box) (TCF7), transcript variant 1
TIGIT	3.93	5.80E-05	2.23E-02	T cell immunoreceptor with Ig and ITIM domains (TIGIT)
ENST00000390622	3.30	6.04E-05	2.23E-02	immunoglobulin heavy variable 1-46
ZBTB24	4.39	6.19E-05	2.23E-02	zinc finger and BTB domain containing 24 (ZBTB24), transcript variant 1
SHPRH	3.52	7.19E-05	2.41E-02	SNF2 histone linker PHD RING helicase (SHPRH), transcript variant 2
GNL3	3.02	7.61E-05	2.50E-02	guanine nucleotide binding protein-like 3 (nucleolar) (GNL3), transcript variant 1
MKRN7P	3.63	7.96E-05	2.51E-02	makorin ring finger protein 7, pseudogene (MKRN7P)
lincRNA:chrX:73425071-73428011_R	4.38	8.43E-05	2.63E-02	lincRNA:chrX:73425071-73428011 reverse strand
DGKH	3.99	9.08E-05	2.70E-02	diacylglycerol kinase, eta (DGKH), transcript variant 1
LIMS3L	3.57	9.31E-05	2.74E-02	LIM and senescent cell antigen-like domains 3-like (LIMS3L)
LOC100133991	4.43	9.69E-05	2.75E-02	uncharacterized LOC100133991 (LOC100133991)
IL6ST	3.13	1.03E-04	2.86E-02	interleukin 6 signal transducer (gp130, oncostatin M receptor) (IL6ST), transcript variant 3

SETD1B	4.35	1.05E-04	2.88E-02	SET domain containing 1B (SETD1B)
SPRY1	3.94	1.06E-04	2.88E-02	sprouty homolog 1, antagonist of FGF signaling (Drosophila) (SPRY1), transcript variant 2
BABAM1	4.00	1.08E-04	2.89E-02	BRISC and BRCA1 A complex member 1 (BABAM1), transcript variant 1
lincRNA:chr11:130730240-130745336_R	2.63	1.16E-04	2.94E-02	lincRNA:chr11:130730240-130745336 reverse strand
MRPS5	4.62	1.16E-04	2.94E-02	mitochondrial ribosomal protein S5 (MRPS5), nuclear gene encoding mitochondrial protein
MIAT	3.97	1.16E-04	2.94E-02	myocardial infarction associated transcript (non-protein coding) (MIAT)
A_33_P3404759	2.76	1.23E-04	2.95E-02	Unknown
MAML2	3.06	1.23E-04	2.95E-02	mastermind-like 2 (Drosophila) (MAML2)
PDE4DIP	4.10	1.26E-04	2.95E-02	phosphodiesterase 4D interacting protein (PDE4DIP), transcript variant 9
PLEKHG2	3.31	1.26E-04	2.95E-02	pleckstrin homology domain containing, family G (with RhoGef domain) member 2 (PLEKHG2)
COX7B	4.24	1.27E-04	2.95E-02	cytochrome c oxidase subunit VIIb (COX7B), nuclear gene encoding mitochondrial protein
ZACN	3.87	1.28E-04	2.95E-02	zinc activated ligand-gated ion channel (ZACN)
KLHL12	2.96	1.37E-04	3.03E-02	kelch-like 12 (Drosophila) (KLHL12)
KLHL18	3.37	1.37E-04	3.03E-02	kelch-like 18 (Drosophila) (KLHL18)
UBA7	3.88	1.38E-04	3.03E-02	ubiquitin-like modifier activating enzyme 7 (UBA7)
VWA5A	3.73	1.42E-04	3.08E-02	von Willebrand factor A domain containing 5A (VWA5A), transcript variant 2
RASA4	3.57	1.46E-04	3.14E-02	RAS p21 protein activator 4 (RASA4)
RASSF2	4.58	1.47E-04	3.14E-02	Ras association (RalGDS/AF-6) domain family member 2 (RASSF2), transcript variant 1
TPP1	3.74	1.47E-04	3.14E-02	tripeptidyl peptidase I (TPP1)
NEDD9	3.97	1.51E-04	3.17E-02	neural precursor cell expressed, developmentally down-regulated 9 (NEDD9), transcript variant 2
CUL4B	3.35	1.53E-04	3.18E-02	cullin 4B (CUL4B), transcript variant 1
KIAA1671	3.40	1.54E-04	3.18E-02	KIAA1671 (KIAA1671)
UBAP2L	3.52	1.57E-04	3.22E-02	ubiquitin associated protein 2-like (UBAP2L), transcript variant 2
A_24_P32215	3.06	1.59E-04	3.23E-02	Unknown
PDE3B	3.32	1.67E-04	3.29E-02	phosphodiesterase 3B, cGMP-inhibited (PDE3B)
CCDC127	3.29	1.72E-04	3.36E-02	coiled-coil domain containing 127 (CCDC127)
GNL3L	3.83	1.76E-04	3.41E-02	guanine nucleotide binding protein-like 3 (nucleolar)-like (GNL3L), transcript variant 2
METTL23	3.78	1.88E-04	3.53E-02	methyltransferase like 23 (METTL23), transcript variant 6
X57723	3.26	1.98E-04	3.67E-02	rearranged TCR Vbeta 16a mRNA for T cell receptor
CRLS1	3.33	1.99E-04	3.67E-02	cardiolipin synthase 1 (CRLS1), transcript variant 1
GTF3C2	4.15	2.03E-04	3.69E-02	general transcription factor IIIC, polypeptide 2, beta 110kDa (GTF3C2), transcript variant 1
lincRNA:chr8:129108840-129113465_R	3.14	2.13E-04	3.76E-02	lincRNA:chr8:129108840-129113465 reverse strand
lincRNA:chr13:27831250-27836500_F	3.94	2.15E-04	3.78E-02	lincRNA:chr13:27831250-27836500 forward strand
DYNLL1	4.81	2.23E-04	3.85E-02	dynein, light chain, LC8-type 1 (DYNLL1), transcript variant 1
PRR14L	2.96	2.24E-04	3.85E-02	proline rich 14-like (PRR14L)
TAF7	3.73	2.29E-04	3.85E-02	TAF7 RNA polymerase II, TATA box binding protein (TBP)-associated factor, 55kDa (TAF7)
IRF9	3.94	2.30E-04	3.85E-02	interferon regulatory factor 9 (IRF9)
ATRN	3.15	2.43E-04	3.93E-02	attractin (ATRN), transcript variant 2
RAB12	4.11	2.45E-04	3.93E-02	RAB12, member RAS oncogene family (RAB12)
DCTD	4.00	2.49E-04	3.93E-02	dCMP deaminase (DCTD), transcript variant 1
LOC100133315	3.78	2.51E-04	3.93E-02	transient receptor potential cation channel, subfamily C, member 2-like (LOC100133315)
EIF3G	3.52	2.66E-04	4.09E-02	eukaryotic translation initiation factor 3, subunit G (EIF3G)
LDLRAP1	3.94	2.76E-04	4.18E-02	low density lipoprotein receptor adaptor protein 1 (LDLRAP1)
SELL	2.95	2.82E-04	4.22E-02	selectin L (SELL), transcript variant 1
SNIP1	3.07	2.85E-04	4.22E-02	Smad nuclear interacting protein 1 (SNIP1)
NAB1	3.67	2.85E-04	4.22E-02	NGFI-A binding protein 1 (EGR1 binding protein 1) (NAB1)
FKBP3	3.72	2.86E-04	4.22E-02	FK506 binding protein 3, 25kDa (FKBP3)
GNA13	3.64	2.88E-04	4.24E-02	guanine nucleotide binding protein (G protein), alpha 13 (GNA13)
PGAP1	3.51	2.92E-04	4.28E-02	post-GPI attachment to proteins 1 (PGAP1)
PIK3CD	3.17	2.95E-04	4.29E-02	phosphoinositide-3-kinase, catalytic, delta polypeptide (PIK3CD)
OAS2	3.22	3.18E-04	4.51E-02	2'-5'-oligoadenylate synthetase 2, 69/71kDa (OAS2), transcript variant 1
VBP1	4.39	3.18E-04	4.51E-02	von Hippel-Lindau binding protein 1 (VBP1)
lincRNA:chr8:129021903-129113336_R	2.64	3.24E-04	4.56E-02	lincRNA:chr8:129021903-129113336 reverse strand
A_33_P3376781	3.40	3.24E-04	4.56E-02	Unknown
lincRNA:chr8:129108828-129113500_R	2.61	3.28E-04	4.57E-02	lincRNA:chr8:129108828-129113500 reverse strand

THC2772510	3.01	3.33E-04	4.60E-02	GLT8D3 protein (Fragment), partial (91%)
lincRNA:chr20:1646825-1667675_F	2.89	3.43E-04	4.68E-02	lincRNA:chr20:1646825-1667675 forward strand
UCKL1	4.27	3.43E-04	4.68E-02	uridine-cytidine kinase 1-like 1 (UCKL1), transcript variant 1
ENST00000390633	2.83	3.49E-04	4.71E-02	immunoglobulin heavy variable 1-69
NF1P2	2.69	3.60E-04	4.77E-02	neurofibromin 1 pseudogene 2 (NF1P2)
AB360850	3.87	3.70E-04	4.81E-02	mRNA for T cell receptor beta variable 7, partial cds, clone: non 379
TERF1	3.24	3.75E-04	4.82E-02	telomeric repeat binding factor (NIMA-interacting) 1 (TERF1), transcript variant 1
PPP4R2	3.56	3.90E-04	4.97E-02	rotein phosphatase 4, regulatory subunit 2 (PPP4R2)
FOXO1	2.62	3.92E-04	4.98E-02	forkhead box O1 (FOXO1)
CASK	3.12	3.94E-04	4.98E-02	alcium/calmodulin-dependent serine protein kinase (MAGUK family) (CASK), transcript variant 1
FAM184B	3.40	3.98E-04	4.99E-02	family with sequence similarity 184, member B (FAM184B)
RAPGEF2	2.79	4.07E-04	5.07E-02	Rap guanine nucleotide exchange factor (GEF) 2 (RAPGEF2)
NECAP2	3.81	4.14E-04	5.07E-02	NECAP endocytosis associated 2 (NECAP2), transcript variant 1
COMMD3	2.83	4.17E-04	5.07E-02	COMM domain containing 3 (COMMD3)
PSMD12	3.33	4.20E-04	5.07E-02	proteasome (prosome, macropain) 26S subunit, non-ATPase, 12 (PSMD12), transcript variant 1
EIF4EBP2	2.91	4.39E-04	5.21E-02	eukaryotic translation initiation factor 4E binding protein 2 (EIF4EBP2)
ZC3H8	2.28	4.43E-04	5.21E-02	zinc finger CCCH-type containing 8 (ZC3H8)
MKI67IP	2.96	4.44E-04	5.21E-02	MKI67 (FHA domain) interacting nucleolar phosphoprotein (MKI67IP)
FAIM3	2.25	4.52E-04	5.25E-02	Fas apoptotic inhibitory molecule 3 (FAIM3), transcript variant 1
TRIM5	3.95	4.59E-04	5.28E-02	tripartite motif containing 5 (TRIM5), transcript variant gamma
TOMM6	2.89	4.62E-04	5.29E-02	translocase of outer mitochondrial membrane 6 homolog (yeast) (TOMM6)
ESD	4.44	4.67E-04	5.33E-02	esterase D (ESD)
DKC1	2.93	4.69E-04	5.33E-02	dyskeratosis congenita 1, dyskerin (DKC1), transcript variant 1
GDE1	3.31	4.73E-04	5.36E-02	glycerophosphodiester phosphodiesterase 1 (GDE1)
LOC441455	2.78	4.77E-04	5.36E-02	makorin ring finger protein 1 pseudogene (LOC441455)
A_33_P3357964	2.70	4.77E-04	5.36E-02	Unknown
PARP9	3.64	4.80E-04	5.36E-02	poly (ADP-ribose) polymerase family, member 9 (PARP9)
ARFIP1	2.92	4.80E-04	5.36E-02	ADP-ribosylation factor interacting protein 1 (ARFIP1), transcript variant 1
RBL1	3.41	4.84E-04	5.36E-02	retinoblastoma-like 1 (p107) (RBL1), transcript variant 2
RPA2	3.98	4.85E-04	5.36E-02	replication protein A2, 32kDa (RPA2)
DNAH17	3.28	4.91E-04	5.36E-02	dynein, axonemal, heavy chain 17 (DNAH17)
ENST00000390610	3.25	4.92E-04	5.36E-02	immunoglobulin heavy variable 1-24
CKS1B	3.69	4.97E-04	5.39E-02	CDC28 protein kinase regulatory subunit 1B (CKS1B)
A_33_P3424108	2.65	5.10E-04	5.50E-02	Unknown
ERP44	3.08	5.13E-04	5.50E-02	endoplasmic reticulum protein 44 (ERP44)
DDX42	2.96	5.15E-04	5.50E-02	DEAD (Asp-Glu-Ala-Asp) box polypeptide 42 (DDX42), transcript variant 1
LONP1	2.67	5.20E-04	5.50E-02	lon peptidase 1, mitochondrial (LONP1), nuclear gene encoding mitochondrial protein
SNX27	3.99	5.39E-04	5.64E-02	sorting nexin family member 27 (SNX27)
MOCS1	2.76	5.41E-04	5.64E-02	molybdenum cofactor synthesis 1 (MOCS1)
OSBP	3.48	5.52E-04	5.74E-02	oxysterol binding protein (OSBP)
UROS	2.91	5.60E-04	5.76E-02	uroporphyrinogen III synthase (UROS)
lincRNA:chr15:69951596-69988646_F	2.41	5.67E-04	5.81E-02	lincRNA:chr15:69951596-69988646 forward strand
CSF1R	1.97	5.70E-04	5.82E-02	colony stimulating factor 1 receptor (CSF1R)
RABGAP1L	3.94	5.71E-04	5.82E-02	RAB GTPase activating protein 1-like (RABGAP1L)
SKP2	2.69	5.74E-04	5.83E-02	S-phase kinase-associated protein 2 (p45) (SKP2)
GLS	4.51	5.86E-04	5.91E-02	glutaminase (GLS), nuclear gene encoding mitochondrial protein
LOC100506190	4.05	5.93E-04	5.94E-02	Alu subfamily SC sequence contamination warning entry
CHN1	3.68	5.98E-04	5.94E-02	chimerin (chimaerin) 1 (CHN1), transcript variant 1
GGCT	2.47	6.00E-04	5.94E-02	gamma-glutamylcyclotransferase (GGCT)
NF1	2.88	6.34E-04	6.22E-02	neurofibromin 1 (NF1), transcript variant 2
NONO	2.52	6.48E-04	6.26E-02	non-POU domain containing, octamer-binding (NONO)
RAB3GAP1	3.28	6.49E-04	6.26E-02	RAB3 GTPase activating protein subunit 1 (catalytic) (RAB3GAP1), transcript variant 2
GCC2	3.26	6.51E-04	6.26E-02	GRIP and coiled-coil domain containing 2 (GCC2), transcript variant 1
lincRNA:chr5:43014835-43017310_F	2.78	6.52E-04	6.26E-02	lincRNA:chr5:43014835-43017310 forward strand
PHKB	3.97	6.54E-04	6.26E-02	phosphorylase kinase, beta (PHKB), transcript variant 2

CMAHP	3.62	6.56E-04	6.26E-02	cytidine monophospho-N-acetylneuraminic acid hydroxylase, pseudogene (CMAHP), transcript variant 1
FAM86B2	3.39	6.63E-04	6.26E-02	family with sequence similarity 86, member B2 (FAM86B2)
PHF3	3.58	6.76E-04	6.30E-02	PHD finger protein 3 (PHF3)
BUB3	2.71	6.94E-04	6.37E-02	budding uninhibited by benzimidazoles 3 homolog (yeast) (BUB3)
lincRNA:chr8:129015583-129108805_F	2.46	7.01E-04	6.39E-02	lincRNA:chr8:129015583-129108805 forward strand
lincRNA:chr22:25588075-25594350_R	2.01	7.12E-04	6.46E-02	lincRNA:chr22:25588075-25594350 reverse strand
MCFD2	3.46	7.17E-04	6.47E-02	multiple coagulation factor deficiency 2 (MCFD2), transcript variant 1
SSRP1	4.29	7.28E-04	6.52E-02	structure specific recognition protein 1 (SSRP1)
CCDC75	3.81	7.32E-04	6.52E-02	coiled-coil domain containing 75 (CCDC75)
PDXDC1	3.05	7.32E-04	6.52E-02	pyridoxal-dependent decarboxylase domain containing 1 (PDXDC1)
PPP1CC	3.51	7.34E-04	6.52E-02	protein phosphatase 1, catalytic subunit, gamma isozyme (PPP1CC), transcript variant 1
COPB2	2.60	7.41E-04	6.53E-02	coatomer protein complex, subunit beta 2 (beta prime) (COPB2), transcript variant 1
APAF1	2.77	7.44E-04	6.53E-02	apoptotic peptidase activating factor 1 (APAF1), transcript variant 3
SNORA23	3.39	7.46E-04	6.53E-02	small nucleolar RNA, H/ACA box 23 (SNORA23), small nucleolar RNA [NR_002962]
SYNJ1	2.98	7.50E-04	6.54E-02	synaptojanin 1 (SYNJ1), transcript variant 2
TAB2	2.54	7.53E-04	6.55E-02	TGF-beta activated kinase 1/MAP3K7 binding protein 2 (TAB2)
ZNF827	2.87	7.58E-04	6.56E-02	zinc finger protein 827 (ZNF827)
HSDL1	3.64	7.62E-04	6.57E-02	hydroxysteroid dehydrogenase like 1 (HSDL1), transcript variant 1
TARS2	4.45	7.65E-04	6.58E-02	threonyl-tRNA synthetase 2, mitochondrial (putative) (TARS2)
CCNL1	1.85	7.81E-04	6.67E-02	cyclin L1 (CCNL1)
DNMT3A	2.32	7.95E-04	6.74E-02	DNA (cytosine-5-)-methyltransferase 3 alpha (DNMT3A), transcript variant 1
lincRNA:chrX:73470227-73470871_R	2.05	7.96E-04	6.74E-02	lincRNA:chrX:73470227-73470871 reverse strand
S1PR1	3.89	8.01E-04	6.74E-02	sphingosine-1-phosphate receptor 1 (S1PR1)
ORF1	2.73	8.04E-04	6.74E-02	partial mRNA for hypothetical protein (ORF1), clone pT-Adv JuaX22
TPP2	2.54	8.06E-04	6.74E-02	tripeptidyl peptidase II (TPP2)
lincRNA:chr3:47207196-47217796_F	2.96	8.11E-04	6.74E-02	lincRNA:chr3:47207196-47217796 forward strand
DBF4	3.28	8.23E-04	6.82E-02	DBF4 homolog (S. cerevisiae) (DBF4)
DYNC1LI2	3.12	8.52E-04	6.99E-02	dynein, cytoplasmic 1, light intermediate chain 2 (DYNC1LI2)
CCR7	2.09	8.53E-04	6.99E-02	chemokine (C-C motif) receptor 7 (CCR7)
FANCD2	2.58	8.66E-04	6.99E-02	Fanconi anemia, complementation group D2 (FANCD2), transcript variant 2
MRPL33	3.37	8.69E-04	6.99E-02	mitochondrial ribosomal protein L33 (MRPL33), nuclear gene encoding mitochondrial protein, transcript variant 1
ENOSF1	3.08	8.69E-04	6.99E-02	enolase superfamily member 1 (ENOSF1), transcript variant 2
ARHGEF18	3.12	8.72E-04	6.99E-02	Rho/Rac guanine nucleotide exchange factor (GEF) 18 (ARHGEF18), transcript variant 1
NLRC5	3.15	8.73E-04	6.99E-02	NLR family, CARD domain containing 5 (NLRC5)
POLB	1.96	8.84E-04	6.99E-02	polymerase (DNA directed), beta (POLB)
GRAMD1B	3.01	8.86E-04	6.99E-02	GRAM domain containing 1B (GRAMD1B)
RASSF5	4.12	8.87E-04	6.99E-02	Ras association (RalGDS/AF-6) domain family member 5 (RASSF5), transcript variant 2
ENST00000540599	3.09	8.87E-04	6.99E-02	cDNA FLJ36021 fis, clone TESTI2016568
lincRNA:chr8:129082331-129113343_F	2.70	8.91E-04	6.99E-02	lincRNA:chr8:129082331-129113343 forward strand
P2RY10	3.42	9.18E-04	7.09E-02	purinergic receptor P2Y, G-protein coupled, 10 (P2RY10), transcript variant 1
FAM115C	2.65	9.24E-04	7.10E-02	family with sequence similarity 115, member C (FAM115C), transcript variant 3
CTLA4	2.82	9.29E-04	7.11E-02	cytotoxic T-lymphocyte-associated protein 4 (CTLA4), transcript variant 1
GGCX	2.63	9.34E-04	7.11E-02	gamma-glutamyl carboxylase (GGCX), transcript variant 1
RBM8A	2.14	9.47E-04	7.16E-02	RNA binding motif protein 8A (RBM8A)
LRRC16A	2.46	9.51E-04	7.16E-02	leucine rich repeat containing 16A (LRRC16A), transcript variant 1
COG3	3.25	9.54E-04	7.16E-02	component of oligomeric golgi complex 3 (COG3)
PRKAR1A	3.81	9.55E-04	7.16E-02	protein kinase, cAMP-dependent, regulatory, type I, alpha (tissue specific extinguisher 1) (PRKAR1A), transcript variant 3
lincRNA:chr5:74327969-74348269_F	3.35	9.62E-04	7.16E-02	lincRNA:chr5:74327969-74348269 forward strand
RPL19	3.36	9.70E-04	7.16E-02	ribosomal protein L19 (RPL19)
AKAP8	3.29	9.81E-04	7.16E-02	A kinase (PRKA) anchor protein 8 (AKAP8
GALM	3.87	9.81E-04	7.16E-02	galactose mutarotase (aldose 1-epimerase) (GALM)
C4orf41	2.62	9.82E-04	7.16E-02	chromosome 4 open reading frame 41 (C4orf41), transcript variant 2
CD302	3.21	9.85E-04	7.16E-02	CD302 molecule (CD302), transcript variant 1
EEF2	4.07	9.93E-04	7.16E-02	eukaryotic translation elongation factor 2 (EEF2)
PSMC6	3.07	9.94E-04	7.16E-02	proteasome (prosome, macropain) 26S subunit, ATPase, 6 (PSMC6)

A_33_P3240966	2.71	9.95E-04	7.16E-02	Unknown
ENST00000391369	2.52	1.00E-03	7.18E-02	HCG2032337PRO1848 Uncharacterized protein
NDFIP2	2.66	1.01E-03	7.21E-02	Nedd4 family interacting protein 2 (NDFIP2), transcript variant 1
UBE3A	2.40	1.06E-03	7.41E-02	ubiquitin protein ligase E3A (UBE3A), transcript variant 3
RILPL2	3.40	1.06E-03	7.41E-02	Rab interacting lysosomal protein-like 2 (RILPL2)
lincRNA:chr2:67037871-67048646_F	2.17	1.06E-03	7.41E-02	lincRNA:chr2:67037871-67048646 forward strand
lincRNA:chr22:27062378-27064552_R	2.70	1.06E-03	7.41E-02	lincRNA:chr22:27062378-27064552 reverse strand
DDHD1	3.03	1.07E-03	7.41E-02	DDHD domain containing 1 (DDHD1), transcript variant 1
HIC2	2.50	1.08E-03	7.41E-02	hypermethylated in cancer 2 (HIC2)
PLAT	2.84	1.08E-03	7.41E-02	plasminogen activator, tissue (PLAT), transcript variant 1
RICTOR	2.70	1.08E-03	7.41E-02	RPTOR independent companion of MTOR, complex 2 (RICTOR)
SNX3	3.38	1.09E-03	7.41E-02	sorting nexin 3 (SNX3), transcript variant 1
ENST00000390595	3.35	1.09E-03	7.41E-02	immunoglobulin heavy variable 1-3
OR1L4	2.16	1.09E-03	7.41E-02	olfactory receptor, family 1, subfamily L, member 4 (OR1L4)
LIMS1	2.98	1.10E-03	7.41E-02	LIM and senescent cell antigen-like domains 1 (LIMS1), transcript variant 2
CEP68	3.12	1.10E-03	7.41E-02	centrosomal protein 68kDa (CEP68)
SF3B3	3.91	1.10E-03	7.41E-02	splicing factor 3b, subunit 3, 130kDa (SF3B3)
ADI1	2.95	1.10E-03	7.41E-02	acireductone dioxygenase 1 (ADI1)
NOD2	3.42	1.10E-03	7.41E-02	nucleotide-binding oligomerization domain containing 2 (NOD2)
lincRNA:chr6:29701971-29740296_F	3.27	1.10E-03	7.41E-02	lincRNA:chr6:29701971-29740296 forward strand
ATF7IP	2.94	1.11E-03	7.41E-02	activating transcription factor 7 interacting protein (ATF7IP)
ZNF410	2.56	1.12E-03	7.41E-02	zinc finger protein 410 (ZNF410), transcript variant 2
AGPS	3.42	1.12E-03	7.41E-02	alkylglycerone phosphate synthase (AGPS)
CBX7	3.59	1.12E-03	7.41E-02	chromobox homolog 7 (CBX7)
PPFIA1	3.26	1.14E-03	7.50E-02	protein tyrosine phosphatase, receptor type, f polypeptide (PTPRF), interacting protein (liprin), alpha 1 (PPFIA1), transcript variant 2
EDEM1	3.36	1.16E-03	7.54E-02	ER degradation enhancer, mannosidase alpha-like 1 (EDEM1)
MCM6	2.92	1.18E-03	7.60E-02	minichromosome maintenance complex component 6 (MCM6)
RBM18	3.37	1.18E-03	7.61E-02	RNA binding motif protein 18 (RBM18), transcript variant 1
ALG6	2.63	1.18E-03	7.61E-02	asparagine-linked glycosylation 6, alpha-1,3-glucosyltransferase homolog (S. cerevisiae) (ALG6)
ATP2C1	3.22	1.19E-03	7.62E-02	ATPase, Ca++ transporting, type 2C, member 1 (ATP2C1), transcript variant 3
SKIV2L	2.94	1.19E-03	7.62E-02	superkiller viralicidic activity 2-like (S. cerevisiae) (SKIV2L)
MRPS18A	2.57	1.20E-03	7.62E-02	mitochondrial ribosomal protein S18A (MRPS18A), nuclear gene encoding mitochondrial protein, transcript variant 1
NEK4	3.71	1.21E-03	7.67E-02	NIMA (never in mitosis gene a)-related kinase 4 (NEK4), transcript variant 1
A_33_P3376954	3.08	1.22E-03	7.70E-02	Unknown
RAB30	2.51	1.22E-03	7.70E-02	RAB30, member RAS oncogene family (RAB30)
AAK1	3.67	1.23E-03	7.70E-02	AP2 associated kinase 1 (AAK1)
RPL18	3.43	1.24E-03	7.73E-02	ribosomal protein L18 (RPL18)
ENST00000390639	1.79	1.24E-03	7.73E-02	immunoglobulin heavy variable 7-81 (non-functional)
DLEU1	2.77	1.24E-03	7.73E-02	deleted in lymphocytic leukemia 1 (non-protein coding) (DLEU1)
RBAK	2.90	1.24E-03	7.73E-02	RB-associated KRAB zinc finger (RBAK), transcript variant 1
LOC100507429	2.49	1.26E-03	7.78E-02	Homo sapiens hypothetical LOC100507429 (LOC100507429)
NAT10	2.94	1.26E-03	7.78E-02	N-acetyltransferase 10 (GCN5-related) (NAT10), transcript variant 1
lincRNA:chr8:129032934-129113447_R	2.60	1.27E-03	7.78E-02	lincRNA:chr8:129032934-129113447 reverse strand
ENST00000502284	2.40	1.27E-03	7.78E-02	cDNA FLJ44668 fis, clone BRACE3004981
JHDM1D	3.81	1.28E-03	7.78E-02	jumonji C domain containing histone demethylase 1 homolog D (S. cerevisiae) (JHDM1D)
NDUFS6	3.09	1.28E-03	7.78E-02	NADH dehydrogenase (ubiquinone) Fe-S protein 6, 13kDa (NADH-coenzyme Q reductase) (NDUFS6), nuclear gene encoding mitochondrial protein
C7orf42	3.09	1.29E-03	7.78E-02	chromosome 7 open reading frame 42 (C7orf42)
STT3A	3.32	1.29E-03	7.78E-02	STT3, subunit of the oligosaccharyltransferase complex, homolog A (S. cerevisiae) (STT3A)
CDYL2	2.81	1.29E-03	7.78E-02	chromodomain protein, Y-like 2 (CDYL2)
VAMP1	3.52	1.30E-03	7.83E-02	vesicle-associated membrane protein 1 (synaptobrevin 1) (VAMP1), transcript variant 3
UBN1	3.12	1.31E-03	7.86E-02	ubinuclein 1 (UBN1), transcript variant 1
XLOC_l2_014645	3.21	1.31E-03	7.86E-02	lincRNA (XLOC_l2_014645), lincRNA [TCONS_l2_00028399]
VNN2	1.86	1.32E-03	7.90E-02	vanin 2 (VNN2), transcript variant 1
ZZZ3	1.94	1.34E-03	7.94E-02	zinc finger, ZZ-type containing 3 (ZZZ3)
MRPL35	3.53	1.34E-03	7.94E-02	mitochondrial ribosomal protein L35 (MRPL35), nuclear gene encoding mitochondrial protein, transcript variant 2

IGFBP4	2.95	1.35E-03	7.95E-02	insulin-like growth factor binding protein 4 (IGFBP4)
TFRC	2.98	1.36E-03	7.99E-02	transferrin receptor (p90, CD71) (TFRC), transcript variant 1
TMEM194B	1.82	1.37E-03	8.01E-02	transmembrane protein 194B
TXK	2.75	1.38E-03	8.06E-02	TXK tyrosine kinase (TXK)
EVC	2.85	1.40E-03	8.15E-02	Ellis van Creveld syndrome (EVC)
OSBPL3	2.91	1.41E-03	8.15E-02	oxysterol binding protein-like 3 (OSBPL3), transcript variant 1
UQCRH	3.30	1.41E-03	8.15E-02	ubiquinol-cytochrome c reductase hinge protein (UQCRH), nuclear gene encoding mitochondrial protein
SEPN1	2.39	1.43E-03	8.20E-02	selenoprotein N, 1 (SEPN1), transcript variant 1
FBXL20	2.86	1.43E-03	8.20E-02	F-box and leucine-rich repeat protein 20 (FBXL20), transcript variant 1
lincRNA:chr14:22994620-23016487_F	3.03	1.44E-03	8.20E-02	lincRNA:chr14:22994620-23016487 forward strand
FAU	3.13	1.45E-03	8.20E-02	Finkel-Biskis-Reilly murine sarcoma virus (FBR-MuSV) ubiquitously expressed (FAU)
DCTN6	4.26	1.48E-03	8.30E-02	dynactin 6 (DCTN6)
IPO7	3.55	1.48E-03	8.31E-02	importin 7 (IPO7)
ADSL	3.12	1.49E-03	8.33E-02	adenylosuccinate lyase (ADSL), transcript variant 1
FBXW2	3.28	1.50E-03	8.33E-02	F-box and WD repeat domain containing 2 (FBXW2)
lincRNA:chr8:141532461-141536259_R	2.87	1.50E-03	8.37E-02	lincRNA:chr8:141532461-141536259 reverse strand
WTAP	2.50	1.51E-03	8.37E-02	Wilms tumor 1 associated protein
AK096917	3.16	1.54E-03	8.46E-02	cDNA FLJ39598 fis, clone SKNSH2002866
RAB4A	3.42	1.58E-03	8.63E-02	RAB4A, member RAS oncogene family (RAB4A)
ENST00000445752	2.88	1.61E-03	8.65E-02	coiled-coil domain containing 144A
lincRNA:chr3:9387975-9397375_R	2.82	1.62E-03	8.67E-02	lincRNA:chr3:9387975-9397375 reverse strand
C12orf23	3.10	1.64E-03	8.71E-02	chromosome 12 open reading frame 23 (C12orf23)
SEC11A	3.66	1.68E-03	8.82E-02	SEC11 homolog A (S. cerevisiae) (SEC11A)
C2orf3	2.44	1.71E-03	8.89E-02	chromosome 2 open reading frame 3 (C2orf3), transcript variant 1
MLF1	2.87	1.74E-03	8.94E-02	myeloid leukemia factor 1 (MLF1), transcript variant 1
LOC100506774	3.07	1.75E-03	8.94E-02	Homo sapiens hypothetical LOC100506774 (LOC100506774)
EIF4A3	3.43	1.75E-03	8.94E-02	eukaryotic translation initiation factor 4A3 (EIF4A3)
STAT1	3.37	1.76E-03	8.94E-02	signal transducer and activator of transcription 1, 91kDa (STAT1), transcript variant beta
TRIM14	2.00	1.76E-03	8.94E-02	tripartite motif containing 14 (TRIM14), transcript variant 2
ELK1	3.06	1.77E-03	8.94E-02	ELK1, member of ETS oncogene family (ELK1), transcript variant 2
LYPLA1	2.37	1.77E-03	8.94E-02	lysophospholipase I (LYPLA1)
MBIP	3.45	1.77E-03	8.94E-02	MAP3K12 binding inhibitory protein 1 (MBIP), transcript variant 2
CKAP2	3.08	1.78E-03	8.94E-02	cytoskeleton associated protein 2 (CKAP2), transcript variant 1
TLE3	1.87	1.78E-03	8.94E-02	transducin-like enhancer of split 3 (E(sp1) homolog, Drosophila) (TLE3), transcript variant 1
PRKCB	3.25	1.79E-03	8.94E-02	protein kinase C, beta (PRKCB), transcript variant 2
HSPA4	2.97	1.79E-03	8.94E-02	heat shock 70kDa protein 4 (HSPA4)
A_24_P255473	3.20	1.79E-03	8.94E-02	Unknown
SLC25A14	2.84	1.79E-03	8.94E-02	solute carrier family 25 (mitochondrial carrier, brain), member 14 (SLC25A14), nuclear gene encoding mitochondrial protein, transcript variant long
HDGF	2.69	1.79E-03	8.94E-02	hepatoma-derived growth factor (HDGF), transcript variant 1
RBCK1	2.50	1.80E-03	8.94E-02	RanBP-type and C3HC4-type zinc finger containing 1 (RBCK1), transcript variant 2,
USP3	3.35	1.81E-03	8.97E-02	ubiquitin specific peptidase 3 (USP3)
ENST00000390394	2.71	1.82E-03	8.97E-02	T cell receptor beta variable 20-1
A_33_P3385387	2.67	1.82E-03	8.97E-02	Unknown
PILRA	2.82	1.83E-03	8.99E-02	paired immunoglobin-like type 2 receptor alpha (PILRA), transcript variant 3
C10orf46	2.20	1.84E-03	9.04E-02	chromosome 10 open reading frame 46 (C10orf46)
C17orf49	3.00	1.87E-03	9.05E-02	chromosome 17 open reading frame 49 (C17orf49)
LOC100127885	2.46	1.87E-03	9.05E-02	hypothetical protein LOC100127885 (LOC100127885)
MAN1C1	2.72	1.87E-03	9.05E-02	mannosidase, alpha, class 1C, member 1 (MAN1C1)
SYNC	2.71	1.87E-03	9.05E-02	syncoilin, intermediate filament protein (SYNC), transcript variant 1
POLE	2.02	1.87E-03	9.05E-02	polymerase (DNA directed), epsilon (POLE)
IKBKE	3.43	1.88E-03	9.06E-02	inhibitor of kappa light polypeptide gene enhancer in B-cells, kinase epsilon (IKBKE), transcript variant 1
POLR3E	3.60	1.88E-03	9.06E-02	polymerase (RNA) III (DNA directed) polypeptide E (80kD) (POLR3E)
CDKAL1	2.99	1.89E-03	9.07E-02	CDK5 regulatory subunit associated protein 1-like 1 (CDKAL1)
lincRNA:chr8:129001411-129082607_R	3.10	1.91E-03	9.15E-02	lincRNA:chr8:129001411-129082607 reverse strand
BRMS1	2.86	1.92E-03	9.18E-02	breast cancer metastasis suppressor 1 (BRMS1), transcript variant 2

NECAP1	2.68	1.93E-03	9.20E-02	NECAP endocytosis associated 1 (NECAP1), transcript variant 1
ATP6V1A	2.84	1.94E-03	9.20E-02	ATPase, H+ transporting, lysosomal 70kDa, V1 subunit A (ATP6V1A)
ARHGAP35	2.78	1.94E-03	9.20E-02	Rho GTPase activating protein 35 (ARHGAP35)
GNAQ	2.41	1.96E-03	9.21E-02	guanine nucleotide binding protein (G protein), q polypeptide (GNAQ)
ENST00000435913	2.79	1.96E-03	9.21E-02	Unknown
TAB3	2.42	1.96E-03	9.21E-02	TGF-beta activated kinase 1/MAP3K7 binding protein 3 (TAB3)
FDPS	3.45	1.97E-03	9.21E-02	farnesyl diphosphate synthase (FDPS), transcript variant 1
CHD6	2.09	2.00E-03	9.27E-02	chromodomain helicase DNA binding protein 6 (CHD6)
A_33_P3362453	2.19	2.01E-03	9.30E-02	Unknown
lincRNA:chr12:68421607-68422081_F	2.43	2.01E-03	9.30E-02	lincRNA:chr12:68421607-68422081 forward strand
MAT2B	3.45	2.03E-03	9.30E-02	methionine adenosyltransferase II, beta (MAT2B), transcript variant 2
lincRNA:chr1:40004013-40020238_F	2.86	2.03E-03	9.30E-02	lincRNA:chr1:40004013-40020238 forward strand
C5orf56	3.06	2.03E-03	9.30E-02	cDNA: FLJ21568 fis, clone COL06492
CD2	3.07	2.03E-03	9.30E-02	cDNA, FLJ17718
DCAF13	3.45	2.03E-03	9.30E-02	DDB1 and CUL4 associated factor 13 (DCAF13)
PGAM1	2.94	2.04E-03	9.30E-02	phosphoglycerate mutase 1 (brain) (PGAM1)
GEM	3.86	2.04E-03	9.30E-02	GTP binding protein overexpressed in skeletal muscle (GEM), transcript variant 1
MGA	2.52	2.05E-03	9.31E-02	ref|Homo sapiens MAX gene associated (MGA), transcript variant 1
MEAF6	2.48	2.06E-03	9.31E-02	MYST/Esa1-associated factor 6 (MEAF6)
RHBDD2	3.34	2.07E-03	9.31E-02	rhomboid domain containing 2 (RHBDD2), transcript variant 2
ZCCHC17	3.61	2.07E-03	9.31E-02	zinc finger, CCHC domain containing 17 (ZCCHC17)
ILKAP	3.34	2.08E-03	9.34E-02	integrin-linked kinase-associated serine/threonine phosphatase (ILKAP)
CLASP1	2.72	2.09E-03	9.37E-02	cytoplasmic linker associated protein 1 (CLASP1), transcript variant 1
ETFA	2.86	2.09E-03	9.37E-02	electron-transfer-flavoprotein, alpha polypeptide (ETFA), nuclear gene encoding mitochondrial protein, transcript variant 1
RAB43	2.91	2.12E-03	9.45E-02	RAB43, member RAS oncogene family (RAB43), transcript variant 1
PDPK1	2.17	2.13E-03	9.46E-02	3-phosphoinositide dependent protein kinase-1 (PDPK1), transcript variant 1
ENST00000399576	3.08	2.14E-03	9.48E-02	Uncharacterized protein
ST6GAL1	2.79	2.15E-03	9.48E-02	T6 beta-galactosamide alpha-2,6-sialyltranferase 1 (ST6GAL1), transcript variant 1
NFKBID	3.13	2.18E-03	9.53E-02	nuclear factor of kappa light polypeptide gene enhancer in B-cells inhibitor, delta (NFKBID)
ELMO1	3.13	2.19E-03	9.53E-02	engulfment and cell motility 1 (ELMO1), transcript variant 1
AGFG1	3.13	2.19E-03	9.53E-02	ArfGAP with FG repeats 1 (AGFG1), transcript variant 1
VMP1	2.54	2.19E-03	9.53E-02	vacuole membrane protein 1 (VMP1)
C1orf124	2.51	2.23E-03	9.63E-02	chromosome 1 open reading frame 124 (C1orf124), transcript variant 1
FAM20B	2.42	2.24E-03	9.63E-02	family with sequence similarity 20, member B (FAM20B)
CREB3L2	2.45	2.24E-03	9.63E-02	cAMP responsive element binding protein 3-like 2 (CREB3L2)
MTRR	2.63	2.24E-03	9.63E-02	5-methyltetrahydrofolate-homocysteine methyltransferase reductase (MTRR), transcript variant 2
ACTR3B	3.44	2.24E-03	9.63E-02	ARP3 actin-related protein 3 homolog B (yeast) (ACTR3B), transcript variant 2
HNRPLL	2.54	2.26E-03	9.67E-02	heterogeneous nuclear ribonucleoprotein L-like
TNFSF8	2.87	2.27E-03	9.69E-02	tumor necrosis factor (ligand) superfamily, member 8 (TNFSF8), transcript variant 1, mRNA
ATP2B1	3.16	2.30E-03	9.73E-02	ATPase, Ca++ transporting, plasma membrane 1 (ATP2B1), transcript variant 1
ENST00000379435	2.40	2.30E-03	9.73E-02	T cell receptor beta variable 20/OR9-2 (non-functional)
PRMT10	2.71	2.31E-03	9.73E-02	protein arginine methyltransferase 10 (putative) (PRMT10)
THRAP3	2.93	2.31E-03	9.73E-02	thyroid hormone receptor associated protein 3 (THRAP3)
A_33_P3397473	2.53	2.31E-03	9.73E-02	Unknown
BET1	3.41	2.31E-03	9.73E-02	blocked early in transport 1 homolog (S. cerevisiae) (BET1)
TES	3.15	2.33E-03	9.74E-02	testis derived transcript (3 LIM domains) (TES), transcript variant 2, mRNA
LPXN	1.97	2.34E-03	9.74E-02	leupaxin (LPXN), transcript variant 2
TNRC6C	2.70	2.35E-03	9.75E-02	trinucleotide repeat containing 6C (TNRC6C)
MBD1	3.27	2.36E-03	9.75E-02	methyl-CpG binding domain protein 1 (MBD1)
TOMM5	3.00	2.37E-03	9.78E-02	translocase of outer mitochondrial membrane 5 homolog (yeast) (TOMM5), nuclear gene encoding mitochondrial protein, transcript variant 3
SLC11A2	2.34	2.37E-03	9.78E-02	solute carrier family 11 (proton-coupled divalent metal ion transporters), member 2 (SLC11A2), transcript variant 4
SMARCA5	3.15	2.39E-03	9.78E-02	SWI/SNF related, matrix associated, actin dependent regulator of chromatin, subfamily a, member 5 (SMARCA5)
RBM38	3.34	2.39E-03	9.78E-02	RNA binding motif protein 38 (RBM38), transcript variant 1
ZBTB46	2.88	2.39E-03	9.78E-02	zinc finger and BTB domain containing 46 (ZBTB46)
RCOR1	2.78	2.40E-03	9.79E-02	REST corepressor 1 (RCOR1)

STAG2	1.88	2.40E-03	9.79E-02	stromal antigen 2 (STAG2), transcript variant 1
PREB	3.19	2.42E-03	9.81E-02	prolactin regulatory element binding (PREB)
GNAS	4.05	2.43E-03	9.82E-02	GNAS complex locus
ENST00000419160	2.76	2.43E-03	9.83E-02	Alu subfamily J sequence contamination warning entry, partial (13%)
TRAP1	3.17	2.45E-03	9.88E-02	TNF receptor-associated protein 1 (TRAP1)
NFATC3	3.32	2.46E-03	9.90E-02	nuclear factor of activated T-cells, cytoplasmic, calcineurin-dependent 3 (NFATC3), transcript variant 1

Table S4. Genes down-regulated in pSS salivary gland $CD3^+CD4^+CD45RA^-$ T cells

Gene Symbol	Log FC pSS/non-SS	p-value	Adj p-value	Description
lincRNA:chr3:65012510-65037885_R	0.44	2.81E-05	1.74E-02	lincRNA:chr3:65012510-65037885 reverse strand
AIF1	0.40	6.24E-05	2.23E-02	allograft inflammatory factor 1 (AIF1), transcript variant 2
CRNN	0.52	1.51E-04	3.17E-02	cornulin (CRNN)
VAMP2	0.48	1.65E-04	3.28E-02	vesicle-associated membrane protein 2 (synaptobrevin 2) (VAMP2)
lincRNA:chr18:48545077-48556150_F	0.47	2.23E-04	3.85E-02	lincRNA:chr18:48545077-48556150 forward strand
ZBP1	0.35	2.29E-04	3.85E-02	Z-DNA binding protein 1 (ZBP1), transcript variant 1
SLC5A10	0.48	2.34E-04	3.85E-02	solute carrier family 5 (sodium/glucose cotransporter), member 10 (SLC5A10), transcript variant 1
ADRB2	0.51	2.46E-04	3.93E-02	adrenergic, beta-2-, receptor, surface (ADRB2)
IL15	0.58	2.71E-04	4.14E-02	interleukin 15 (IL15), transcript variant 2
LOC256880	0.51	2.80E-04	4.22E-02	uncharacterized LOC256880 (LOC256880)
ENST00000439423	0.44	3.61E-04	4.77E-02	hypothetical LOC554174, mRNA (cDNA clone MGC:16743 IMAGE:4130428)
A_33_P3212570	0.44	3.62E-04	4.77E-02	Unknown
ERCC-00104_60	0.54	3.71E-04	4.81E-02	
lincRNA:chr8:128181722-128182314_R	0.38	4.20E-04	5.07E-02	lincRNA:chr8:128181722-128182314 reverse strand
ENST00000457108	0.44	4.12E-04	5.07E-02	Zinc finger protein 21 (Zinc finger protein KOX14)
lincRNA:chr6:22020628-22111150_R	0.45	4.17E-04	5.07E-02	lincRNA:chr6:22020628-22111150 reverse strand
C21orf67	0.42	4.43E-04	5.21E-02	chromosome 21 open reading frame 67 (C21orf67), transcript variant 1
SLC22A2	0.57	4.35E-04	5.21E-02	solute carrier family 22 (organic cation transporter), member 2 (SLC22A2)
BRD1	0.49	4.90E-04	5.36E-02	bromodomain containing 1 (BRD1)
BEST1	0.55	4.98E-04	5.39E-02	bestrophin 1 (BEST1), transcript variant 1
lincRNA:chr7:32969114-32982514_R	0.40	5.17E-04	5.50E-02	lincRNA:chr7:32969114-32982514 reverse strand
FASLG	0.33	5.92E-04	5.94E-02	Fas ligand (TNF superfamily, member 6) (FASLG)
ADH7	0.61	5.96E-04	5.94E-02	alcohol dehydrogenase 7 (class IV), mu or sigma polypeptide (ADH7), transcript variant 2
MKS1	0.44	6.20E-04	6.12E-02	Meckel syndrome, type 1 (MKS1), transcript variant 1
XAGE5	0.49	6.24E-04	6.14E-02	X antigen family, member 5 (XAGE5)
lincRNA:chr2:184032455-184084780_R	0.54	6.36E-04	6.22E-02	lincRNA:chr2:184032455-184084780 reverse strand
FKBP11	0.33	6.58E-04	6.26E-02	FK506 binding protein 11, 19 kDa (FKBP11), transcript variant 1
TPRN	0.49	6.75E-04	6.30E-02	taperin (TPRN)
PLD1	0.53	6.75E-04	6.30E-02	phospholipase D1, phosphatidylcholine-specific (PLD1), transcript variant 1
SLC4A10	0.42	6.96E-04	6.37E-02	solute carrier family 4, sodium bicarbonate transporter, member 10 (SLC4A10), transcript variant 2
lincRNA:chr10:50217119-50223094_F	0.49	7.15E-04	6.47E-02	lincRNA:chr10:50217119-50223094 forward strand
lincRNA:chr19:56791970-56826162_F	0.50	7.35E-04	6.52E-02	lincRNA:chr19:56791970-56826162 forward strand
C1orf88	0.58	7.34E-04	6.52E-02	chromosome 1 open reading frame 88 (C1orf88)
KLHL4	0.51	7.42E-04	6.53E-02	kelch-like 4 (Drosophila) (KLHL4), transcript variant 2
ATP13A2	0.42	7.81E-04	6.67E-02	ATPase type 13A2 (ATP13A2), transcript variant 1
C2orf53	0.50	7.80E-04	6.67E-02	chromosome 2 open reading frame 53 (C2orf53)
MMP1	0.48	7.99E-04	6.74E-02	matrix metallopeptidase 1 (interstitial collagenase) (MMP1), transcript variant 1
lincRNA:chr10:131848410-131919360_F	0.48	8.41E-04	6.93E-02	lincRNA:chr10:131848410-131919360 forward strand
LOC100652995	0.45	8.65E-04	6.99E-02	hypothetical LOC100652995 (LOC100652995)
ENST00000434150	0.47	8.75E-04	6.99E-02	Unknown
CLTB	0.55	8.75E-04	6.99E-02	clathrin, light chain B (CLTB), transcript variant 2
ENST00000414816	0.55	8.92E-04	6.99E-02	Homo sapiens hypothetical LOC400511 (FLJ45256)
ZFPM1	0.56	8.94E-04	6.99E-02	zinc finger protein, multitype 1 (ZFPM1)
CACNA1I	0.57	8.72E-04	6.99E-02	calcium channel, voltage-dependent, T type, alpha 1I subunit (CACNA1I), transcript variant 1
PHYHD1	0.56	9.05E-04	7.05E-02	phytanoyl-CoA dioxygenase domain containing 1 (PHYHD1), transcript variant 2
lincRNA:chr8:38616218-38630243_F	0.46	9.15E-04	7.09E-02	lincRNA:chr8:38616218-38630243 forward strand
GPC3	0.50	9.17E-04	7.09E-02	glypican 3 (GPC3), transcript variant 1

GZMB	0.52	9.22E-04	7.10E-02	granzyme B (granzyme 2, cytotoxic T-lymphocyte-associated serine esterase 1) (GZMB)
B3GALT6	0.55	9.34E-04	7.11E-02	UDP-Gal:betaGal beta 1,3-galactosyltransferase polypeptide 6 (B3GALT6)
ZNF202	0.39	9.77E-04	7.16E-02	zinc finger protein 202 (ZNF202)
TMEM104	0.48	9.64E-04	7.16E-02	transmembrane protein 104 (TMEM104)
lincRNA:chr5:93988844-94003469_F	0.52	9.83E-04	7.16E-02	lincRNA:chr5:93988844-94003469 forward strand
lincRNA:chr19:52195738-52206988_R	0.49	1.14E-03	7.50E-02	lincRNA:chr19:52195738-52206988 reverse strand
INTS5	0.54	1.15E-03	7.53E-02	integrator complex subunit 5 (INTS5),
ABCB1	0.30	1.19E-03	7.62E-02	ATP-binding cassette, sub-family B (MDR/TAP), member 1 (ABCB1)
lincRNA:chr16:88805249-88810399_F	0.57	1.20E-03	7.62E-02	lincRNA:chr16:88805249-88810399 forward strand
CCDC57	0.50	1.22E-03	7.70E-02	coiled-coil domain containing 57 (CCDC57)
FLAP	0.47	1.28E-03	7.78E-02	HSFLAP five-lipoxygenase activating protein (FLAP) {Homo sapiens} (exp=-1; wgp=0; cg=0), partial (66%)
KIR3DL1	0.50	1.35E-03	7.97E-02	killer cell immunoglobulin-like receptor, three domains, long cytoplasmic tail, 1 (KIR3DL1)
SLC27A1	0.46	1.36E-03	7.99E-02	solute carrier family 27 (fatty acid transporter), member 1 (SLC27A1)
AY203961	0.54	1.40E-03	8.15E-02	LP8151 mRNA, complete cds
ENST00000531225	0.50	1.41E-03	8.15E-02	Putative transmembrane protein C8orfK29
GGTLC2	0.51	1.41E-03	8.15E-02	gamma-glutamyltransferase light chain 2 (GGTLC2), transcript variant 1
ZNF285	0.45	1.42E-03	8.20E-02	zinc finger protein 285 (ZNF285)
MATK	0.47	1.45E-03	8.20E-02	megakaryocyte-associated tyrosine kinase (MATK), transcript variant 1
ZNF692	0.53	1.45E-03	8.20E-02	zinc finger protein 692
EDA	0.56	1.44E-03	8.20E-02	ectodysplasin A (EDA), transcript variant 3
A_33_P3260021	0.54	1.51E-03	8.38E-02	Unknown
PHOSPHO1	0.59	1.52E-03	8.40E-02	phosphatase, orphan 1 (PHOSPHO1), transcript variant 1
CU691877	0.59	1.58E-03	8.63E-02	Synthetic construct Homo sapiens gateway clone IMAGE:100021124 3' read C1QTNF9
PLEKHG3	0.48	1.59E-03	8.63E-02	pleckstrin homology domain containing, family G (with RhoGef domain) member 3 (PLEKHG3)
ADAM8	0.50	1.59E-03	8.63E-02	ADAM metallopeptidase domain 8 (ADAM8), transcript variant 1
ARX	0.58	1.60E-03	8.64E-02	aristaless related homeobox (ARX)
ENST00000381261	0.40	1.60E-03	8.64E-02	A kinase (PRKA) anchor protein 17A
lincRNA:chr4:93175777-93192627_F	0.56	1.61E-03	8.64E-02	lincRNA:chr4:93175777-93192627 forward strand
GOSR1	0.41	1.63E-03	8.67E-02	golgi SNAP receptor complex member 1 (GOSR1), transcript variant 3
MUC3A	0.47	1.63E-03	8.67E-02	mucin 3A, cell surface associated (MUC3A)
FFAR3	0.53	1.62E-03	8.67E-02	free fatty acid receptor 3 (FFAR3)
lincRNA:chr6:113682343-113682601_F	0.57	1.63E-03	8.67E-02	lincRNA:chr6:113682343-113682601 forward strand
ERCC-00108_60	0.61	1.66E-03	8.74E-02	
SLC2A11	0.66	1.69E-03	8.85E-02	solute carrier family 2 (facilitated glucose transporter), member 11
lincRNA:chr8:49499922-49506997_F	0.52	1.70E-03	8.86E-02	lincRNA:chr8:49499922-49506997 forward strand
LOC100132356	0.46	1.71E-03	8.88E-02	uncharacterized LOC100132356 (LOC100132356)
lincRNA:chr15:25322012-25328606_F	0.62	1.71E-03	8.89E-02	lincRNA:chr15:25322012-25328606 forward strand
LOC440297	0.49	1.74E-03	8.94E-02	chondroitin sulfate proteoglycan 4 pseudogene (LOC440297)
A_33_P3385671	0.61	1.77E-03	8.94E-02	Unknown
C14orf93	0.49	1.80E-03	8.95E-02	chromosome 14 open reading frame 93 (C14orf93), transcript variant 1
HMGA2	0.55	1.81E-03	8.96E-02	high mobility group AT-hook 2 (HMGA2), transcript variant 2
AK123110	0.58	1.90E-03	9.12E-02	cDNA FLJ41115 fis, clone BRACE1000533
RRM1	0.51	1.95E-03	9.21E-02	ribonucleotide reductase M1 (RRM1)
A_33_P3247068	0.52	1.96E-03	9.21E-02	Unknown
A_33_P3232637	0.55	1.97E-03	9.21E-02	Unknown
MSMB	0.56	1.99E-03	9.25E-02	microseminoprotein, beta- (MSMB)
CSF1	0.56	1.99E-03	9.26E-02	colony stimulating factor 1 (macrophage) (CSF1), transcript variant 1
A_33_P3268395	0.55	2.03E-03	9.30E-02	Unknown
SSTR3	0.58	2.03E-03	9.30E-02	somatostatin receptor 3 (SSTR3)

DMPK	0.47	2.06E-03	9.31E-02	dystrophia myotonica-protein kinase (DMPK)
GTPBP5	0.57	2.05E-03	9.31E-02	GTP binding protein 5 (putative) (GTPBP5)
ENST00000401851	0.64	2.06E-03	9.31E-02	hypothetical protein MGC10955
MUC7	0.43	2.12E-03	9.45E-02	mucin 7, secreted (MUC7), transcript variant 3
lincRNA:chr18:77398927-77439745_R	0.60	2.13E-03	9.46E-02	lincRNA:chr18:77398927-77439745 reverse strand
NEK6	0.54	2.15E-03	9.48E-02	NIMA (never in mitosis gene a)-related kinase 6 (NEK6), transcript variant 2
SUN5	0.56	2.15E-03	9.48E-02	Sad1 and UNC84 domain containing 5 (SUN5)
PNPO	0.49	2.19E-03	9.53E-02	pyridoxamine 5'-phosphate oxidase (PNPO)
CK906254	0.68	2.19E-03	9.53E-02	ie98c03.y5 Melton Normalized Human Islet 4 N4-HIS 1 Homo sapiens cDNA clone IMAGE:5674757 5'
CNTN1	0.55	2.21E-03	9.57E-02	contactin 1 (CNTN1), transcript variant 1
A_33_P3240652	0.44	2.24E-03	9.63E-02	Unknown
PNKD	0.61	2.26E-03	9.67E-02	paroxysmal nonkinesigenic dyskinesia (PNKD), nuclear gene encoding mitochondrial protein, transcript variant 1
PLCG2	0.65	2.34E-03	9.74E-02	phospholipase C, gamma 2 (phosphatidylinositol-specific) (PLCG2)
NAT8B	0.52	2.38E-03	9.78E-02	N-acetyltransferase 8B (GCN5-related, putative, gene/pseudogene) (NAT8B)
PLEKHF1	0.60	2.39E-03	9.78E-02	pleckstrin homology domain containing, family F (with FYVE domain) member 1 (PLEKHF1)
GJA4	0.60	2.39E-03	9.78E-02	gap junction protein, alpha 4, 37kDa (GJA4)
MCC	0.59	2.40E-03	9.79E-02	mutated in colorectal cancers (MCC), transcript variant 1
DSCAML1	0.65	2.41E-03	9.79E-02	Down syndrome cell adhesion molecule like 1 (DSCAML1)
LOC728684	0.50	2.42E-03	9.81E-02	cDNA FLJ44019 fis, clone TESTI4026192
ACOT11	0.52	2.46E-03	9.90E-02	acyl-CoA thioesterase 11 (ACOT11), transcript variant 2
ENST00000525262	0.62	2.48E-03	9.96E-02	Unknown

Table S5. Ingenuity Diseases and Functions enriched among DEGs in SG CD4+CD45RA- T cells of pSS cases versus nSS controls

Disease or Function	p-value	Z-score[a]	# DEGs in Category	Disease and Function Molecules Present in DEG Dataset
Lymphohematopoietic cancer	1.21E-09	-0.894	126	ABCB1,ACOT11,ADRB2,ADSL,AKAP17A,AKAP8,APAF1,APH1A,ARHGAP35,ARIH1,ATP6V1A,ATRN,BEST1,CBX7,CCR7,CD2,CD200,CEP68,CHD6,CLTB,CRNN,CSF1R,CTLA4,CXCL13,CXCR5,DHFR,DLEU1,DNMT3A,ELK1,ELMO1,EPSTI1,ETFA,EVC,FAM86B2/FAM86KP,FANCD2,FASLG,FCMR,FDPS,FILIP1L,FKBP3,FOXO1,GCC2,GJA4,GLS,GNA13,GNAQ,GNAS,GZMB,HMGA2,IFITM1,IGFBP4,IKBKE,IL15,ILKAP,IPO7,KIR3DL1,KLHL4,LIMS3/LIMS4,LONP1,LYPLA1,MAML2,MBD1,MCM6,MGA,MLF1,MMP1,MOCS1,MRPS5,NEDD9,NEK4,NEK6,NF1,NONO,NR3C1,OAS2,OSBP,PDE3B,PDE4DIP,PDPK1,PHF3,PHKB,PHYHD1,PIK3CD,PLAC8,PLCG2,PLEKHG2,PLEKHG3,PNKD,POLB,POLE,PPP2CA,PPP4R2,PRKCB,PRR14L,RAB4A,RASA4,RASSF5,RBCK1,RBM38,RICTOR,RRM1,S1PR1,SATB1,SELL,SETD1B,SIRPG,SKIV2L2,SKP2,SLC4A10,SSTR3,STAG2,STAT1,TCF7,TFRC,THRAP3,TIGIT,TLE3,TNFSF8,TNRC6C,UBE3A,UCKL1,UROS,USP3,VMP1,ZBTB24,ZNF827
Lymphoproliferative disorder	1.27E-09	↓ -2.183	89	ACOT11,ADRB2,ADSL,AKAP8,APAF1,APH1A,ARIH1,ATRN,CCR7,CD2,CD200,CEP68,CHD6,CRNN,CSF1R,CTLA4,CXCL13,CXCR5,DHFR,DLEU1,DNMT3A,ELK1,ELMO1,FANCD2,FASLG,FCMR,FDPS,FILIP1L,FKBP3,FOXO1,GJA4,GNA13,GNAQ,GZMB,HMGA2,IFITM1,IGFBP4,IKBKE,IL15,IPO7,KIR3DL1,KLHL4,LONP1,LYPLA1,MAML2,MBD1,MGA,MMP1,NEDD9,NEK4,NEK6,NF1,NONO,NR3C1,OAS2,OSBP,PDE3B,PIK3CD,PLAC8,PLCG2,PLEKHG3,PNKD,POLB,POLE,PPP2CA,PRKCB,RAB4A,RASSF5,RBCK1,RBM38,RICTOR,RRM1,S1PR1,SATB1,SELL,SKIV2L2,SKP2,SLC4A10,STAG2,STAT1,TCF7,TIGIT,TNFSF8,TNRC6C,UBE3A,UCKL1,UROS,VMP1,ZBTB24
Hematologic cancer	1.55E-09	-0.894	125	ABCB1,ACOT11,ADRB2,ADSL,AKAP17A,AKAP8,APAF1,APH1A,ARHGAP35,ARIH1,ATP6V1A,ATRN,BEST1,CBX7,CCR7,CD2,CD200,CEP68,CHD6,CLTB,CRNN,CSF1R,CTLA4,CXCL13,CXCR5,DHFR,DLEU1,DNMT3A,ELK1,ELMO1,EPSTI1,ETFA,EVC,FAM86B2/FAM86KP,FANCD2,FASLG,FCMR,FDPS,FILIP1L,FKBP3,FOXO1,GCC2,GJA4,GLS,GNA13,GNAQ,GNAS,GZMB,HMGA2,IFITM1,IGFBP4,IKBKE,IL15,ILKAP,IPO7,KIR3DL1,KLHL4,LIMS3/LIMS4,LONP1,LYPLA1,MAML2,MBD1,MCM6,MGA,MLF1,MMP1,MOCS1,MRPS5,NEDD9,NEK4,NEK6,NF1,NONO,NR3C1,OAS2,OSBP,PDE3B,PDE4DIP,PDPK1,PHF3,PHKB,PHYHD1,PIK3CD,PLAC8,PLCG2,PLEKHG2,PLEKHG3,PNKD,POLB,POLE,PPP2CA,PPP4R2,PRKCB,PRR14L,RAB4A,RASA4,RASSF5,RBCK1,RBM38,RICTOR,RRM1,S1PR1,SATB1,SELL,SETD1B,SIRPG,SKIV2L2,SKP2,SLC4A10,STAG2,STAT1,TCF7,TFRC,THRAP3,TIGIT,TLE3,TNFSF8,TNRC6C,UBE3A,UCKL1,UROS,USP3,VMP1,ZBTB24,ZNF827
Lymphoid cancer	4.24E-09	-0.902	122	ABCB1,ACOT11,ADRB2,ADSL,AKAP17A,AKAP8,APAF1,APH1A,ARHGAP35,ARIH1,ATP6V1A,ATRN,BEST1,CCR7,CD2,CD200,CEP68,CHD6,CLTB,CRNN,CSF1R,CTLA4,CXCL13,CXCR5,DHFR,DLEU1,DNMT3A,ELK1,ELMO1,ETFA,EVC,FAM86B2/FAM86KP,FANCD2,FASLG,FCMR,FDPS,FILIP1L,FKBP3,FOXO1,GCC2,GJA4,GLS,GNA13,GNAQ,GNAS,GZMB,HMGA2,IFITM1,IGFBP4,IKBKE,IL15,ILKAP,IPO7,KIR3DL1,KLHL4,LIMS3/LIMS4,LONP1,LYPLA1,MAML2,MBD1,MCM6,MGA,MLF1,MMP1,MOCS1,MRPS5,NEDD9,NEK4,NEK6,NF1,NONO,NR3C1,OAS2,OSBP,PDE3B,PDE4DIP,PDPK1,PHKB,PHYHD1,PIK3CD,PLAC8,PLCG2,PLEKHG2,PLEKHG3,PNKD,POLB,POLE,PPP2CA,PRKCB,PRR14L,RAB4A,RASA4,RASSF5,RBCK1,RBM38,RICTOR,RRM1,S1PR1,SATB1,SELL,SETD1B,SIRPG,SKIV2L2,SKP2,SLC4A10,SSTR3,STAG2,STAT1,TCF7,TFRC,THRAP3,TIGIT,TLE3,TNFSF8,TNRC6C,UBE3A,UCKL1,UROS,USP3,VMP1,ZBTB24,ZNF827
Necrosis	1.42E-08	-1.691	124	AAK1,ABCB1,ADAM8,ADI1,ADRB2,APAF1,ATP13A2,ATP2B1,ATP2C1,BIRC2,BRMS1,BUB3,CCR7,CD2,CD200,CKAP2,CLASP1,COPB2,CREB3L2,CSF1,CSF1R,CTLA4,CUL4B,DCTD,DHFR,DMPK,DNMT3A,DYNLL1,EEF2,EIF3G,ELK1,FANCD2,FASLG,FAU,FCMR,FCRL3,FDPS,FOXO1,GEM,GLS,GNA13,GNAQ,GNAS,GNL3,GPC3,GZMB,HDGF,HMGA2,HSPA4,IGFBP4,IGHG1,IKBKE,IL15,IL6ST,ILKAP,IRF9,KIR3DL1,LIMS1,LONP1,LYPLA1,MAML2,MBD1,MDN1,MMP1,MTG2,NAT10,NEDD9,NEK4,NEK6,NF1,NFKBID,NR3C1,PDE3B,PDPK1,PIK3CD,PLAC8,PLAT,PLCG2,PLD1,PLEKHF1,POLB,POLR3E,PPP1CC,PPP2CA,PRKAR1A,PRKCB,PSMC6,PSMD12,RAPGEF2,RASA4,RASSF5,RBCK1,RBL1,RBM8A,RICTOR,RNF19A,RPA2,RPL19,RRM1,S1PR1,SATB1,SELL,SF3B3,SKP2,SLC11A2,SLC22A2,SMN1/SMN2,SSRP1,SSTR3,ST6GAL1,STAT1,TCF7,TERF1,TFRC,TNFSF8,TPP1,TPP2,TRAP1,UBA7,UBE3A,VOPP1,WTAP,YME1L1,ZC3H8
Lymphoproliferative malignancy	2.46E-08	-1.459	83	ACOT11,ADRB2,ADSL,AKAP8,ATRN,CCR7,CD2,CD200,CEP68,CHD6,CRNN,CSF1R,CTLA4,CXCL13,CXCR5,DHFR,DLEU1,DNMT3A,ELK1,ELMO1,FASLG,FCMR,FDPS,FILIP1L,FKBP3,FOXO1,GJA4,GNA13,GZMB,HMGA2,IFITM1,IGFBP4,IKBKE,IL15,IPO7,KIR3DL1,KLHL4,LONP1,LYPLA1,MAML2,MBD1,MGA,MMP1,NEDD9,NEK4,NEK6,NF1,NONO,NR3C1,OAS2,OSBP,PDE3B,PIK3CD,PLAC8,PLCG2,PLEKHG3,PNKD,POLB,POLE,PPP2CA,PRKCB,RAB4A,RASSF5,RBCK1,RBM38,RICTOR,RRM1,S1PR1,SATB1,SELL,SKIV2L2,SKP2,SLC4A10,STAT1,TCF7,TIGIT,TNFSF8,TNRC6C,UBE3A,UCKL1,UROS,VMP1,ZBTB24
Lymphoma	3.30E-08	-1.179	67	ADRB2,ADSL,AKAP8,ATRN,CCR7,CD2,CD200,CHD6,CRNN,CSF1R,CTLA4,CXCL13,CXCR5,DHFR,DNMT3A,ELK1,FASLG,FDPS,FILIP1L,FKBP3,FOXO1,GJA4,GNA13,GZMB,HMGA2,IFITM1,IGFBP4,IKBKE,IL15,IPO7,KIR3DL1,LONP1,MBD1,MGA,MMP1,NEK4,NEK6,NF1,NONO,NR3C1,OAS2,OSBP,PIK3CD,PLAC8,PLCG2,PLEKHG3,POLB,POLE,PPP2CA,PRKCB,RAB4A,RASSF5,RBCK1,RBM38,RICTOR,RRM1,SATB1,SELL,SKIV2L2,SKP2,SLC4A10,STAT1,TNFSF8,TNRC6C,UBE3A,UROS,ZBTB24
Lymphocytic neoplasm	3.81E-08	-1.459	84	ACOT11,ADRB2,ADSL,AKAP8,ATRN,CCR7,CD2,CD200,CEP68,CHD6,CRNN,CSF1R,CTLA4,CXCL13,CXCR5,DHFR,DLEU1,DNMT3A,ELK1,ELMO1,FASLG,FCMR,FDPS,FILIP1L,FKBP3,FOXO1,GJA4,GNA13,GZMB,HMGA2,IFITM1,IGFBP4,IKBKE,IL15,IPO7,KIR3DL1,KLHL4,LONP1,LYPLA1,MAML2,MBD1,MGA,MMP1,NEDD9,NEK4,NEK6,NF1,NONO,NR3C1,OAS2,OSBP,PDE3B,PIK3CD,PLAC8,PLCG2,PLEKHG3,PNKD,POLB,POLE,PPP2CA,PRKCB,RAB4A,RASSF5,RBCK1,RBM38,RICTOR,RRM1,S1PR1,SATB1,SELL,SKIV2L2,SKP2,SLC4A10,STAT1,TCF7,TFRC,TIGIT,TNFSF8,TNRC6C,UBE3A,UCKL1,UROS,VMP1,ZBTB24

Non-Hodgkin disease	8.60E-08		58	ADSL,AKAP8,ATRN,CCR7,CD2,CD200,CHD6,CRNN,CSF1R,CXCL13,CXCR5,DHFR,DNMT3A,ELK1,FASLG,FDPS,FILIP1L,FOXO1,GJA4,GNA13,GZMB,IFITM1,IGFBP4,IKBKE,IL15,KIR3DL1,LONP1,MBD1,MGA,MMP1,NEK4,NEK6,NF1,NONO,NR3C1,OAS2,OSBP,PIK3CD,PLCG2,PLEKHG3,POLB,POLE,PPP2CA,PRKCB,RAB4A,RBCK1,RICTOR,RRM1,SATB1,SELL,SKIV2L2,SKP2,SLC4A10,STAT1,TNRC6C,UBE3A,UROS,ZBTB24
Cell death	1.47E-07	↓ -2.118	145	AAK1,ABCB1,ADAM8,ADI1,ADRB2,AGO2,AIF1,AKAP8,APAF1,APH1A,ARHGAP35,ARX,ATP13A2,ATP2B1,ATP2C1,BIRC2,BRD1,BRMS1,BUB3,CBX7,CCR7,CD2,CD200,CKAP2,CLASP1,COPB2,CREB3L2,CSF1,CSF1R,CTLA4,CUL4B,DCTD,DDX42,DHFR,DMPK,DNMT3A,DYNLL1,EDA,EEF2,EIF3G,ELK1,ELMO1,FANCD2,FASLG,FAU,FCMR,FCRL3,FDPS,FFAR3,FOXO1,GEM,GLS,GNA13,GNAQ,GNAS,GNL3,GPC3,GZMB,HDGF,HMGA2,HSPA4,IGFBP4,IGHG1,IKBKE,IL15,IL6ST,ILKAP,IRF9,KIR3DL1,LIMS1,LONP1,LYPLA1,MAML2,MBD1,MCFD2,MDN1,MMP1,MTG2,NAT10,NAT8B,NEDD9,NEK4,NEK6,NF1,NFKBID,NOD2,NR3C1,PDE3B,PDPK1,PIK3CD,PLAC8,PLAT,PLCG2,PLD1,PLEKHF1,POLB,POLR3E,PPP1CC,PPP2CA,PRKAR1A,PRKCB,PSMC6,PSMD12,RAPGEF2,RASA4,RASSF2,RASSF5,RBCK1,RBL1,RBM8A,RICTOR,RNF19A,RPA2,RPL19,RRM1,S1PR1,SATB1,SELENON,SELL,SF3B3,SKP2,SLC11A2,SLC22A2,SMARCA5,SMN1/SMN2,SSRP1,SSTR3,ST6GAL1,STAT1,TAB2,TCF7,TERF1,TFRC,TNFSF8,TPP1,TPP2,TRAP1,TXK,UBA7,UBE3A,VAMP2,VOPP1,WTAP,YME1L1,ZC3H8
Quantity of blood cells	1.57E-07	0.537	59	ABCB1,ADAM8,BIRC2,BRD1,CCR7,CD200,CD302,CSF1,CSF1R,CTLA4,CXCL13,CXCR5,DAPP1,DKC1,DNMT3A,ELMO1,FANCD2,FASLG,FCMR,FOXO1,GLS,GNA13,GNAS,GPC3,HMGA2,IL15,IL6ST,MATK,NEDD9,NF1,NFATC3,NLRC5,NOD2,NR3C1,PIK3CD,PILRA,PLAC8,PLAT,PLCG2,POLR3E,PPP2CA,PRKCB,RAPGEF2,RASSF2,RASSF5,RBM38,RCOR1,RICTOR,S1PR1,SATB1,SELL,SLC11A2,ST6GAL1,STAT1,TFRC,TIGIT,TPP2,TXK,ZBTB46
Myelomonocytic leukemia	3.76E-07		13	ABCB1,APAF1,APH1A,ARIH1,CSF1R,DHFR,DNMT3A,FANCD2,NF1,NR3C1,POLB,RRM1,STAG2
Trafficking of lymphocytes	4.97E-07	1.941	8	CCR7,CXCL13,FOXO1,IL15,NEDD9,PDPK1,S1PR1,SELL
Systemic lupus erythematosus	6.56E-07		19	ABCB1,AGFG1,CTLA4,DHFR,FASLG,FOXO1,GNAQ,GZMB,IL6ST,NR3C1,PPP2CA,S1PR1,SELL,SKIV2L,ST6GAL1,STAT1,TCF7,TLE3,ZBP1
Chronic myelomonocytic leukemia	1.67E-06		12	APAF1,APH1A,ARIH1,CSF1R,DHFR,DNMT3A,FANCD2,NF1,NR3C1,POLB,RRM1,STAG2
Apoptosis	2.64E-06	-1.357	114	ABCB1,ADAM8,ADI1,ADRB2,AGO2,AIF1,AKAP8,APAF1,APH1A,ARHGAP35,ATP2C1,BIRC2,BRD1,BRMS1,BUB3,CCR7,CD2,CD200,CKAP2,CLASP1,CREB3L2,CSF1,CSF1R,CTLA4,CUL4B,DDX42,DHFR,DMPK,DNMT3A,DYNLL1,EDA,ELK1,ELMO1,FASLG,FAU,FCMR,FFAR3,FOXO1,GEM,GLS,GNA13,GNAQ,GNAS,GNL3,GPC3,GZMB,HDGF,HMGA2,HSPA4,IGFBP4,IGHG1,IKBKE,IL15,IL6ST,ILKAP,KIR3DL1,LIMS1,LYPLA1,MDN1,MMP1,NAT10,NAT8B,NEDD9,NEK6,NF1,NFKBID,NOD2,NR3C1,PDPK1,PIK3CD,PLAC8,PLAT,PLCG2,PLD1,PLEKHF1,POLB,POLR3E,PPP1CC,PPP2CA,PRKAR1A,PRKCB,RAPGEF2,RASA4,RASSF2,RASSF5,RBCK1,RBL1,RICTOR,RPA2,RRM1,S1PR1,SATB1,SELENON,SELL,SKP2,SLC22A2,SMARCA5,SMN1/SMN2,SSRP1,SSTR3,ST6GAL1,STAT1,TAB2,TERF1,TFRC,TNFSF8,TPP2,TRAP1,TXK,UBA7,VOPP1,WTAP,YME1L1,ZC3H8
Homing of lymphocytes	3.81E-06	↑ 2.252	15	ADAM8,CCR7,CXCL13,CXCR5,ELMO1,FOXO1,GNA13,GNAS,IGHG1,IL15,NEDD9,NR3C1,PIK3CD,S1PR1,SELL
Myelodysplastic-myeloproliferative disease	6.83E-06		13	ABCB1,APAF1,APH1A,ARIH1,CSF1R,DHFR,DNMT3A,FANCD2,NF1,NR3C1,POLB,RRM1,STAG2
Chronic leukemia	8.63E-06		33	AKAP8,APAF1,APH1A,ARIH1,CD200,CEP68,CSF1R,DHFR,DLEU1,DNMT3A,FANCD2,FASLG,FCMR,FOXO1,GNAQ,LONP1,LYPLA1,NEDD9,NF1,NR3C1,PDE3B,PIK3CD,PLCG2,PNKD,POLB,POLE,PRKCB,RASSF5,RRM1,S1PR1,STAG2,TIGIT,UCKL1
Chronic inflammation of joint	9.14E-06		3	IL6ST,PLAT,STAT1
Cell viability of leukocytes	1.14E-05	1.418	20	CCR7,CSF1,CSF1R,CTLA4,FASLG,FCMR,FCRL3,FOXO1,IL15,IL6ST,NF1,PDPK1,PIK3CD,PLAT,PLCG2,PRKCB,RICTOR,STAT1,TCF7,TNFSF8
Quantity of leukocytes	1.18E-05	0.296	49	ABCB1,ADAM8,BIRC2,CCR7,CD200,CD302,CSF1,CSF1R,CTLA4,CXCL13,CXCR5,DAPP1,DKC1,DNMT3A,ELMO1,FASLG,FCMR,FOXO1,GLS,GNA13,GNAS,IL15,IL6ST,NEDD9,NF1,NFATC3,NLRC5,NOD2,NR3C1,PIK3CD,PILRA,PLAC8,PLAT,PLCG2,POLR3E,PPP2CA,PRKCB,RASSF2,RASSF5,RICTOR,S1PR1,SATB1,SELL,ST6GAL1,STAT1,TIGIT,TPP2,TXK,ZBTB46
Diffuse small-cell lymphoma	1.32E-05		26	ADSL,CHD6,CRNN,CSF1R,DHFR,ELK1,FILIP1L,GNA13,IKBKE,LONP1,MBD1,MGA,NEK4,NR3C1,OSBP,PIK3CD,PLCG2,PLEKHG3,POLB,POLE,RBCK1,RRM1,SKIV2L2,SLC4A10,UBE3A,UROS
Morphology of lymph node	1.35E-05		15	CCR7,CD200,CTLA4,CXCR5,FASLG,IGHG1,IL15,NFATC3,NOD2,PIK3CD,RASSF5,SATB1,SELL,TPP2,ZBTB46
Activation of B lymphocytes	1.36E-05	0.788	13	CCR7,CXCR5,DAPP1,FCRL3,IGHG1,IL15,NOD2,PIK3CD,PLCG2,PRKCB,ST6GAL1,TAB2,TAB3
Chronic myeloid leukemia	1.44E-05		15	APAF1,APH1A,ARIH1,CSF1R,DHFR,DNMT3A,FANCD2,FASLG,FOXO1,GNAQ,NF1,NR3C1,POLB,RRM1,STAG2
Homing of mononuclear leukocytes	1.65E-05	↑ 2.396	17	ADAM8,AIF1,CCR7,CXCL13,CXCR5,ELMO1,FOXO1,GNA13,GNAS,IGHG1,IL15,NEDD9,NR3C1,PIK3CD,PRKCB,S1PR1,SELL
Type M4 acute myeloid leukemia	1.66E-05		6	ABCB1,CSF1R,DHFR,NR3C1,POLB,RRM1
Immune response of cells	1.78E-05	0.656	34	ABCB1,BIRC2,CCR7,CD200,CD302,CHN1,CSF1,CTLA4,EIF4EBP2,ELMO1,FASLG,FCMR,FOXO1,GNA13,GNAS,HSDL1,IGHG1,IL15,IL6ST,NOD2,NR3C1,PLD1,PRKCB,RAB43,RASA4,RICTOR,SLC22A2,SNX3,SPRY1,STAT1,TIGIT,TNFSF8,TRIM14,TRIM5
Psoriatic arthritis	1.88E-05		10	APAF1,DHFR,FOXO1,IGHG1,NOD2,NR3C1,POLB,PPFIA1,PRKCB,TCF7
Abnormal morphology of lymph node	2.13E-05		14	CCR7,CD200,CTLA4,CXCR5,FASLG,IL15,NFATC3,NOD2,PIK3CD,RASSF5,SATB1,SELL,TPP2,ZBTB46
Leukemia	2.19E-05	-0.588	78	ABCB1,ACOT11,ADRB2,AKAP17A,AKAP8,APAF1,APH1A,ARHGAP35,ARIH1,ATP6V1A,BEST1,CCR7,CD2,CD200,CEP68,CLTB,CSF1R,CXCL13,DHFR,DLEU1,DNMT3A,ELMO1,ETFA,EVC,FAM86B2/FAM86KP,FANCD2,FASLG,FCMR,FOXO1,GCC2,GLS,GNAQ,GNAS,GZMB,IL15,ILKAP,IPO7,KLHL4,LIMS3/LIMS4,LONP1,LYPLA1,MAML2,MCM6,MLF1,MOCS1,MRPS5,NEDD9,NF1,NR3C1,PDE3B,PDE4DIP,PDPK1,PHF3,PHKB,PHYHD1,PIK3CD,PLCG2,PNKD,POLB,POLE,PRKCB,PRR14L,RASA4,RASSF5,RRM1,S1PR1,SELL,SETD1B,SIRPG,SKP2,STAG2,TCF7,THRAP3,TIGIT,UCKL1,USP3,VMP1,ZNF827

Transcription	2.28E-05	1.075	90	ADRB2,AGO2,AIF1,ARHGAP35,ARX,ATF7IP,ATP2C1,BIRC2,BRMS1,BRWD1,CASK,CBX7,CKAP2,CKS1B,CNTN1,CREB3L2,CSF1,DNMT3A,EIF4A3,ELK1,ELMO1,FASLG,FOXO1,GCFC2,GEM,GNA13,GNAQ,GNAS,GTF3C2,HDGF,HMGA2,HSPA4,IKBKE,IL6ST,IRF9,KDM7A,LIMS1,MAML2,MBD1,METTL23,MGA,MLF1,NAB1,NAT8B,NDFIP2,NEK4,NEK6,NFATC3,NFKBID,NLRC5,NOD2,NONO,NR3C1,PARP9,PIK3CD,PLAC8,PLEKHG2,PPP1CC,PPP2CA,PREB,PRKAR1A,PRKCB,PVT1,RAB3GAP1,RBAK,RBCK1,RBL1,RCOR1,RPAIN,S1PR1,SATB1,SMARCA5,SNIP1,SSRP1,STAT1,TAB2,TAF7,TCF7,THRAP3,TLE3,TNFSF8,TXK,UBE3A,UBN1,USP3,ZBP1,ZC3H8,ZFPM1,ZNF202,ZNF692
Mycosis fungoides	2.36E-05		9	CCR7,CD2,CXCL13,DHFR,IL15,NR3C1,POLE,RRM1,SELL
Quantity of lymphoid tissue	2.55E-05	1.863	25	ABCB1,CCR7,CD200,CTLA4,CXCL13,CXCR5,FCMR,FOXO1,GNA13,IL6ST,NEDD9,NFATC3,NOD2,NR3C1,PIK3CD,PLCG2,POLR3E,RASSF5,RBM38,RICTOR,SATB1,ST6GAL1,STAT1,TPP2,TXK
Cell death of tumor cell lines	2.59E-05	-1.165	73	ABCB1,ADI1,APAF1,ATP13A2,ATP2B1,BIRC2,BUB3,CKAP2,CLASP1,CREB3L2,CSF1R,DCTD,DHFR,DNMT3A,DYNLL1,EEF2,EIF3G,ELK1,FASLG,FAU,FCMR,FOXO1,GEM,GLS,GNA13,GNAS,GPC3,GZMB,HSPA4,IGFBP4,IGHG1,IKBKE,IL15,IL6ST,ILKAP,KIR3DL1,LIMS1,LONP1,LYPLA1,MAML2,MBD1,MTG2,NAT10,NEDD9,NEK6,NF1,NR3C1,PDPK1,PIK3CD,PLAT,PLCG2,PPP2CA,PRKAR1A,PRKCB,RAPGEF2,RASSF5,RICTOR,RPA2,RRM1,SATB1,SF3B3,SKP2,SLC11A2,SSRP1,ST6GAL1,STAT1,TERF1,TFRC,TPP2,TRAP1,UBA7,UBE3A,VOPP1
Morphology of blood cells	2.71E-05		27	ABCB1,CD200,CSF1,CSF1R,CTLA4,CXCR5,FANCD2,FASLG,FCMR,GNA13,GNAQ,IGHG1,IL15,NOD2,PDPK1,PIK3CD,PLCG2,RAPGEF2,RASSF2,RBL1,RBM38,SLC11A2,STAT1,TFRC,TPP2,ZBTB46,ZFPM1
Quantity of lymphatic system cells	2.82E-05	1.636	42	ABCB1,BIRC2,CCR7,CSF1,CSF1R,CTLA4,CXCL13,CXCR5,DAPP1,DKC1,DNMT3A,FASLG,FCMR,FOXO1,GLS,GNA13,IL15,IL6ST,NEDD9,NF1,NFATC3,NLRC5,NOD2,NR3C1,PIK3CD,PLCG2,POLR3E,PRKCB,RAPGEF2,RASSF2,RASSF5,RBL1,RBM38,RICTOR,S1PR1,SATB1,SELL,ST6GAL1,STAT1,TIGIT,TPP2,TXK
Interaction of mononuclear leukocytes	2.90E-05	↑ 2.541	17	ATRN,CCR7,CD2,CTLA4,CXCL13,FASLG,FOXO1,IL15,IL6ST,NEDD9,NFATC3,NR3C1,RASSF5,RICTOR,SELL,TFRC,TXK
Trafficking of cells	2.94E-05	↑ 2.153	9	CCR7,CXCL13,CXCR5,FOXO1,IL15,NEDD9,PDPK1,S1PR1,SELL
Adult leukemia	3.14E-05		13	ABCB1,CCR7,CD2,CSF1R,DHFR,DNMT3A,NR3C1,POLB,POLE,PRKCB,RRM1,S1PR1,VMP1
Liquid tumor	3.53E-05	-0.636	82	ABCB1,ACOT11,ADRB2,AKAP17A,AKAP8,APAF1,APH1A,ARHGAP35,ARIH1,ATP6V1A,BEST1,CCR7,CD2,CD200,CEP68,CLTB,CSF1R,CXCL13,DHFR,DLEU1,DNMT3A,ELMO1,ETFA,EVC,FAM86B2/FAM86KP,FANCD2,FASLG,FCMR,FOXO1,GCC2,GLS,GNAQ,GNAS,GZMB,HMGA2,IL15,ILKAP,IPO7,KLHL4,LIMS3/LIMS4,LONP1,LYPLA1,MAML2,MCM6,MGA,MLF1,MMP1,MOCS1,MRPS5,NEDD9,NF1,NR3C1,PDE3B,PDE4DIP,PDPK1,PHF3,PHKB,PHYHD1,PIK3CD,PLCG2,PNKD,POLB,POLE,PRKCB,PRR14L,RASA4,RASSF5,RRM1,S1PR1,SELL,SETD1B,SIRPG,SKP2,STAG2,TCF7,THRAP3,TIGIT,TLE3,UCKL1,USP3,VMP1,ZNF827
Migration of marginal-zone B lymphocytes	3.61E-05	0.849	4	CXCL13,CXCR5,GNA13,S1PR1
Deletion of cells	3.85E-05		6	CD2,CTLA4,FASLG,IL15,IL6ST,TCF7
Rheumatic Disease	3.92E-05	-1.242	58	ABCB1,ADAM8,ADRB2,AGFG1,AIF1,APAF1,ARIH1,ATP2B1,ATP2C1,BRMS1,CCR7,CD200,CSF1,CSF1R,CTLA4,CXCL13,CXCR5,DHFR,DYNLL1,EEF2,EVC,FASLG,FAU,FCRL3,FDPS,FOXO1,GNAQ,GZMB,IGFBP4,IGHG1,IL15,IL6ST,KIR3DL1,MBD1,MMP1,NOD2,NONO,NR3C1,PDE3B,PIK3CD,PILRA,PLAT,PLCG2,POLB,PPFIA1,PPP2CA,PRKCB,RBM38,RPL19,S1PR1,SELL,SKIV2L,ST6GAL1,STAT1,TCF7,TFRC,TLE3,ZBP1
Binding of mononuclear leukocytes	4.06E-05	↑ 2.335	16	CCR7,CD2,CTLA4,CXCL13,FASLG,FOXO1,IL15,IL6ST,NEDD9,NFATC3,NR3C1,RASSF5,RICTOR,SELL,TFRC,TXK
Cell proliferation of tumor cell lines	4.39E-05	↑ 2.075	74	ABCB1,ADSL,APAF1,ARIH1,BIRC2,BUB3,CBX7,CKS1B,COPB2,CSF1,CSNK1G3,CTLA4,ELK1,FASLG,FOXO1,GJA4,GLS,GNAQ,GNAS,GNL3,GPC3,GZMB,HDGF,HMGA2,IGFBP4,IKBKE,IL15,IL6ST,ILKAP,IPO7,MAT2B,MATK,MMP1,MSMB,NAT10,NEDD9,NF1,NR3C1,PDPK1,PIK3CD,PLAC8,PLAT,PLCG2,PLD1,PPP1CC,PPP2CA,PRKAR1A,PRKCB,RAB30,RAPGEF2,RASSF5,RBCK1,RBL1,RBM38,RICTOR,RRM1,SATB1,SKP2,SMN1/SMN2,SNIP1,STAG2,STAT1,TAB2,TAB3,TAF7,TERF1,TES,TFRC,TNFSF8,TPP2,VAMP2,VBP1,VMP1,WTAP
Adult T cell leukemia	4.56E-05		12	CCR7,CD2,CSF1R,DHFR,DNMT3A,NR3C1,POLB,POLE,PRKCB,RRM1,S1PR1,VMP1
Quantity of lymphocytes	4.57E-05	1.739	39	ABCB1,BIRC2,CCR7,CSF1,CSF1R,CTLA4,CXCL13,CXCR5,DAPP1,DKC1,DNMT3A,FASLG,FCMR,FOXO1,GLS,GNA13,IL15,IL6ST,NEDD9,NF1,NFATC3,NLRC5,NOD2,NR3C1,PIK3CD,PLCG2,POLR3E,PRKCB,RASSF2,RASSF5,RICTOR,S1PR1,SATB1,SELL,ST6GAL1,STAT1,TIGIT,TPP2,TXK
Leukemia of lymphocytes	4.57E-05		18	CCR7,CD2,CSF1R,CXCL13,DHFR,DNMT3A,GZMB,IL15,NF1,NR3C1,POLB,POLE,PRKCB,RRM1,S1PR1,SELL,TCF7,VMP1
Cell death of immune cells	4.66E-05	0.062	36	ADAM8,APAF1,BIRC2,CCR7,CD2,CSF1,CSF1R,CTLA4,FASLG,FCMR,FCRL3,FOXO1,GLS,GNAS,GZMB,IKBKE,IL15,IL6ST,NF1,NFKBID,NR3C1,PDPK1,PIK3CD,PLAT,PLCG2,POLR3E,PRKCB,RICTOR,S1PR1,SATB1,ST6GAL1,STAT1,TCF7,TNFSF8,TPP2,ZC3H8
Chronic myeloproliferative neoplasm	4.78E-05		18	APAF1,APH1A,ARIH1,CSF1R,DHFR,DNMT3A,FANCD2,FASLG,FOXO1,GNAQ,HMGA2,IL15,NF1,NR3C1,POLB,POLE,RRM1,STAG2
Interaction of lymphocytes	4.81E-05	↑ 2.888	15	ATRN,CCR7,CD2,CTLA4,CXCL13,FASLG,IL6ST,NEDD9,NFATC3,NR3C1,RASSF5,RICTOR,SELL,TFRC,TXK
Lymphocytic leukemia	4.92E-05		38	ACOT11,AKAP8,CCR7,CD2,CD200,CEP68,CSF1R,CXCL13,DHFR,DLEU1,DNMT3A,ELMO1,FASLG,FCMR,GZMB,IL15,KLHL4,LONP1,LYPLA1,MAML2,NEDD9,NF1,NR3C1,PDE3B,PIK3CD,PLCG2,PNKD,POLB,POLE,PRKCB,RASSF5,RRM1,S1PR1,SELL,TCF7,TIGIT,UCKL1,VMP1
Quantity of mononuclear leukocytes	5.06E-05	1.333	40	ABCB1,BIRC2,CCR7,CSF1,CSF1R,CTLA4,CXCL13,CXCR5,DAPP1,DKC1,DNMT3A,FASLG,FCMR,FOXO1,GLS,GNA13,GNAS,IL15,IL6ST,NEDD9,NF1,NFATC3,NLRC5,NOD2,NR3C1,PIK3CD,PLCG2,POLR3E,PRKCB,RASSF2,RASSF5,RICTOR,S1PR1,SATB1,SELL,ST6GAL1,STAT1,TIGIT,TPP2,TXK

Quantity of B lymphocytes	5.19E-05	0.868	23	CCR7,CSF1,CXCL13,CXCR5,DAPP1,DKC1,FASLG,FCMR,FOXO1,GLS,GNA13,IL6ST,NEDD9,NFATC3,PIK3CD,PLCG2,PRKCB,RASSF2,RASSF5,S1PR1,ST6GAL1,STAT1,TPP2
Function of blood cells	5.20E-05	-1.076	31	ABCB1,ADRB2,CCR7,CD200,CSF1,CSF1R,CTLA4,FCMR,GNAQ,GPC3,IL15,NEDD9,NF1,NFKBID,NLRC5,NOD2,PIK3CD,PLAT,PLCG2,PLD1,RAPGEF2,RASSF5,S1PR1,SELL,SKP2,ST6GAL1,STAT1,TCF7,TIGIT,TPP2,TXK
Cell survival	5.21E-05	↑ 3.290	67	ABCB1,AGO2,AGPS,APAF1,ATP13A2,ATP2C1,BIRC2,CASK,CCR7,CD200,CKS1B,COPB2,CSF1,CSF1R,CTLA4,EDA,EEF2,EIF4A3,ELK1,FANCD2,FASLG,FCMR,FCRL3,FOXO1,GNA13,GPC3,GZMB,HDGF,HMGA2,HSPA4,IKBKE,IL15,IL6ST,IRF9,LIMS1,MCFD2,MMP1,NEDD9,NEK4,NF1,NR3C1,PDPK1,PIK3CD,PLAC8,PLAT,PLCG2,PLEKHG2,POLB,PPP1CC,PPP2CA,PRKAR1A,PRKCB,RICTOR,RRM1,S1PR1,SELL,SHPRH,SLC11A2,SLC22A2,SMN1/SMN2,SPRY1,STAT1,TCF7,TERF1,TNFSF8,VBP1,WTAP
Differentiation of hematopoietic progenitor cells	5.46E-05	0.408	22	APAF1,BRD1,CD2,CSF1,CSF1R,EEF2,ELK1,HMGA2,IL15,IL6ST,LONP1,MLF1,PIK3CD,PLCG2,PPP4R2,RCOR1,SATB1,SMARCA5,TCAF2,TFRC,ZBTB24,ZFPM1
Binding of lymphocytes	5.90E-05	↑ 2.697	14	CCR7,CD2,CTLA4,CXCL13,FASLG,IL6ST,NEDD9,NFATC3,NR3C1,RASSF5,RICTOR,SELL,TFRC,TXK
Apoptosis of mononuclear leukocytes	5.96E-05	0.085	23	ADAM8,CD2,CSF1,CTLA4,FASLG,FCMR,GNAS,GZMB,IKBKE,IL15,IL6ST,NFKBID,NR3C1,PDPK1,PIK3CD,PLCG2,POLR3E,PRKCB,S1PR1,SATB1,STAT1,TPP2,ZC3H8
Apoptosis of fibroblast cell lines	6.30E-05	-0.686	22	ABCB1,APAF1,BIRC2,CD2,CSF1R,FASLG,GLS,GNA13,GNAQ,GZMB,MDN1,NR3C1,PLAC8,PLEKHF1,PRKAR1A,PRKCB,RASA4,RASSF5,RBL1,SKP2,STAT1,YME1L1
FLT3 activating mutation positive acute myeloid leukemia	6.67E-05		6	CSF1R,DNMT3A,NR3C1,POLB,POLE,RRM1
Apoptosis of leukocytes	6.86E-05	-0.658	27	ADAM8,BIRC2,CCR7,CD2,CSF1,CTLA4,FASLG,FCMR,GNAS,GZMB,IKBKE,IL15,IL6ST,NFKBID,NR3C1,PDPK1,PIK3CD,PLAT,PLCG2,POLR3E,PRKCB,S1PR1,SATB1,ST6GAL1,STAT1,TPP2,ZC3H8
Activation of mononuclear leukocytes	6.94E-05	-0.412	28	CCR7,CD2,CD200,CSF1,CTLA4,CXCR5,DAPP1,FASLG,FCRL3,FOXO1,GJA4,HSPA4,IGHG1,IL15,KIR3DL1,NFATC3,NFKBID,NOD2,PIK3CD,PLCG2,PRKCB,SATB1,SIRPG,ST6GAL1,STAT1,TAB2,TAB3,TIGIT
Activation of lymphocytes	7.07E-05	-0.159	27	CCR7,CD2,CD200,CTLA4,CXCR5,DAPP1,FASLG,FCRL3,FOXO1,GJA4,HSPA4,IGHG1,IL15,KIR3DL1,NFATC3,NFKBID,NOD2,PIK3CD,PLCG2,PRKCB,SATB1,SIRPG,ST6GAL1,STAT1,TAB2,TAB3,TIGIT
B-cell lymphoproliferative disorder	7.16E-05		39	AKAP8,ATRN,CCR7,CHD6,CRNN,CSF1R,CXCL13,CXCR5,DHFR,ELK1,FASLG,FILIP1L,FOXO1,GNA13,LONP1,LYPLA1,MBD1,MGA,NEK4,NF1,NONO,NR3C1,OSBP,PIK3CD,PLCG2,PLEKHG3,POLB,POLE,PPP2CA,PRKCB,RAB4A,RBCK1,RICTOR,RRM1,S1PR1,SATB1,SKIV2L2,SLC4A10,TNRC6C
Expression of RNA	7.46E-05	0.638	92	ADRB2,AGO2,AIF1,ARHGAP35,ARX,ATF7IP,ATP2C1,BIRC2,BRMS1,BRWD1,CASK,CBX7,CKAP2,CKS1B,CNTN1,CREB3L2,CSF1,DHFR,DNMT3A,EIF3G,EIF4A3,EIF4EBP2,ELK1,FASLG,FOXO1,GCFC2,GEM,GNA13,GNAQ,GNAS,GTF3C2,HDGF,HMGA2,HSPA4,IKBKE,IL6ST,IRF9,KDM7A,LIMS1,MAML2,MBD1,METTL23,MGA,MLF1,NAB1,NAT8B,NDFIP2,NEK4,NEK6,NFATC3,NFKBID,NLRC5,NOD2,NONO,NR3C1,PARP9,PIK3CD,PLAC8,PLEKHG2,PPP1CC,PPP2CA,PREB,PRKAR1A,PRKCB,RAB3GAP1,RBAK,RBCK1,RBL1,RCOR1,RPAIN,RPL19,S1PR1,SATB1,SMARCA5,SNIP1,SSRP1,STAT1,TAB2,TAF7,TCF7,THRAP3,TLE3,TNFSF8,TXK,UBE3A,UBN1,USP3,ZBP1,ZC3H8,ZFPM1,ZNF202,ZNF692
Transcription of RNA	7.81E-05	1.285	81	ADRB2,AGO2,ARHGAP35,ARX,ATF7IP,ATP2C1,BIRC2,BRMS1,BRWD1,CASK,CBX7,CKAP2,CKS1B,CREB3L2,CSF1,DNMT3A,EIF4A3,ELK1,FASLG,FOXO1,GCFC2,GEM,GNA13,GNAQ,GNAS,GTF3C2,HDGF,HMGA2,IKBKE,IL6ST,IRF9,KDM7A,LIMS1,MAML2,MBD1,METTL23,MGA,MLF1,NAB1,NEK4,NEK6,NFATC3,NFKBID,NLRC5,NOD2,NONO,NR3C1,PARP9,PLAC8,PLEKHG2,PPP1CC,PPP2CA,PREB,PRKAR1A,PRKCB,RBAK,RBCK1,RBL1,RCOR1,RPAIN,S1PR1,SATB1,SMARCA5,SNIP1,SSRP1,STAT1,TAB2,TAF7,TCF7,THRAP3,TLE3,TNFSF8,TXK,UBE3A,UBN1,USP3,ZBP1,ZC3H8,ZFPM1,ZNF202,ZNF692
Cell death of blood cells	8.00E-05	0.206	37	ABCB1,ADAM8,APAF1,BIRC2,CCR7,CD2,CSF1,CSF1R,CTLA4,FASLG,FCMR,FCRL3,FOXO1,GLS,GNAS,GZMB,IKBKE,IL15,IL6ST,NF1,NFKBID,NR3C1,PDPK1,PIK3CD,PLAT,PLCG2,POLR3E,PRKCB,RICTOR,S1PR1,SATB1,ST6GAL1,STAT1,TCF7,TNFSF8,TPP2,ZC3H8
Arrest in proliferation of tumor cell lines	8.09E-05		11	CBX7,GLS,IL6ST,IPO7,NR3C1,PPP1CC,RASSF5,RBL1,SKP2,STAT1,TAF7
Cell death of tumor cells	8.18E-05	-1.057	27	APAF1,BIRC2,BRMS1,COPB2,CSF1,CTLA4,EIF3G,FASLG,FAU,FOXO1,GZMB,HMGA2,IGHG1,IL15,IL6ST,LYPLA1,NEK4,NR3C1,PIK3CD,PPP2CA,PSMC6,PSMD12,RBM8A,RPA2,RPL19,SELL,SF3B3
Cytogenetically abnormal acute myeloid leukemia	8.63E-05		9	ABCB1,CD2,CSF1R,DHFR,DNMT3A,NR3C1,POLB,POLE,RRM1
Sezary syndrome	8.63E-05		9	CD2,CXCL13,DHFR,IGFBP4,IL15,KIR3DL1,POLE,RRM1,SATB1
Quantity of antigen presenting cells	8.71E-05	-0.811	19	ADAM8,CCR7,CD200,CD302,CSF1,CSF1R,CTLA4,CXCL13,ELMO1,GNAS,IL15,IL6ST,PILRA,PLAT,RICTOR,SELL,ST6GAL1,TIGIT,ZBTB46
Cellular homeostasis	9.06E-05	1.721	73	ACOT11,ADRB2,APAF1,ARHGAP35,ATP13A2,ATP2C1,ATP6V1A,BIRC2,CCR7,CD2,CTLA4,CXCL13,CXCR5,DMPK,EEF2,ELK1,FASLG,FBXL20,FFAR3,FOXO1,GJA4,GNA13,GNAQ,GNAS,GZMB,HSPA4,IGFBP4,IGHG1,IL15,IL6ST,LONP1,MMP1,NAB1,NF1,NFATC3,NFKBID,NOD2,NR3C1,PDE3B,PDPK1,PIK3CD,PLCG2,PLD1,PLEKHF1,POLB,PPP2CA,PRKCB,RAB12,RAPGEF2,RBL1,RICTOR,S1PR1,SATB1,SELENON,SKP2,SLC11A2,SLC25A14,SLC27A1,SLC4A10,STAT1,TAB2,TAB3,TCF7,TFRC,TNFSF8,TPP2,TRIM5,TXK,VMP1,ZACN,ZBTB24,ZC3H8,ZFPM1
Function of mononuclear leukocytes	9.19E-05	-1.076	20	ABCB1,ADRB2,CCR7,CSF1,CTLA4,IL15,NEDD9,NFKBID,NLRC5,NOD2,PIK3CD,PLCG2,RASSF5,S1PR1,SELL,SKP2,STAT1,TCF7,TPP2,TXK

Apoptosis of lymphocytes	9.26E-05	-0.140	22	ADAM8,CD2,CSF1,CTLA4,FASLG,FCMR,GNAS,GZMB,IKBKE,IL15,NFKBID,NR3C1,PDPK1,PIK3CD,PLCG2,POLR3E,PRKCB,S1PR1,SATB1,STAT1,TPP2,ZC3H8
Quantity of cells	9.33E-05	0.277	79	ABCB1,ADAM8,ADRB2,APAF1,ARX,ATP2C1,BIRC2,BRD1,CCR7,CD200,CD302,CREB3L2,CSF1,CSF1R,CTLA4,CXCL13,CXCR5,DAPP1,DHFR,DKC1,DNMT3A,DYNLL1,EDA,EIF4EBP2,ELMO1,FANCD2,FASLG,FCMR,FFAR3,FOXO1,GLS,GNA13,GNAQ,GNAS,GPC3,HDGF,HMGA2,IGHG1,IL15,IL6ST,MATK,NAT10,NEDD9,NF1,NFATC3,NLRC5,NOD2,NR3C1,PIK3CD,PILRA,PLAC8,PLAT,PLCG2,POLR3E,PPP2CA,PRKCB,RAPGEF2,RASSF2,RASSF5,RBL1,RBM38,RCOR1,RICTOR,S1PR1,SATB1,SELENON,SELL,SKP2,SLC11A2,SMN1/SMN2,SNX27,ST6GAL1,STAT1,TFRC,TIGIT,TPP2,TXK,UBE3A,ZBTB46
M1 childhood acute myeloid leukemia	9.72E-05		5	ABCB1,DHFR,NR3C1,POLB,RRM1
M2 childhood acute myeloid leukemia	9.72E-05		5	ABCB1,DHFR,NR3C1,POLB,RRM1
M4 childhood acute myeloid leukemia	9.72E-05		5	ABCB1,DHFR,NR3C1,POLB,RRM1
M5a childhood acute myeloid leukemia	9.72E-05		5	ABCB1,DHFR,NR3C1,POLB,RRM1
M5b childhood acute monocytic leukemia	9.72E-05		5	ABCB1,DHFR,NR3C1,POLB,RRM1
M6 childhood acute erythroid leukemia	9.72E-05		5	ABCB1,DHFR,NR3C1,POLB,RRM1
M7 childhood acute myeloid leukemia	9.72E-05		5	ABCB1,DHFR,NR3C1,POLB,RRM1
Fibrosis	1.05E-04	-1.027	30	ADRB2,AGO2,CSF1,CSF1R,CXCL13,DHFR,DNMT3A,ELK1,FASLG,GNAS,HDGF,HMGA2,IGHG1,IKBKE,IL6ST,LIMS1,MMP1,NDUFS6,NF1,NR3C1,PILRA,PLAC8,PLAT,PNKD,POLB,PRKCB,RASSF2,SELL,SKP2,STAT1
Inflammation of joint	1.07E-04	-1.662	52	ABCB1,ADAM8,ADRB2,AIF1,APAF1,ARIH1,ATP2B1,ATP2C1,BRMS1,CCR7,CD200,CSF1,CSF1R,CTLA4,CXCL13,CXCR5,DHFR,DYNLL1,EEF2,EVC,FASLG,FAU,FCRL3,FDPS,FOXO1,GZMB,IGFBP4,IGHG1,IL15,IL6ST,KIR3DL1,MBD1,MMP1,NOD2,NONO,NR3C1,PDE3B,PIK3CD,PILRA,PLAT,PLCG2,POLB,PPFIA1,PRKCB,RBM38,RPL19,S1PR1,SKIV2L,STAT1,TCF7,TFRC,TLE3
Abnormal morphology of lymphoid organ	1.32E-04		23	CCR7,CD200,CSF1,CSF1R,CTLA4,CXCR5,FASLG,GPC3,IL15,IL6ST,NFATC3,NLRC5,NOD2,PIK3CD,RASSF2,RASSF5,RBL1,RBM38,SATB1,SELL,SNX27,TPP2,ZBTB46
Apoptosis of lymphatic system cells	1.32E-04	0.047	23	ADAM8,CD2,CSF1,CTLA4,FASLG,FCMR,GNAS,GZMB,IKBKE,IL15,IL6ST,NFKBID,NR3C1,PDPK1,PIK3CD,PLCG2,POLR3E,PRKCB,S1PR1,SATB1,STAT1,TPP2,ZC3H8
Cell death of mononuclear leukocytes	1.32E-04	0.561	24	ADAM8,APAF1,CD2,CSF1,CTLA4,FASLG,FCMR,GNAS,GZMB,IKBKE,IL15,IL6ST,NFKBID,NR3C1,PDPK1,PIK3CD,PLCG2,POLR3E,PRKCB,S1PR1,SATB1,STAT1,TPP2,ZC3H8
Cell viability of mononuclear leukocytes	1.38E-04	1.288	15	CSF1,CSF1R,CTLA4,FASLG,FCMR,FCRL3,IL15,IL6ST,PDPK1,PIK3CD,PLCG2,PRKCB,RICTOR,STAT1,TCF7
T-cell leukemia	1.42E-04		17	CCR7,CD2,CSF1R,CXCL13,DHFR,DNMT3A,GZMB,NF1,NR3C1,POLB,POLE,PRKCB,RRM1,S1PR1,SELL,TCF7,VMP1
Cell viability	1.49E-04	↑2.969	62	ABCB1,AGO2,AGPS,APAF1,ATP13A2,ATP2C1,BIRC2,CASK,CCR7,CD200,CKS1B,COPB2,CSF1,CSF1R,CTLA4,EDA,EEF2,EIF4A3,ELK1,FANCD2,FASLG,FCMR,FCRL3,FOXO1,GNA13,HDGF,HMGA2,IKBKE,IL15,IL6ST,IRF9,MCFD2,MMP1,NEK4,NF1,NR3C1,PDPK1,PIK3CD,PLAC8,PLAT,PLCG2,PLEKHG2,POLB,PPP1CC,PPP2CA,PRKAR1A,PRKCB,RICTOR,RRM1,S1PR1,SELL,SHPRH,SLC11A2,SLC22A2,SMN1/SMN2,SPRY1,STAT1,TCF7,TERF1,TNFSF8,VBP1,WTAP
Cell death of connective tissue cells	1.51E-04	-0.871	33	ABCB1,APAF1,BIRC2,CD2,CREB3L2,CSF1,CSF1R,ELK1,FANCD2,FASLG,FOXO1,GLS,GNA13,GNAQ,GNL3,GZMB,MDN1,NFKBID,NR3C1,PLAC8,PLEKHF1,POLB,PRKAR1A,PRKCB,RASA4,RASSF5,RBL1,S1PR1,SKP2,STAT1,TPP1,TPP2,YME1L1
Chemotaxis of lymphocytes	1.59E-04	↑2.279	11	ADAM8,CCR7,CXCL13,ELMO1,GNA13,GNAS,IL15,NEDD9,NR3C1,PIK3CD,S1PR1
Hematopoiesis of mononuclear leukocytes	1.65E-04	1.831	37	APAF1,CCR7,CD2,CSF1,CSF1R,CTLA4,CXCR5,ELK1,FASLG,FOXO1,IGHG1,IL15,IL6ST,LONP1,NAB1,NF1,NFATC3,NFKBID,PDPK1,PIK3CD,PLCG2,POLB,RASSF5,RBL1,RICTOR,S1PR1,SATB1,SKP2,STAT1,TAB2,TAB3,TCF7,TNFSF8,TPP2,TXK,ZBTB46,ZFPM1
Neoplasia of leukocytes	1.71E-04		30	CCR7,CD2,CD200,CSF1R,CXCL13,DHFR,DNMT3A,FASLG,GJA4,GZMB,IFITM1,IGFBP4,IL15,KIR3DL1,MMP1,NF1,NR3C1,OAS2,POLB,POLE,PRKCB,RRM1,S1PR1,SATB1,SELL,SKP2,STAT1,TCF7,VMP1,ZBTB24
Graft-vs-host disease	1.72E-04		8	CD2,DHFR,FASLG,KIR3DL1,NR3C1,POLB,RRM1,TNFSF8
Abnormal morphology of spleen	1.72E-04		18	CCR7,CSF1,CSF1R,CTLA4,FASLG,GPC3,IL6ST,NFATC3,NLRC5,PIK3CD,RASSF2,RASSF5,RBL1,RBM38,SATB1,SELL,SNX27,TPP2
Organization of lymphoid tissue	1.74E-04		3	CCR7,CXCL13,CXCR5
Quantity of hematopoietic progenitor cells	1.85E-04	↑2.291	26	ABCB1,BRD1,CCR7,CSF1,CTLA4,FANCD2,FOXO1,GPC3,IL15,IL6ST,MATK,NOD2,NR3C1,PIK3CD,PLCG2,POLR3E,RAPGEF2,RASSF2,RASSF5,RBM38,RCOR1,SATB1,ST6GAL1,TFRC,TPP2,TXK
B-cell lymphoma	1.86E-04		38	AKAP8,ATRN,CCR7,CHD6,CRNN,CSF1R,CXCL13,CXCR5,DHFR,ELK1,FILIP1L,FOXO1,GNA13,LONP1,MBD1,MGA,NEK4,NF1,NONO,NR3C1,OSBP,PIK3CD,PLCG2,PLEKHG3,POLB,POLE,PPP2CA,PRKCB,RAB4A,RASSF5,RBCK1,RICTOR,RRM1,SATB1,SKIV2L2,SLC4A10,TNFSF8,TNRC6C
Quantity of dendritic cells	1.92E-04	-0.489	11	CCR7,CD200,CD302,CSF1,CSF1R,CTLA4,CXCL13,IL15,RICTOR,SELL,ZBTB46

Progression of tumor	1.93E-04	0.239	18	BRMS1,CKS1B,CSF1,CSF1R,CTLA4,DHFR,FOXO1,GZMB,IGFBP4,MMP1,NEDD9,NR3C1,PIK3CD,POLB,POLE,RRM1,SKP2,STAT1
Apoptosis of T lymphocytes	2.00E-04	0.228	18	ADAM8,CD2,CTLA4,FASLG,FCMR,GNAS,GZMB,IKBKE,IL15,NFKBID,NR3C1,PDPK1,PIK3CD,POLR3E,SATB1,STAT1,TPP2,ZC3H8
Cell death of lymphocytes	2.03E-04	0.355	23	ADAM8,APAF1,CD2,CSF1,CTLA4,FASLG,FCMR,GNAS,GZMB,IKBKE,IL15,NFKBID,NR3C1,PDPK1,PIK3CD,PLCG2,POLR3E,PRKCB,S1PR1,SATB1,STAT1,TPP2,ZC3H8
Leukocytosis	2.04E-04	0.896	11	BIRC2,CSF1,CSF1R,FASLG,IL15,IL6ST,PIK3CD,POLB,POLE,RRM1,SELL
Mammary tumor	2.05E-04	0.054	77	ABCB1,ADRB2,AGO2,AGPS,APH1A,ARHGAP35,ATP2C1,BRWD1,C17orf49,CASK,CCNL1,CCR7,CMAHP,CNTN1,CSF1,CSF1R,CTLA4,DBF4,DHFR,DKC1,DNMT3A,ETFA,FANCD2,FDPS,GGCT,GLS,GNA13,GNAS,GOSR1,GPC3,HMGA2,IKBKE,ILKAP,KLHL12,LONP1,LYPLA1,MBD1,MCM6,MGA,MLF1,MMP1,MUC7,NEDD9,NEK4,NEK6,NF1,NR3C1,PDPK1,PIK3CD,PLAT,PNPO,POLB,POLE,PPFIA1,PPP1CC,PRKCB,PSMD12,RPL19,RRM1,SELL,SETD1B,SF3B3,SKP2,SLC25A14,SLC27A1,SLC5A10,SSTR3,STAG2,STAT1,TES,TLE3,UBAP2L,UBE3A,VBP1,VWA5A,ZFPM1,ZNF692
B-cell non-Hodgkin disease	2.08E-04		36	AKAP8,ATRN,CCR7,CHD6,CRNN,CSF1R,CXCL13,CXCR5,DHFR,ELK1,FILIP1L,FOXO1,GNA13,LONP1,MBD1,MGA,NEK4,NF1,NONO,NR3C1,OSBP,PIK3CD,PLCG2,PLEKHG3,POLB,POLE,PPP2CA,PRKCB,RAB4A,RBCK1,RICTOR,RRM1,SATB1,SKIV2L2,SLC4A10,TNRC6C
Systemic autoimmune syndrome	2.13E-04	0.351	53	ABCB1,ADAM8,ADRB2,AGFG1,AIF1,ARIH1,ATP2B1,ATP2C1,BRMS1,CCR7,CMAHP,CSF1,CTLA4,CXCL13,CXCR5,DHFR,DYNLL1,EEF2,EVC,FASLG,FAU,FCMR,FCRL3,FDPS,FOXO1,GNAQ,GZMB,IGFBP4,IGHG1,IL15,IL6ST,MBD1,MMP1,NOD2,NONO,NR3C1,P2RY10,POLB,PPP2CA,PRKCB,RBM38,RPL19,S1PR1,SELL,SKIV2L,ST6GAL1,STAT1,TCF7,TFRC,TLE3,TNFSF8,TPP2,ZBP1
Quantity of phagocytes	2.14E-04	-1.881	25	ABCB1,ADAM8,CCR7,CD200,CD302,CSF1,CSF1R,CTLA4,CXCL13,ELMO1,FASLG,GNAS,IL15,IL6ST,NF1,NOD2,PILRA,PLAC8,PLAT,PPP2CA,RICTOR,SELL,ST6GAL1,TIGIT,ZBTB46
Abnormal morphology of dendritic cells	2.18E-04		5	CSF1R,CXCR5,PDPK1,PLCG2,ZBTB46
Trafficking of T lymphocytes	2.18E-04	1.342	5	CCR7,FOXO1,PDPK1,S1PR1,SELL
Homing of leukocytes	2.22E-04	↑2.010	23	ADAM8,AIF1,CCR7,CSF1,CSF1R,CXCL13,CXCR5,ELMO1,FASLG,FOXO1,GNA13,GNAS,IGHG1,IL15,NEDD9,NOD2,NR3C1,PIK3CD,PLCG2,PRKCB,RICTOR,S1PR1,SELL
Function of leukocytes	2.27E-04	-1.076	27	ABCB1,ADRB2,CCR7,CD200,CSF1,CSF1R,CTLA4,FCMR,IL15,NEDD9,NF1,NFKBID,NLRC5,NOD2,PIK3CD,PLAT,PLCG2,RASSF5,S1PR1,SELL,SKP2,ST6GAL1,STAT1,TCF7,TIGIT,TPP2,TXK
Diffuse lymphoma	2.35E-04		31	ADSL,AKAP8,CD2,CHD6,CRNN,CSF1R,DHFR,ELK1,FILIP1L,FOXO1,GNA13,IKBKE,LONP1,MBD1,MGA,NEK4,NF1,NR3C1,OSBP,PIK3CD,PLCG2,PLEKHG3,POLB,POLE,RBCK1,RRM1,SKIV2L2,SLC4A10,TNRC6C,UBE3A,UROS
Function of lymphocytes	2.37E-04	-0.813	19	ABCB1,ADRB2,CCR7,CTLA4,IL15,NEDD9,NFKBID,NLRC5,NOD2,PIK3CD,PLCG2,RASSF5,S1PR1,SELL,SKP2,STAT1,TCF7,TPP2,TXK
T cell homeostasis	2.40E-04	↑2.001	30	APAF1,CCR7,CD2,CTLA4,CXCR5,ELK1,FOXO1,IGHG1,IL15,IL6ST,LONP1,NAB1,NF1,NFATC3,NFKBID,PDPK1,PIK3CD,POLB,RBL1,RICTOR,S1PR1,SATB1,SKP2,STAT1,TCF7,TNFSF8,TPP2,TXK,ZC3H8,ZFPM1
Hematologic cancer of cells	2.43E-04		29	CCR7,CD2,CD200,CSF1R,CXCL13,DHFR,DNMT3A,GJA4,GZMB,IFITM1,IGFBP4,IL15,KIR3DL1,MMP1,NF1,NR3C1,OAS2,POLB,POLE,PRKCB,RRM1,S1PR1,SATB1,SELL,SKP2,STAT1,TCF7,VMP1,ZBTB24
T-cell lymphoproliferative disorder	2.49E-04		29	CCR7,CD2,CD200,CSF1R,CXCL13,DHFR,DNMT3A,GJA4,GZMB,IFITM1,IGFBP4,IL15,KIR3DL1,MMP1,NF1,NR3C1,OAS2,POLB,POLE,PRKCB,RRM1,S1PR1,SATB1,SELL,SKP2,STAT1,TCF7,VMP1,ZBTB24
Function of regulatory T lymphocytes	2.58E-04	-0.832	6	ADRB2,CTLA4,PIK3CD,S1PR1,SELL,SKP2
Cell death of fibroblast cell lines	2.67E-04	-1.027	25	ABCB1,APAF1,BIRC2,CD2,CSF1R,ELK1,FANCD2,FASLG,GLS,GNA13,GNAQ,GZMB,MDN1,NR3C1,PLAC8,PLEKHF1,POLB,PRKAR1A,PRKCB,RASA4,RASSF5,RBL1,SKP2,STAT1,YME1L1
Cutaneous T-cell lymphoma	2.69E-04		8	CCR7,DHFR,IFITM1,IL15,NR3C1,OAS2,PRKCB,STAT1
Cell movement of lymphatic system cells	2.74E-04	↑2.192	25	ADAM8,CCR7,CD2,CTLA4,CXCL13,CXCR5,ELMO1,FASLG,FOXO1,GNA13,GNAS,IGHG1,IL15,IL6ST,NEDD9,NR3C1,PDPK1,PIK3CD,PLCG2,RASSF5,RICTOR,S1PR1,SELL,STAT1,TXK
T-cell non-Hodgkin disease	2.75E-04		24	CCR7,CD2,CD200,CXCL13,DHFR,DNMT3A,GJA4,GZMB,IFITM1,IGFBP4,IL15,KIR3DL1,MMP1,NR3C1,OAS2,POLB,POLE,PRKCB,RRM1,SATB1,SELL,SKP2,STAT1,ZBTB24
Binding of blood cells	2.89E-04	1.878	25	ADRB2,ATRN,CCR7,CD2,CD200,CSF1,CTLA4,CXCL13,FASLG,FOXO1,GZMB,IL15,IL6ST,NEDD9,NFATC3,NR3C1,PIK3CD,PILRA,PLCG2,RASSF5,RICTOR,SELL,STAT1,TFRC,TXK
Apoptosis of cervical cancer cell lines	2.92E-04	-1.442	19	ABCB1,BIRC2,CLASP1,CSF1R,FASLG,GZMB,IKBKE,LIMS1,LYPLA1,NEDD9,NEK6,NR3C1,PDPK1,RICTOR,STAT1,TERF1,TRAP1,UBA7,VOPP1
Stage I-IV B-cell non-Hodgkin lymphoma	2.94E-04		6	DHFR,NR3C1,PIK3CD,POLB,POLE,RRM1
Cell movement of leukemia cell lines	2.98E-04	0.909	12	AIF1,CSF1,CXCL13,FASLG,GNA13,MATK,NEDD9,PLD1,PRKCB,RICTOR,S1PR1,SELL
Cell movement of lymphocytes	2.99E-04	↑2.453	24	ADAM8,CCR7,CD2,CTLA4,CXCL13,CXCR5,ELMO1,FASLG,FOXO1,GNA13,GNAS,IGHG1,IL15,IL6ST,NEDD9,NR3C1,PDPK1,PIK3CD,RASSF5,RICTOR,S1PR1,SELL,STAT1,TXK
Small cell lymphocytic lymphoma/chronic lymphocytic leukemia	3.03E-04		33	AKAP8,CD200,CEP68,CHD6,CRNN,CSF1R,DLEU1,ELK1,FASLG,FCMR,FILIP1L,LONP1,LYPLA1,MBD1,MGA,NEDD9,NR3C1,OSBP,PDE3B,PIK3CD,PLCG2,PNKD,POLB,POLE,PRKCB,RASSF5,RBCK1,RRM1,S1PR1,SKIV2L2,SLC4A10,TIGIT,UCKL1

Cell death of lymphatic system cells	3.07E-04	0.527	24	ADAM8,APAF1,CD2,CSF1,CTLA4,FASLG,FCMR,GNAS,GZMB,IKBKE,IL15,IL6ST,NFKBID,NR3C1,PDPK1,PIK3CD,PLCG2,POLR3E,PRKCB,S1PR1,SATB1,STAT1,TPP2,ZC3H8
Acute myeloid leukemia with inv(16)	3.09E-04		5	ABCB1,CD2,DHFR,POLB,RRM1
Deletion of T lymphocytes	3.09E-04		5	CD2,CTLA4,FASLG,IL15,TCF7
FLT3 internal tandem duplication positive acute myeloid leukemia	3.09E-04		5	CSF1R,NR3C1,POLB,POLE,RRM1
Relapsed diffuse large B-cell lymphoma	3.09E-04		5	NR3C1,PIK3CD,POLB,POLE,RRM1
Cell movement of mononuclear leukocytes	3.33E-04	↑ 2.512	27	ADAM8,AIF1,ATRN,CCR7,CD2,CTLA4,CXCL13,CXCR5,ELMO1,FASLG,FOXO1,GNA13,GNAS,IGHG1,IL15,IL6ST,NEDD9,NR3C1,PDPK1,PIK3CD,PRKCB,RASSF5,RICTOR,S1PR1,SELL,STAT1,TXK
Philadelphia chromosome positive leukemia	3.39E-04		7	CSF1R,DHFR,GNAQ,NR3C1,POLB,POLE,RRM1
Survival of organism	3.64E-04	1.990	37	ABCB1,ADRB2,ATP2C1,BABAM1,CD2,CNTN1,CSF1,CTLA4,DNMT3A,EPN1,FANCD2,FASLG,FCMR,FOXO1,GNA13,GNAS,HMGA2,IKBKE,IL15,IL6ST,NFATC3,NLRC5,NOD2,NR3C1,PDPK1,PIK3CD,PLAT,PLD1,PRKAR1A,PRKCB,RBL1,SKP2,SLC22A2,SMN1/SMN2,SNX27,STAT1,TXK
17p deletion positive TP53 mutation positive chronic lymphocytic leukemia	3.64E-04		4	NR3C1,PIK3CD,POLB,RRM1
Binding of interferon-stimulated response element	3.64E-04	1.982	4	IKBKE,IRF9,RICTOR,STAT1
Cell movement of naive lymphocytes	3.64E-04	1.941	4	CCR7,CXCL13,CXCR5,SELL
T cell development	3.65E-04	↑ 2.016	29	APAF1,CCR7,CD2,CTLA4,CXCR5,ELK1,FOXO1,IGHG1,IL15,IL6ST,LONP1,NAB1,NF1,NFATC3,NFKBID,PDPK1,PIK3CD,POLB,RBL1,RICTOR,S1PR1,SATB1,SKP2,STAT1,TCF7,TNFSF8,TPP2,TXK,ZFPM1
Cell death of T lymphocytes	3.82E-04	0.578	19	ADAM8,APAF1,CD2,CTLA4,FASLG,FCMR,GNAS,GZMB,IKBKE,IL15,NFKBID,NR3C1,PDPK1,PIK3CD,POLR3E,SATB1,STAT1,TPP2,ZC3H8
B-cell malignant tumor	4.00E-04		39	AKAP8,ATRN,CCR7,CHD6,CRNN,CSF1R,CXCL13,CXCR5,DHFR,ELK1,FILIP1L,FOXO1,GNA13,LONP1,MBD1,MGA,NEK4,NF1,NONO,NR3C1,OSBP,PIK3CD,PLCG2,PLEKHG3,POLB,POLE,PPP2CA,PRKCB,RAB4A,RASSF5,RBCK1,RICTOR,RRM1,SATB1,SKIV2L2,SLC4A10,TFRC,TNFSF8,TNRC6C
Cortical T cell acute lymphoblastic leukemia	4.12E-04		7	CD2,CSF1R,DHFR,NR3C1,POLB,POLE,RRM1
Histiocytosis	4.12E-04		7	CSF1R,DHFR,MMP1,NR3C1,POLB,POLE,RRM1
Burkitt's lymphoma	4.17E-04		10	ADSL,DHFR,GNA13,IKBKE,NR3C1,POLB,POLE,RRM1,UBE3A,UROS
Quantity of follicular B lymphocytes	4.17E-04	0.997	10	FCMR,FOXO1,GNA13,IL6ST,NEDD9,NFATC3,PIK3CD,PLCG2,ST6GAL1,STAT1
Differentiation of osteoclasts	4.23E-04	-0.733	14	ADAM8,ADRB2,CSF1,CSF1R,DYNLL1,GNAS,GPC3,IGHG1,IL6ST,NF1,PLCG2,RASSF2,STAT1,TFRC
Childhood myelodysplastic syndrome	4.27E-04		5	ABCB1,DHFR,NR3C1,POLB,RRM1
De novo myelodysplastic syndrome	4.27E-04		5	ABCB1,DHFR,NR3C1,POLB,RRM1
Acquired microcephaly	4.38E-04		2	ARX,PLEKHG2
Development of B-cell follicle	4.38E-04		2	CCR7,CXCR5
Entry into cell cycle progression of fibroblasts	4.38E-04		2	RBL1,SKP2
Formation of kinetochore microtubule	4.38E-04		2	NEK6,UBAP2L
Low grade malignant urothelial neoplasm	4.38E-04		2	FOXO1,STAG2
Moderate grade chronic obstructive pulmonary disease	4.38E-04		2	ADRB2,NR3C1
Retroperitoneal fibrosis	4.38E-04		2	DHFR,NR3C1
Trafficking by naive T lymphocytes	4.38E-04		2	CCR7,SELL
11q deletion positive 17p deletion negative TP53 mutation negative chronic lymphocytic leukemia	4.61E-04		4	NR3C1,PIK3CD,POLB,RRM1

Rheumatoid arthritis	4.72E-04		39	ABCB1,ADAM8,ADRB2,AIF1,ARIH1,ATP2B1,ATP2C1,BRMS1,CCR7,CSF1,CTLA4,CXCL13,CXCR5,DHFR,DYNLL1,EEF2,EVC,FASLG,FAU,FCRL3,FDPS,FOXO1,GZMB,IGFBP4,IGHG1,IL15,IL6ST,MBD1,MMP1,NONO,NR3C1,POLB,RBM38,RPL19,S1PR1,SKIV2L,STAT1,TFRC,TLE3
Morphology of spleen	4.81E-04		22	CCR7,CSF1,CSF1R,CTLA4,CXCR5,FASLG,GPC3,IGHG1,IL15,IL6ST,NFATC3,NLRC5,PDPK1,PIK3CD,RASSF2,RASSF5,RBL1,RBM38,SATB1,SELL,SNX27,TPP2
Quantity of lymphoid organ	4.85E-04	1.776	18	ABCB1,CCR7,CD200,CTLA4,CXCL13,CXCR5,FOXO1,NOD2,NR3C1,PIK3CD,POLR3E,RASSF5,RBM38,RICTOR,SATB1,STAT1,TPP2,TXK
Lymphopoiesis	4.89E-04	↑ 2.201	34	APAF1,CCR7,CD2,CTLA4,CXCR5,ELK1,FASLG,FOXO1,IGHG1,IL15,IL6ST,LONP1,NAB1,NF1,NFATC3,NFKBID,PDPK1,PIK3CD,PLCG2,POLB,RASSF5,RBL1,RICTOR,S1PR1,SATB1,SKP2,STAT1,TAB2,TAB3,TCF7,TNFSF8,TPP2,TXK,ZFPM1
Refractory Philadelphia chromosome-negative acute lymphoblastic leukemia	4.98E-04		5	DHFR,NR3C1,POLB,POLE,RRM1
Relapsed Philadelphia chromosome-negative acute lymphoblastic leukemia	4.98E-04		5	DHFR,NR3C1,POLB,POLE,RRM1
Viral Infection	5.00E-04	↑ 3.445	69	ABCB1,ADRB2,AGFG1,AGO2,APAF1,ATP2C1,ATP6V1A,CCR7,CD200,CEP68,CLTB,COG3,COPB2,CSF1,CTLA4,DCAF13,DHFR,DMPK,DNMT3A,EDEM1,EIF3G,EIF4A3,EIF4EBP2,EPN1,FASLG,FAU,FDPS,HSPA4,IFITM1,IKBKE,IL15,IRF9,KDM7A,LONP1,MDN1,NDUFS6,NLRC5,NR3C1,OAS2,OSBP,OSBPL3,P2RY10,PARP9,PDE3B,PHF3,PLAT,PLCG2,PLD1,POLB,PRKCB,PSMD12,PVT1,RPL18,RRM1,SELL,SETD1B,SMN1/SMN2,SSRP1,ST6GAL1,STAT1,STT3A,SYNJ1,TFRC,TRIM14,TRIM5,UBA7,VNN2,ZBP1,ZCCHC17
Invasion of cells	5.13E-04	1.645	43	ADAM8,ADI1,ADRB2,AGO2,ARHGEF18,CCR7,COPB2,CSF1,CUL4B,ELK1,ELMO1,FASLG,FOXO1,GEM,GNA13,GNAQ,GNAS,GZMB,HDGF,HMGA2,IFITM1,IGHG1,IPO7,LDLRAP1,LYPLA1,MATK,MMP1,NEDD9,NF1,NFKBID,NOD2,PDPK1,PIK3CD,PLAT,PLD1,PRKCB,RICTOR,RRM1,SATB1,SKP2,ST6GAL1,STAT1,UBE3A
Activation of blood cells	5.24E-04	0.415	36	CCR7,CD2,CD200,CSF1,CSF1R,CTLA4,CXCR5,DAPP1,FASLG,FCRL3,FOXO1,GJA4,GNA13,GNAQ,HSPA4,IGHG1,IL15,KIR3DL1,MMP1,NFATC3,NFKBID,NOD2,PIK3CD,PLAT,PLCG2,PRKCB,RAB4A,SATB1,SELL,SIRPG,ST6GAL1,STAT1,TAB2,TAB3,TIGIT,ZBTB46
Cell cycle progression	5.30E-04	0.148	49	AIF1,AKAP8,APAF1,ARX,BIRC2,BUB3,CACUL1,CBX7,CKAP2,CLASP1,CSF1,CSF1R,CTLA4,DBF4,DHFR,DYNC1LI2,ELK1,FANCD2,FOXO1,GEM,GNA13,GNL3,IGHG1,IRF9,MATK,MLF1,NEDD9,NEK4,NEK6,NF1,NR3C1,PDPK1,PDS5A,PLAC8,PPP2CA,PRKAR1A,PRKCB,RASSF2,RASSF5,RBL1,RRM1,S1PR1,SKP2,SSRP1,STAT1,TERF1,TPP2,UBA7,USP3
Chemotaxis of mononuclear leukocytes	5.32E-04	↑ 2.440	13	ADAM8,AIF1,CCR7,CXCL13,ELMO1,GNA13,GNAS,IL15,NEDD9,NR3C1,PIK3CD,PRKCB,S1PR1
Refractory acute lymphocytic leukemia	5.33E-04		6	CSF1R,DHFR,NR3C1,POLB,POLE,RRM1
Apoptosis of lung cancer cell lines	5.41E-04	-1.075	15	APAF1,FASLG,GNAS,IKBKE,IL6ST,ILKAP,NR3C1,PPP2CA,PRKCB,RICTOR,RPA2,RRM1,SKP2,SSRP1,TRAP1
Development of hematopoietic progenitor cells	5.44E-04	1.077	16	AGO2,BRD1,CSF1,FOXO1,HMGA2,IL15,IL6ST,NAB1,PLCG2,RCOR1,RICTOR,SATB1,SMARCA5,STAT1,TCF7,ZFPM1
Urothelial bladder carcinoma	5.69E-04		11	ARHGAP35,CHD6,CTLA4,DHFR,FOXO1,NF1,NLRC5,POLE,PRKCB,RRM1,STAG2
Stage I-IV diffuse large B-cell lymphoma	5.77E-04		5	DHFR,NR3C1,POLB,POLE,RRM1
Cell viability of antigen presenting cells	5.94E-04	0.904	7	CCR7,CSF1,CSF1R,FOXO1,IL15,PIK3CD,PLAT
Cell death of thymocytes	5.99E-04	0.510	11	ADAM8,APAF1,FASLG,GNAS,GZMB,NFKBID,NR3C1,PIK3CD,POLR3E,SATB1,ZC3H8
Cell death of lymphoid organ	6.02E-04	0.734	12	ADAM8,APAF1,FASLG,GNAS,GZMB,IL6ST,NFKBID,NR3C1,PIK3CD,POLR3E,SATB1,ZC3H8
Chemotaxis of leukocytes	6.37E-04	1.947	21	ADAM8,AIF1,CCR7,CSF1,CSF1R,CXCL13,CXCR5,ELMO1,FASLG,GNA13,GNAS,IL15,NEDD9,NOD2,NR3C1,PIK3CD,PLCG2,PRKCB,RICTOR,S1PR1,SELL
Relapsed childhood acute lymphoblastic leukemia	6.62E-04		6	CSF1R,DHFR,NR3C1,POLB,POLE,RRM1
Apoptosis of tumor cell lines	6.93E-04	-1.436	55	ABCB1,ADI1,APAF1,BIRC2,BUB3,CKAP2,CLASP1,CSF1R,DYNLL1,FASLG,FAU,FCMR,FOXO1,GEM,GLS,GNA13,GNAS,GZMB,HSPA4,IGFBP4,IGHG1,IKBKE,IL15,IL6ST,ILKAP,KIR3DL1,LIMS1,LYPLA1,NAT10,NEDD9,NEK6,NF1,NR3C1,PDPK1,PIK3CD,PLAT,PLCG2,PPP2CA,PRKAR1A,PRKCB,RAPGEF2,RASSF5,RICTOR,RPA2,RRM1,SATB1,SKP2,SSRP1,ST6GAL1,STAT1,TERF1,TFRC,TRAP1,UBA7,VOPP1
Apoptosis of cytotoxic T cells	6.99E-04		3	FASLG,GZMB,IL15
Paternal imprinting	6.99E-04		3	GNAS,GPC3,UBE3A
Refractory childhood acute lymphoblastic leukemia	6.99E-04		3	POLB,POLE,RRM1
Granulomatosis	7.06E-04		4	DHFR,FDPS,NOD2,NR3C1
Binding of leukocytes	7.08E-04	↑ 2.119	22	ATRN,CCR7,CD2,CD200,CSF1,CTLA4,CXCL13,FASLG,FOXO1,IL15,IL6ST,NEDD9,NFATC3,NR3C1,PIK3CD,PILRA,RASSF5,RICTOR,SELL,STAT1,TFRC,TXK

Activation of cells	7.23E-04	0.175	45	ADAM8,CCR7,CD2,CD200,CSF1,CSF1R,CTLA4,CXCR5,DAPP1,FASLG,FCRL3,FFAR3,FOXO1,GJA4,GNA13,GNAQ,GNAS,GZMB,HSPA4,IGHG1,IL15,IL6ST,KIR3DL1,MMP1,NFATC3,NFKBID,NOD2,PDPK1,PIK3CD,PLAT,PLCG2,PPP2CA,PRKCB,RAB4A,RICTOR,SATB1,SELL,SIRPG,ST6GAL1,STAT1,TAB2,TAB3,TIGIT,ZBP1,ZBTB46
Thymic carcinoma	7.36E-04		6	CSF1R,DHFR,NR3C1,POLE,RRM1,SSTR3
Cell death of epithelial cell lines	7.64E-04	-1.143	18	AAK1,ATP13A2,BIRC2,CSF1R,CUL4B,DYNLL1,ELK1,FASLG,FOXO1,NEK6,PDPK1,PPP2CA,PRKCB,RASSF5,SLC22A2,SSRP1,TFRC,VOPP1
Non-M3 acute myeloid leukemia	7.69E-04		7	ABCB1,CSF1R,DHFR,DNMT3A,NR3C1,POLB,RRM1
Apoptosis of lymphoid organ	7.73E-04	0.425	11	ADAM8,FASLG,GNAS,GZMB,IL6ST,NFKBID,NR3C1,PIK3CD,POLR3E,SATB1,ZC3H8
Cell movement of cervical cancer cell lines	8.03E-04	0.853	9	ARHGAP35,CSF1R,DBF4,HDGF,NF1,PLD1,RICTOR,SNX27,ST6GAL1
Metabolism of DNA	8.08E-04	-1.342	22	ABCB1,APAF1,CD2,CSF1,CUL4B,DBF4,FASLG,FFAR3,GZMB,HMGA2,IGFBP4,NR3C1,PDS5A,POLB,POLE,PPP1CC,RPA2,RPAIN,SSRP1,STAT1,TERF1,VMP1
Advanced non-Hodgkin lymphoma	8.15E-04		6	DHFR,IL15,NR3C1,POLB,POLE,RRM1
Philadelphia-positive acute lymphoblastic leukemia	8.15E-04		6	CSF1R,DHFR,NR3C1,POLB,POLE,RRM1
Recurrent metastatic solid tumor	8.15E-04		6	CSF1R,CTLA4,DHFR,PIK3CD,POLE,RRM1
Myelodysplastic syndrome	8.38E-04		18	ABCB1,APAF1,APH1A,ARIH1,CSF1R,DHFR,DNMT3A,FANCD2,FASLG,GNAS,MGA,NF1,NR3C1,POLB,POLE,RRM1,STAG2,TLE3
SjÃ¶gren syndrome	8.53E-04		9	CCR7,CSF1,CXCL13,CXCR5,FASLG,IFITM1,MATK,NR3C1,STAT1
Inv(3) positive acute myeloid leukemia	8.57E-04		4	NR3C1,POLB,POLE,RRM1
Macular edema	8.57E-04		4	ADRB2,DHFR,NR3C1,PRKCB
Anemia	8.76E-04	-1.491	23	ADRB2,AGO2,BRD1,CTLA4,DHFR,DNMT3A,FANCD2,IL6ST,MTRR,NFATC3,NR3C1,PLCG2,POLB,RBM38,RRM1,SEC11A,SELL,SLC11A2,TFRC,TLE3,TPP1,TPP2,ZFPM1
Apoptosis of thymocytes	8.80E-04	0.170	10	ADAM8,FASLG,GNAS,GZMB,NFKBID,NR3C1,PIK3CD,POLR3E,SATB1,ZC3H8
Apoptosis of gonadal cell lines	9.01E-04		6	FASLG,GNAQ,PLD1,POLB,SSTR3,TFRC
Pallor	9.06E-04		9	BRD1,CUL4B,GNA13,NF1,POLB,SLC11A2,TAB2,TFRC,ZFPM1
Cell movement of natural killer cells	9.07E-04	1.422	7	CCR7,GNA13,GNAS,IL15,PIK3CD,S1PR1,SELL
Quantity of memory T lymphocytes	9.07E-04	-1.681	7	CTLA4,DNMT3A,IL15,RASSF5,STAT1,TPP2,TXK
Grade 1 lymphocytic cancer	9.48E-04		18	CCR7,CHD6,CRNN,CSF1R,CXCL13,CXCR5,ELK1,FILIP1L,MBD1,MGA,NR3C1,OSBP,PIK3CD,POLE,RBCK1,RRM1,SKIV2L2,SLC4A10
Cell death of cancer cells	9.62E-04	-1.516	21	APAF1,COPB2,CSF1,CTLA4,EIF3G,FASLG,FAU,FOXO1,IL15,IL6ST,LYPLA1,NEK4,NR3C1,PIK3CD,PSMC6,PSMD12,RBM8A,RPA2,RPL19,SELL,SF3B3
Cell viability of lymphocytes	9.66E-04	1.497	13	CTLA4,FASLG,FCMR,FCRL3,IL15,IL6ST,PDPK1,PIK3CD,PLCG2,PRKCB,RICTOR,STAT1,TCF7
Cytosis	9.70E-04	0.342	20	AGPS,ATP13A2,BIRC2,CSF1,CSF1R,DHFR,DNMT3A,FASLG,IL15,IL6ST,MMP1,NF1,NR3C1,PIK3CD,POLB,POLE,RRM1,S1PR1,SELL,SLC11A2
Adaptive immune response	9.82E-04	0.655	7	CCR7,CTLA4,FFAR3,FOXO1,IL15,PIK3CD,TXK
Response of embryonic cell lines	9.82E-04	1.195	7	BIRC2,EIF4EBP2,NOD2,RICTOR,SPRY1,TRIM14,TRIM5
Eradication of tumor	9.83E-04		3	CD200,CTLA4,IL15
Memory T cell response	9.83E-04		3	CTLA4,FOXO1,IL15
Morphology of lymphoid tissue	9.83E-04		28	CCR7,CD200,CSF1,CSF1R,CTLA4,CXCR5,FASLG,GNA13,GPC3,IGHG1,IL15,IL6ST,NF1,NFATC3,NLRC5,NOD2,NR3C1,PDPK1,PIK3CD,RASSF2,RASSF5,RBL1,RBM38,SATB1,SELL,SNX27,TPP2,ZBTB46
Cell death of lung cancer cell lines	9.87E-04	-1.257	16	APAF1,BIRC2,FASLG,GNAS,IKBKE,IL6ST,ILKAP,NR3C1,PPP2CA,PRKCB,RICTOR,RPA2,RRM1,SKP2,SSRP1,TRAP1
Activation of regulatory T lymphocytes	9.89E-04	0.555	5	CD2,CTLA4,IL15,NFATC3,PIK3CD
Refractory anemia with ringed sideroblasts	9.89E-04		5	DHFR,DNMT3A,NR3C1,POLB,RRM1
Immunosuppression	1.03E-03	0	4	ADRB2,CTLA4,NR3C1,S1PR1
Breast cancer	1.03E-03	-0.068	70	ABCB1,ADRB2,AGO2,APH1A,ARHGAP35,ATP2C1,BRWD1,C17orf49,CASK,CCNL1,CCR7,CMAHP,CNTN1,CSF1R,CTLA4,DHFR,DKC1,DNMT3A,ETFA,FANCD2,FDPS,GGCT,GLS,GNA13,GNAS,GOSR1,GPC3,HMGA2,IKBKE,ILKAP,KLHL12,LONP1,LYPLA1,MBD1,MCM6,MGA,MLF1,MMP1,MUC7,NEK6,NF1,NR3C1,PIK3CD,PLAT,PNPO,POLB,POLE,PPFIA1,PRKCB,PSMD12,RPL19,RRM1,SELL,SETD1B,SF3B3,SKP2,SLC25A14,SLC27A1,SLC5A10,SSTR3,STAG2,STAT1,TES,TLE3,UBAP2L,UBE3A,VBP1,VWA5A,ZFPM1,ZNF692
Organization of cytoplasm	1.04E-03	1.559	67	AGFG1,ARHGAP35,ARHGEF18,ATP13A2,ATP2B1,ATP2C1,ATRN,BRWD1,CARMIL1,CCR7,CD2,CHN1,CKAP2,CLASP1,CNTN1,COG3,COPB2,CSF1,CSF1R,CTLA4,CUL4B,DCTN6,DYNC1LI2,DYNLL1,EIF4EBP2,ELK1,ELMO1,GCC2,GEM,GNA13,GNAS,GZMB,HDGF,LONP1,MATK,MKS1,NEDD9,NEK6,NF1,PDE4DIP,PDPK1,PIK3CD,PLAT,PLCG2,PLD1,PLEKHF1,PPP2CA,PRKCB,RAB30,RAB43,RAPGEF2,RASSF5,RICTOR,RNF19A,S1PR1,SELENON,SLC11A2,SMARCA5,SMN1/SMN2,SSRP1,SSTR3,TERF1,TPP1,TRAPPC11,UBAP2L,UBE3A,YME1L1

Growth of organism	1.07E-03	1.815	39	ABCB1,AGO2,AIF1,APH1A,ARX,CSF1,EPN1,FANCD2,FOXO1,GNA13,GNAS,GPC3,GZMB,IGFBP4,IL15,IL6ST,LIMS1,NDUFS6,NF1,NR3C1,PDPK1,PGAP1,PLAT,POLB,PPP1CC,PRKCB,RAPGEF2,RBL1,S1PR1,SLC25A14,SLC4A10,SMARCA5,SMN1/SMN2,SNX27,ST6GAL1,STAT1,SYNJ1,TERF1,TFRC
Transitional-cell carcinoma	1.09E-03		13	ARHGAP35,CHD6,CTLA4,DHFR,FOXO1,GNA13,NF1,NLRC5,NR3C1,POLE,PRKCB,RRM1,STAG2
Activation of leukocytes	1.10E-03	0.172	33	CCR7,CD2,CD200,CSF1,CSF1R,CTLA4,CXCR5,DAPP1,FASLG,FCRL3,FOXO1,GJA4,HSPA4,IGHG1,IL15,KIR3DL1,NFATC3,NFKBID,NOD2,PIK3CD,PLAT,PLCG2,PRKCB,RAB4A,SATB1,SELL,SIRPG,ST6GAL1,STAT1,TAB2,TAB3,TIGIT,ZBTB46
Morphology of lymphoid organ	1.10E-03		26	CCR7,CD200,CSF1,CSF1R,CTLA4,CXCR5,FASLG,GPC3,IGHG1,IL15,IL6ST,NFATC3,NLRC5,NOD2,NR3C1,PDPK1,PIK3CD,RASSF2,RASSF5,RBL1,RBM38,SATB1,SELL,SNX27,TPP2,ZBTB46
Chronic inflammation	1.12E-03		5	IL6ST,NR3C1,PIK3CD,PLAT,STAT1
Cell death of cervical cancer cell lines	1.13E-03	-1.306	21	ABCB1,ATP2B1,BIRC2,BUB3,CLASP1,CSF1R,FASLG,GZMB,IKBKE,LIMS1,LYPLA1,NEDD9,NEK6,NR3C1,PDPK1,RICTOR,STAT1,TERF1,TRAP1,UBA7,VOPP1
Central nervous system cancer	1.17E-03		30	ARHGAP35,CD200,CLTB,CSF1,CSF1R,CTLA4,DHFR,DLEU1,ELK1,ELMO1,FOXO1,GJA4,GNAQ,IL15,MMP1,NEK6,NF1,NR3C1,PLAC8,POLB,POLE,PRKCB,PVT1,RAPGEF2,RRM1,SEC11A,STAG2,SYNJ1,TERF1,TLE3
Fibrosis of bone marrow	1.20E-03		6	CSF1R,DNMT3A,HMGA2,IGHG1,NF1,NR3C1
Formation of microtubules	1.20E-03	0.896	8	CLASP1,DYNLL1,GNA13,GZMB,NEK6,PDPK1,TERF1,UBAP2L
Cell death of osteosarcoma cells	1.21E-03	↓ -3.000	9	COPB2,EIF3G,FAU,PSMC6,PSMD12,RBM8A,RPA2,RPL19,SF3B3
Migration of lymphatic system cells	1.22E-03	1.678	21	CCR7,CD2,CTLA4,CXCL13,CXCR5,ELMO1,FASLG,FOXO1,GNA13,IL15,IL6ST,NR3C1,PDPK1,PIK3CD,PLCG2,RASSF5,RICTOR,S1PR1,SELL,STAT1,TXK
Abnormal morphology of red pulp	1.23E-03		4	CCR7,RASSF2,RASSF5,RBL1
NPM1 mutation positive acute myeloid leukemia	1.23E-03		4	NR3C1,POLB,POLE,RRM1
Quantity of effector T lymphocytes	1.23E-03		4	CTLA4,DNMT3A,IL15,STAT1
Hemorrhagic disease	1.24E-03	-0.440	16	ATP2C1,CXCL13,FASLG,GGCX,IL15,MCFD2,NR3C1,OAS2,PDPK1,PLAT,POLB,RBM8A,RPL18,S1PR1,TPP2,ZFPM1
Abnormal morphology of immune system	1.25E-03		17	ABCB1,CD200,CSF1,CSF1R,CTLA4,CXCR5,FASLG,FCMR,IGHG1,IL15,NOD2,PDPK1,PIK3CD,PLCG2,RASSF2,TPP2,ZBTB46
Development of lymph follicle	1.26E-03		5	CCR7,CTLA4,CXCR5,FASLG,RICTOR
Function of heart ventricle	1.26E-03	-1.131	5	ADRB2,CSF1,GNAQ,GNAS,NDUFS6
Osteoclastogenesis of macrophages	1.26E-03	-1.474	5	CSF1,FASLG,FOXO1,PLCG2,STAT1
Urothelial cancer	1.29E-03		12	ARHGAP35,CHD6,CTLA4,DHFR,FOXO1,NF1,NLRC5,POLE,PRKCB,RRM1,SEC11A,STAG2
Activation-induced cell death of lymphoma cell lines	1.30E-03		2	FASLG,SATB1
Acute otitis media	1.30E-03		2	DHFR,NR3C1
Cytokinesis of lung cancer cell lines	1.30E-03		2	PPP1CC,SSRP1
Development of bone marrow precursor cells	1.30E-03		2	CSF1,IL15
Egression of follicular B lymphocytes	1.30E-03		2	GNA13,S1PR1
Enlargement of embryonic tissue	1.30E-03		2	APAF1,EDA
Focal dystonia	1.30E-03		2	VAMP1,VAMP2
Hyperplasia of tunica intima	1.30E-03		2	AIF1,NF1
Migration of adenocarcinoma cells	1.30E-03		2	CSF1,CSF1R
Moderate asthma	1.30E-03		2	ADRB2,NR3C1
Myxoma	1.30E-03		2	GNAS,PRKAR1A
Polarization of CD4+ T-lymphocytes	1.30E-03		2	CCR7,ELMO1
Production of macrophages	1.30E-03		2	CSF1,CSF1R
Production of osteoclasts	1.30E-03		2	CSF1,CSF1R
Quantity of epithelial precursor cells	1.30E-03		2	CSF1,CSF1R
Quantity of neuroepithelial tumor	1.30E-03		2	FOXO1,RBL1
Quantity of peripheral lymph node	1.30E-03		2	CCR7,CXCR5
Recruitment of tumor-associated macrophages	1.30E-03		2	CSF1,CSF1R
Size of centrosome	1.30E-03		2	LDLRAP1,TPP2
Size of medullary cavity	1.30E-03		2	CSF1,GNAS
Hematopoiesis in embryo	1.32E-03		6	BRD1,RAPGEF2,RCOR1,TFRC,TNRC6C,ZFPM1

Ectopic ACTH secreting tumor	1.33E-03		3	GNAS,NR3C1,SSTR3
Giant cell arteritis	1.33E-03		3	DHFR,FDPS,NR3C1
Morphology of sebaceous glands	1.33E-03		3	CBX7,EDA,RBL1
Stimulation of cytotoxic T cells	1.33E-03		3	CTLA4,FASLG,IL15
Size of muscle	1.33E-03		10	ADRB2,DMPK,FOXO1,GNAQ,GNAS,IL6ST,LIMS1,PDPK1,SELENON,SMN1/SMN2
Relapsed lymphocytic leukemia	1.34E-03		7	CSF1R,DHFR,NR3C1,PIK3CD,POLB,POLE,RRM1
Metastatic breast cancer	1.36E-03		9	ABCB1,CCR7,DHFR,FDPS,NR3C1,PIK3CD,POLE,RRM1,STAT1
Brain metastasis	1.42E-03		5	CTLA4,DHFR,MMP1,NR3C1,POLB
Morphology of leukocytes	1.43E-03		18	ABCB1,CD200,CSF1,CSF1R,CTLA4,CXCR5,FASLG,FCMR,GNA13,IGHG1,IL15,NOD2,PDPK1,PIK3CD,PLCG2,RASSF2,TPP2,ZBTB46
Homing of T lymphocytes	1.44E-03	1.281	9	CCR7,CXCL13,CXCR5,ELMO1,FOXO1,IL15,NR3C1,S1PR1,SELL
Recurrent acute myeloid leukemia	1.44E-03		6	ABCB1,CSF1R,DHFR,POLB,POLE,RRM1
Colony formation of cervical cancer cell lines	1.45E-03	0	4	HMGA2,NEK6,NF1,TES
Migration of carcinoma cells	1.45E-03	0.747	4	CSF1,CSF1R,NEDD9,ST6GAL1
Colony formation	1.46E-03	1.368	28	CCR7,CKS1B,CSF1,CSF1R,DHFR,GPC3,GZMB,HDGF,HMGA2,IGFBP4,IL6ST,MAML2,NEK6,NF1,NR3C1,PDPK1,PPP2CA,PRKAR1A,PRKCB,RASSF2,RASSF5,RICTOR,RRM1,SATB1,SPRY1,ST6GAL1,TES,UBE3A
Aggregation of blood cells	1.48E-03	0.960	15	ADRB2,ATRN,ELK1,GJA4,GNA13,GNAQ,GNAS,IL15,MMP1,PDPK1,PIK3CD,PLAT,PLCG2,SELL,ZFPM1
Lymphocyte migration	1.49E-03	1.874	20	CCR7,CD2,CTLA4,CXCL13,CXCR5,ELMO1,FASLG,FOXO1,GNA13,IL15,IL6ST,NR3C1,PDPK1,PIK3CD,RASSF5,RICTOR,S1PR1,SELL,STAT1,TXK
Adhesion of blood cells	1.49E-03	1.134	21	ADRB2,ATRN,CCR7,CD2,CD200,CSF1,CXCL13,FASLG,FOXO1,GZMB,IL15,IL6ST,NEDD9,NR3C1,PIK3CD,PILRA,PLCG2,RASSF5,RICTOR,SELL,STAT1
Cell death of epithelial cells	1.53E-03	-1.057	26	AAK1,APAF1,ATP13A2,BIRC2,CSF1R,CUL4B,DYNLL1,ELK1,FASLG,FOXO1,IL15,NEK6,NR3C1,PDPK1,PLAT,PPP2CA,PRKCB,RASSF5,RBCK1,RBL1,S1PR1,SLC22A2,SSRP1,STAT1,TFRC,VOPP1
High bone mass disease	1.55E-03	1.176	7	CSF1,CSF1R,DHFR,FDPS,IGHG1,PLCG2,RRM1
Adult Philadelphia chromosome negative cortical T acute lymphoblastic leukemia	1.57E-03		6	CSF1R,DHFR,NR3C1,POLB,POLE,RRM1
Adult Philadelphia chromosome negative precursor T-cell acute lymphoblastic leukemia	1.57E-03		6	CSF1R,DHFR,NR3C1,POLB,POLE,RRM1
Adult Philadelphia chromosome negative pro-B-cell acute lymphoblastic leukemia	1.57E-03		6	CSF1R,DHFR,NR3C1,POLB,POLE,RRM1
ALK fusion negative large cell lymphoma	1.59E-03		5	DHFR,NR3C1,POLB,POLE,RRM1
Acute myeloid leukemia associated with myelodysplastic syndrome	1.59E-03		5	CSF1R,DHFR,DNMT3A,POLB,RRM1
Binding of B lymphocytes	1.59E-03	1.951	5	CXCL13,NFATC3,RASSF5,SELL,TFRC
Inflammation of peritoneum	1.59E-03	1.195	5	ADRB2,BIRC2,FASLG,SELL,ST6GAL1
Cytopenia	1.60E-03	-1.432	19	ABCB1,ADRB2,ATP2C1,CXCL13,DHFR,FASLG,IGHG1,IL15,NR3C1,OAS2,PDPK1,PIK3CD,PLAT,POLB,RBM8A,RRM1,S1PR1,TPP2,ZFPM1
Function of T lymphocytes	1.64E-03	-1.127	15	ABCB1,ADRB2,CCR7,CTLA4,IL15,NEDD9,NLRC5,NOD2,PIK3CD,S1PR1,SELL,SKP2,TCF7,TPP2,TXK
Immunodeficiency	1.64E-03		15	CSF1R,CTLA4,DHFR,DNMT3A,FASLG,PIK3CD,PLCG2,POLB,POLE,RBCK1,RRM1,SKIV2L,STAT1,TFRC,ZBTB24
Uptake of monosaccharide	1.65E-03	-0.159	16	ADRB2,CSF1,CSF1R,ELK1,GNAQ,GNAS,NOD2,PDPK1,PLAT,PLD1,PRKCB,RAB4A,SKP2,SLC27A1,SLC5A10,SNX27
Necrosis of muscle	1.69E-03	-0.530	19	ADRB2,APAF1,CSF1R,DMPK,FASLG,FOXO1,GNAQ,GNAS,IKBKE,IL6ST,LIMS1,PDPK1,PLAT,PLD1,PPP1CC,PRKCB,SMN1/SMN2,STAT1,WTAP
Differentiation of effector T lymphocytes	1.70E-03	-1.067	4	CTLA4,FOXO1,IL15,RICTOR
Refractory anemia with excess blasts in transformation	1.70E-03		4	DHFR,NR3C1,POLB,RRM1
Quantity of T lymphocytes	1.73E-03	0.755	27	ABCB1,CCR7,CSF1,CTLA4,DKC1,DNMT3A,FASLG,FCMR,FOXO1,IL15,IL6ST,NF1,NFATC3,NLRC5,NOD2,NR3C1,PIK3CD,POLR3E,RASSF2,RASSF5,RICTOR,SATB1,SELL,STAT1,TIGIT,TPP2,TXK
Abnormal morphology of antigen presenting cells	1.75E-03		3	CSF1R,CXCR5,PDPK1

CD274 negative urothelial carcinoma	1.75E-03		3	CTLA4,POLE,RRM1
CD274 positive urothelial carcinoma	1.75E-03		3	CTLA4,POLE,RRM1
Distribution of leukocytes	1.75E-03		3	CCR7,PIK3CD,S1PR1
Pancreatic intraductal papillary mucinous tumor	1.75E-03		3	GNAS,POLE,RRM1
Quantity of enteroendocrine cells	1.75E-03		3	ARX,CSF1,CSF1R
Stage IV non-Sezary/visceral disease	1.75E-03		3	DHFR,POLE,RRM1
Stage IV recurrent squamous cell lung cancer	1.75E-03		3	CTLA4,POLE,RRM1
Stage IV urethral cancer	1.75E-03		3	CTLA4,POLE,RRM1
Transitional cell carcinoma of the renal pelvis	1.75E-03		3	CTLA4,POLE,RRM1
Transcription of DNA	1.75E-03	1.476	64	ADRB2,AGO2,ARHGAP35,ARX,ATF7IP,BRMS1,BRWD1,CASK,CBX7,CKAP2,CKS1B,CREB3L2,DNMT3A,EIF4A3,ELK1,FASLG,FOXO1,GCFC2,GNAS,GTF3C2,HDGF,HMGA2,IRF9,KDM7A,LIMS1,MAML2,MBD1,METTL23,MLF1,NAB1,NEK4,NFATC3,NLRC5,NOD2,NONO,NR3C1,PARP9,PLAC8,PPP2CA,PREB,PRKAR1A,PRKCB,RBAK,RBCK1,RBL1,RCOR1,S1PR1,SATB1,SMARCA5,SNIP1,STAT1,TAF7,TCF7,THRAP3,TLE3,TNFSF8,TXK,UBE3A,UBN1,USP3,ZC3H8,ZFPM1,ZNF202,ZNF692
Colony formation of cells	1.77E-03	1.615	26	CKS1B,CSF1,CSF1R,DHFR,GPC3,GZMB,HDGF,HMGA2,IGFBP4,IL6ST,MAML2,NEK6,NF1,NR3C1,PDPK1,PPP2CA,PRKAR1A,PRKCB,RASSF2,RASSF5,RICTOR,RRM1,SATB1,SPRY1,ST6GAL1,TES
Philadelphia positive B-cell precursor acute lymphoblastic leukemia	1.77E-03		5	DHFR,NR3C1,POLB,POLE,RRM1
T-cell lymphoblastic lymphoma	1.77E-03		5	CD2,DHFR,NR3C1,POLB,POLE
Progression of malignant tumor	1.78E-03		12	CKS1B,CSF1R,CTLA4,DHFR,FOXO1,MMP1,NR3C1,PIK3CD,POLB,POLE,RRM1,SKP2
Aggregation of blood platelets	1.79E-03	0.788	13	ADRB2,ELK1,GJA4,GNA13,GNAQ,GNAS,MMP1,PDPK1,PIK3CD,PLAT,PLCG2,SELL,ZFPM1
Dysplasia	1.83E-03	-1.446	27	ABCB1,ADRB2,AGPS,APAF1,APH1A,ARIH1,B3GALT6,CREB3L2,CSF1,CSF1R,DHFR,DNMT3A,FANCD2,FASLG,FDPS,GNAS,IGHG1,MGA,NF1,NR3C1,POLB,POLE,PRKAR1A,RBL1,RRM1,STAG2,TLE3
Langerhans cell histiocytosis	1.86E-03		6	DHFR,MMP1,NR3C1,POLB,POLE,RRM1
Size of cardiac muscle	1.86E-03		6	ADRB2,FOXO1,GNAQ,IL6ST,LIMS1,PDPK1
Quantity of thymus gland	1.88E-03	↑ 2.020	14	ABCB1,CCR7,CTLA4,FOXO1,NOD2,NR3C1,PIK3CD,POLR3E,RASSF5,RICTOR,SATB1,STAT1,TPP2,TXK
Lymphoblastic lymphoma	1.91E-03		7	CD2,CSF1R,DHFR,NR3C1,POLB,POLE,RRM1
Benign oral disorder	1.97E-03		22	AKAP8,APAF1,CCR7,COX7B,CSF1,CXCL13,CXCR5,DHFR,EVC,FASLG,FDPS,IFITM1,IGHG1,LONP1,MATK,MMP1,NR3C1,PLAC8,POLE,STAT1,VAMP1,VAMP2
Philadelphia positive T-cell precursor acute lymphoblastic leukemia	1.97E-03		5	DHFR,NR3C1,POLB,POLE,RRM1
Recurrent leukemia	1.97E-03		8	ABCB1,CSF1R,DHFR,NR3C1,PIK3CD,POLB,POLE,RRM1
Size of muscle cells	1.98E-03		9	ADRB2,DMPK,FOXO1,GNAQ,GNAS,LIMS1,PDPK1,SELENON,SMN1/SMN2
Gastrointestinal stromal tumor	2.03E-03		6	ABCB1,CSF1R,DHFR,GLS,MTRR,NF1
Abnormal morphology of leukocytes	2.07E-03		13	ABCB1,CSF1,CSF1R,CTLA4,CXCR5,FASLG,FCMR,IGHG1,IL15,PDPK1,PIK3CD,RASSF2,TPP2
Acute leukemia	2.11E-03	0.152	56	ABCB1,ACOT11,ADRB2,AKAP17A,APH1A,ARHGAP35,ARIH1,ATP6V1A,BEST1,CD2,CD200,CLTB,CSF1R,DHFR,DNMT3A,ELMO1,ETFA,EVC,FAM86B2/FAM86KP,FANCD2,FASLG,GCC2,GLS,GNAS,IL15,ILKAP,IPO7,KLHL4,LIMS3/LIMS4,MAML2,MCM6,MLF1,MOCS1,MRPS5,NF1,NR3C1,PDE3B,PDE4DIP,PDPK1,PHF3,PHKB,PHYHD1,PIK3CD,POLB,POLE,PRR14L,RASA4,RRM1,SETD1B,SIRPG,SKP2,STAG2,TCF7,THRAP3,USP3,ZNF827
Arrest in proliferation of cells	2.15E-03		13	CBX7,GLS,IL6ST,IPO7,NR3C1,PPP1CC,PRKCB,RASSF5,RBL1,SKP2,SMARCA5,STAT1,TAF7
Interphase	2.16E-03	0.101	32	ABCB1,BABAM1,CACUL1,CKS1B,CSF1,CSF1R,CTLA4,DBF4,ELK1,FANCD2,FOXO1,GNL3,GNL3L,HMGA2,IL15,ILKAP,NAT10,NEK4,NEK6,NR3C1,PLAC8,POLB,POLE,PPP2CA,RBCK1,RBL1,RBM38,SKP2,STAT1,TERF1,TFRC,UBE3A
Congenital anomaly of skin	2.16E-03		12	ADRB2,ATP6V1A,B3GALT6,COX7B,DKC1,EDA,EVC,GNAQ,MMP1,NF1,NR3C1,RRM1
Vasculitis	2.19E-03	-1.964	7	CTLA4,DHFR,FDPS,IGHG1,IL15,NR3C1,PLAT
Antiviral response of cells	2.19E-03	1.000	5	BIRC2,CCR7,RICTOR,SPRY1,STAT1
Progression of squamous-cell carcinoma	2.19E-03		5	CKS1B,CTLA4,POLE,RRM1,SKP2
Hereditary bleeding disorder	2.20E-03		6	GGCX,MCFD2,NR3C1,PLAT,POLB,RBM8A

Shape change of tumor cell lines	2.20E-03	0.860	11	CHN1,CSF1,FASLG,GEM,GNA13,GNAQ,MATK,PLD1,PPFIA1,PRKCB,RBL1
Incidence of digestive organ tumor	2.21E-03	-1.970	8	CBX7,NF1,PRKAR1A,PRKCB,RASSF5,RBM38,STAT1,TES
Metastatic head and neck tumor	2.21E-03		8	CSF1R,CTLA4,DHFR,MMP1,NR3C1,POLB,POLE,RRM1
Cell death of lymphoma cell lines	2.23E-03	-1.080	13	FASLG,FOXO1,GLS,GNA13,IGFBP4,IGHG1,LONP1,PLCG2,PRKCB,SATB1,ST6GAL1,STAT1,TPP2
Activation-induced cell death of tumor cell lines	2.24E-03		3	FASLG,KIR3DL1,SATB1
Cell movement of naive T lymphocytes	2.24E-03		3	CCR7,CXCR5,SELL
Hemophilia A	2.24E-03		3	MCFD2,NR3C1,POLB
Large cell transformed mycosis fungoides	2.24E-03		3	DHFR,POLE,RRM1
Recurrent CD274 positive malignant solid tumor	2.24E-03		3	CTLA4,POLE,RRM1
Refractory Philadelphia-negative B-cell acute lymphoblastic leukemia	2.24E-03		3	POLB,POLE,RRM1
Relapsed Philadelphia-negative B-cell acute lymphoblastic leukemia	2.24E-03		3	POLB,POLE,RRM1
Stimulation of leukemia cell lines	2.24E-03		3	CD2,CSF1,FASLG
Angioimmunoblastic T-cell lymphoma	2.25E-03		10	CD200,CXCL13,DHFR,DNMT3A,GJA4,MMP1,NR3C1,POLB,POLE,RRM1
Adult acute myeloid leukemia with del(5q)	2.28E-03		4	ABCB1,DHFR,POLB,RRM1
Hepatosplenic T-cell lymphoma	2.28E-03		4	DHFR,NR3C1,POLE,RRM1
Refractory peripheral T-cell lymphoma	2.28E-03		4	DHFR,NR3C1,POLE,RRM1
Relapsed peripheral T-cell lymphoma	2.28E-03		4	DHFR,NR3C1,POLE,RRM1
T(15;17) mutation negative acute myeloid leukemia	2.28E-03		4	CSF1R,POLB,POLE,RRM1
Cell death of embryonic cell lines	2.29E-03	-1.004	20	AAK1,APAF1,ATP13A2,BIRC2,CUL4B,FASLG,FOXO1,GZMB,PDPK1,POLB,PRKCB,RASSF5,RBL1,SKP2,SLC22A2,SSRP1,STAT1,TFRC,VOPP1,YME1L1
Quantity of marginal-zone B lymphocytes	2.34E-03	1.746	7	FCMR,FOXO1,GNA13,NEDD9,NFATC3,PIK3CD,ST6GAL1
Recurrent acute leukemia	2.34E-03		7	ABCB1,CSF1R,DHFR,NR3C1,POLB,POLE,RRM1
Diffuse B-cell lymphoma	2.35E-03		26	AKAP8,CHD6,CRNN,CSF1R,DHFR,ELK1,FILIP1L,FOXO1,GNA13,LONP1,MBD1,MGA,NEK4,NF1,NR3C1,OSBP,PIK3CD,PLCG2,PLEKHG3,POLB,POLE,RBCK1,RRM1,SKIV2L2,SLC4A10,TNRC6C
Systemic CD30 positive anaplastic large cell lymphoma	2.38E-03		6	DHFR,NR3C1,POLB,POLE,RRM1,STAT1
Development of hematopoietic system	2.42E-03	0.097	18	AGO2,BRD1,CSF1,FASLG,FOXO1,HMGA2,IL15,IL6ST,NAB1,PLCG2,RBM38,RCOR1,RICTOR,SATB1,SMARCA5,STAT1,TCF7,ZFPM1
Classic Hodgkin disease	2.42E-03		5	FOXO1,NR3C1,POLB,POLE,RRM1
Extranodal marginal zone cell lymphoma	2.42E-03		5	CCR7,CXCL13,CXCR5,NR3C1,PIK3CD
Stage IV non-Hodgkin lymphoma	2.42E-03		5	DHFR,NR3C1,POLB,POLE,RRM1
Plasma cell dyscrasia	2.45E-03		23	ABCB1,ADRB2,ATRN,CBX7,CSF1R,DHFR,EPSTI1,FDPS,IL15,NONO,NR3C1,PIK3CD,PLCG2,PLEKHG2,POLE,PPP2CA,PPP4R2,PRKCB,RAB4A,RICTOR,RRM1,SATB1,TFRC
AIDS dementia complex	2.56E-03		2	GLS,POLB
Binding of glucocorticoid response element	2.56E-03		2	ADRB2,NR3C1
Cell division of myeloid cells	2.56E-03		2	CSF1,RBL1
Cervical dystonia	2.56E-03		2	VAMP1,VAMP2
Childhood asthma	2.56E-03		2	ADRB2,NR3C1
Diffuse giant cell tumor of tendon sheath	2.56E-03		2	CSF1,CSF1R

Elongation of cervical cancer cell lines	2.56E-03		2	ELMO1,PLD1
Exercise induced asthma	2.56E-03		2	ADRB2,NR3C1
Experimental polyglutamine disease	2.56E-03		2	VAMP1,VAMP2
Inflammatory hepatocellular adenoma	2.56E-03		2	GNAS,IL6ST
Measles	2.56E-03		2	DHFR,FASLG
Refractory Philadelphia chromosome negative precursor T-cell acute lymphoblastic leukemia	2.56E-03		2	POLB,POLE
Relapsed Philadelphia chromosome negative precursor T-cell acute lymphoblastic leukemia	2.56E-03		2	POLB,POLE
Relapsed T-cell lymphoblastic lymphoma	2.56E-03		2	POLB,POLE
Ruffling of macrophage cancer cell lines	2.56E-03		2	CHN1,CSF1
Size of sebaceous glands	2.56E-03		2	CBX7,EDA
Status asthmaticus	2.56E-03		2	ADRB2,NR3C1
Cell movement of blood cells	2.56E-03	1.684	41	ADAM8,ADRB2,AIF1,ATRN,CCR7,CD2,CD200,CD302,CSF1,CSF1R,CTLA4,CXCL13,CXCR5,ELMO1,FASLG,FOXO1,GNA13,GNAS,GPC3,GZMB,IGHG1,IKBKE,IL15,IL6ST,MMP1,NEDD9,NOD2,NR3C1,PDPK1,PIK3CD,PLAT,PLCG2,PRKCB,RASSF5,RICTOR,S1PR1,SELL,STAT1,TNFSF8,TXK,VNN2
Replication of Influenza virus	2.59E-03	1.337	16	ATP2C1,ATP6V1A,COPB2,EIF3G,EIF4A3,FASLG,FAU,FDPS,IFITM1,IKBKE,LONP1,NLRC5,STAT1,TRIM14,UBA7,VNN2
Metabolism of protein	2.61E-03	-1.642	42	AAK1,AGO2,APAF1,APH1A,ARIH1,ATP13A2,COG3,CSF1,CTLA4,CUL4B,DHFR,DKC1,EDEM1,EEF2,EIF3G,EIF4A3,EIF4EBP2,ERP44,FBXW2,FOXO1,GNAQ,GNL3L,GPC3,GZMB,LONP1,MMP1,NR3C1,PASK,PDPK1,PLAT,PPP2CA,RASSF2,RBL1,RPL19,SF3B3,SKP2,SMN1/SMN2,SNX3,TPP1,TPP2,UBE3A,USP3
Adult acute myeloid leukemia with 11q23 abnormalities	2.61E-03		4	ABCB1,DHFR,POLB,RRM1
Adult acute myeloid leukemia with inv(16)(p13.1q22)	2.61E-03		4	ABCB1,DHFR,POLB,RRM1
Adult t(16;16)(p13;q22) acute myeloid leukemia	2.61E-03		4	ABCB1,DHFR,POLB,RRM1
Cell movement of memory T lymphocytes	2.61E-03	0	4	CCR7,CXCL13,IL15,S1PR1
Osteoclastogenesis of bone marrow-derived macrophages	2.61E-03	-1.188	4	CSF1,FASLG,FOXO1,PLCG2
Response of CD4+ T-lymphocytes	2.61E-03		4	CTLA4,GNAS,IL15,TIGIT
Cell viability of phagocytes	2.62E-03	0.668	8	CCR7,CSF1,CSF1R,FOXO1,IL15,NF1,PIK3CD,PLAT
Function of antigen presenting cells	2.62E-03		14	CCR7,CD200,CSF1,CSF1R,IL15,NOD2,PIK3CD,PLAT,PLCG2,RASSF5,SELL,ST6GAL1,STAT1,TIGIT
Uveitis	2.66E-03		7	ADRB2,CD200,CTLA4,DHFR,IL15,NR3C1,STAT1
Hereditary connective tissue disorder	2.72E-03	0.588	21	ADRB2,AGPS,ATP6V1A,B3GALT6,CREB3L2,CSF1,CSF1R,DHFR,DMPK,FDPS,GGCX,GNAS,IGHG1,MMP1,NF1,NR3C1,PLCG2,PRKAR1A,RRM1,SKIV2L,TLE3
Abnormal bone density	2.74E-03	0.697	13	ATP2C1,CSF1,CSF1R,CTLA4,DHFR,FDPS,GNAQ,IGHG1,NR3C1,PDE3B,PLCG2,RASSF2,RRM1
Inflammatory response	2.75E-03	1.392	36	ABCB1,ACOT11,ADAM8,ADRB2,AIF1,BIRC2,CCR7,CD200,CSF1,CSF1R,CXCL13,CXCR5,ELMO1,FANCD2,FASLG,GNA13,GNAS,IGHG1,IL15,IL6ST,MMP1,NEDD9,NFATC3,NOD2,NR3C1,PIK3CD,PLAT,PLCG2,POLB,PRKCB,RBL1,RICTOR,S1PR1,SELL,STAT1,ZBP1
Osteopetrosis	2.77E-03	1.176	6	CSF1,CSF1R,DHFR,IGHG1,PLCG2,RRM1
Apoptosis of muscle cells	2.79E-03	-0.657	15	ADRB2,APAF1,CSF1R,DMPK,FASLG,FOXO1,GNAQ,GNAS,IKBKE,IL6ST,LIMS1,PLD1,PPP1CC,STAT1,WTAP
Cell movement of T lymphocytes	2.79E-03	1.145	14	CCR7,CTLA4,CXCL13,CXCR5,ELMO1,FASLG,FOXO1,IL15,IL6ST,NR3C1,PIK3CD,S1PR1,SELL,STAT1
Pediatric neoplasm	2.80E-03		9	ABCB1,ADRB2,CSF1R,CTLA4,DHFR,NR3C1,POLB,POLE,RRM1
Benign salivary gland disease	2.80E-03		3	CXCL13,VAMP1,VAMP2
Cell flattening of tumor cell lines	2.80E-03		3	GEM,PLD1,RBL1
Egression of lymphocytes	2.80E-03		3	CCR7,GNA13,S1PR1
Hypertrophy of mesangial cells	2.80E-03		3	FOXO1,NFATC3,PDPK1
Morphology of adrenal gland cells	2.80E-03		3	NR3C1,PDPK1,VAMP2

Oncogene-induced senescence of cells	2.80E-03		3	FANCD2,HMGA2,PDPK1
Recurrent prostatic carcinoma	2.80E-03		3	NR3C1,POLE,RRM1
Stage IV ALK translocation negative CD274 positive non-small cell lung cancer	2.80E-03		3	DHFR,POLE,RRM1
Adhesion of mononuclear leukocytes	2.80E-03	1.821	10	CCR7,CD2,FOXO1,IL15,IL6ST,NEDD9,NR3C1,RASSF5,RICTOR,SELL
Hodgkin disease	2.83E-03		7	DHFR,FOXO1,NR3C1,POLB,POLE,RRM1,TNFSF8
Estrogen receptor negative breast tumor	2.91E-03		11	ABCB1,AGPS,CSF1R,DHFR,FDPS,GNAS,PLAT,POLE,RRM1,STAT1,UBE3A
Embolus	2.93E-03		5	GGCX,GNAQ,PLAT,POLE,RRM1
Recurrent primary neoplasia	2.93E-03		5	DHFR,NR3C1,POLB,POLE,RRM1
Demyelination of spinal cord	2.98E-03		4	FASLG,NR3C1,PLAT,S1PR1
Shortening of telomeres	2.98E-03		4	GNL3L,HMGA2,NAT10,TERF1
Progesterone receptor negative breast tumor	3.03E-03		11	ABCB1,AGPS,CSF1R,DHFR,FDPS,GNAS,PLAT,POLE,RRM1,STAT1,UBE3A
Resorption of bone	3.03E-03	-1.420	11	ADAM8,ADRB2,AGO2,CSF1,CSF1R,DYNLL1,NF1,S1PR1,STAT1,TFRC,TPP1
Interaction of T lymphocytes	3.18E-03	1.878	10	ATRN,CCR7,CD2,CTLA4,FASLG,NR3C1,RASSF5,RICTOR,SELL,TXK
E2A-PBX1 positive B-cell acute lymphoblastic leukemia	3.22E-03		5	DHFR,NR3C1,POLB,POLE,RRM1
Meningeal neoplasm	3.22E-03		5	CSF1R,DHFR,GNAQ,NR3C1,POLB
Cardiac lesion	3.24E-03	-1.411	12	ADRB2,CSF1,GNAS,IKBKE,LIMS1,NDUFS6,NF1,PLAT,PNKD,PRKAR1A,PRKCB,STAT1
Accumulation of T lymphocytes	3.25E-03	0.443	8	CCR7,CTLA4,FASLG,IL15,IL6ST,NR3C1,S1PR1,SELL
Cell death of carcinoma cell lines	3.29E-03	-0.809	17	ADI1,APAF1,BIRC2,FASLG,GPC3,IKBKE,IL6ST,ILKAP,NR3C1,PPP2CA,PRKAR1A,RICTOR,RPA2,RRM1,SKP2,STAT1,TRAP1
Chronic lymphocytic leukemia	3.37E-03		22	AKAP8,CD200,CEP68,DLEU1,FASLG,FCMR,LONP1,LYPLA1,NEDD9,NR3C1,PDE3B,PIK3CD,PLCG2,PNKD,POLB,POLE,PRKCB,RASSF5,RRM1,S1PR1,TIGIT,UCKL1
Immune response of T lymphocytes	3.38E-03	-1.044	9	CCR7,CTLA4,FASLG,FOXO1,GNAS,HSDL1,IL15,TIGIT,TNFSF8
Mantle cell lymphoma	3.38E-03		9	DHFR,LONP1,NEK4,NR3C1,PLCG2,PLEKHG3,POLB,POLE,RRM1
Weakness	3.38E-03		9	CNTN1,DMPK,GNAS,NAB1,NFATC3,NR3C1,SMN1/SMN2,SYNJ1,TRAPPC11
Differentiation of osteoclast precursor cells	3.38E-03	-1.114	4	CD200,CSF1,CSF1R,DYNLL1
Hypoplasia of lymph node	3.38E-03	-1.964	4	CTLA4,NEDD9,NFATC3,SELL
Stage IV ALK fusion negative CD274 negative non-small cell lung cancer	3.38E-03		4	CTLA4,DHFR,POLE,RRM1
Stage IV ALK fusion negative CD274 positive non-small cell lung cancer	3.38E-03		4	CTLA4,DHFR,POLE,RRM1
Stage IV EGFR mutation negative CD274 positive non-small cell lung cancer	3.38E-03		4	CTLA4,DHFR,POLE,RRM1
I-kappaB kinase/NF-kappaB cascade	3.40E-03	1.237	11	ATP2C1,NDFIP2,NEK6,NFKBID,NOD2,PDPK1,PRKCB,RBCK1,SNIP1,STAT1,TRIM5
T cell migration	3.43E-03	1.402	15	CCR7,CTLA4,CXCL13,CXCR5,ELMO1,FASLG,FOXO1,IL15,IL6ST,NR3C1,PIK3CD,S1PR1,SELL,STAT1,TXK
Chronic inflammatory disorder	3.44E-03	-0.359	48	ABCB1,ADAM8,ADRB2,AIF1,ARIH1,ATP2B1,ATP2C1,BRMS1,BRWD1,C5orf56,CCR7,CDKAL1,CSF1,CTLA4,CXCL13,CXCR5,DHFR,DYNLL1,EEF2,EVC,FASLG,FAU,FCRL3,FDPS,FOXO1,GZMB,IGFBP4,IGHG1,IL15,IL6ST,MBD1,MMP1,NOD2,NONO,NR3C1,PDE3B,PIK3CD,PLD1,POLB,RBM38,RPL19,S1PR1,SKIV2L,STAT1,TFRC,TLE3,TNFSF8,VNN2
Dermatitis	3.44E-03	0.489	22	ARIH1,CCR7,CD2,CXCL13,DHFR,FASLG,GZMB,IGHG1,IL15,IRF9,MMP1,NR3C1,PIK3CD,PLCG2,POLE,PRKCB,RBCK1,RRM1,SELL,STAT1,TNFSF8,TXK
Cell viability of monocyte-derived dendritic cells	3.45E-03		3	CCR7,FOXO1,PIK3CD
Erythropoiesis of hematopoietic cells	3.45E-03		3	BRD1,SMARCA5,ZFPM1
Length of plasma membrane projections	3.45E-03		3	ARHGAP35,ARX,VAMP2
Mass of interscapular fat pad	3.45E-03		3	ADRB2,GNAS,RBL1

Migration of memory T lymphocytes	3.45E-03		3	CCR7,CXCL13,S1PR1
Progressive primary central nervous system lymphoma	3.45E-03		3	DHFR,NR3C1,POLB
Recurrent primary central nervous system lymphoma	3.45E-03		3	DHFR,NR3C1,POLB
Childhood solid tumor	3.46E-03		6	ADRB2,CTLA4,DHFR,NR3C1,POLB,RRM1
High risk acute lymphocytic leukemia	3.46E-03		6	CSF1R,DHFR,NR3C1,POLB,POLE,RRM1
Methylation of DNA	3.46E-03		6	ATF7IP,DNMT3A,GNAS,MTRR,PPP2CA,RBL1
Refractory non-Hodgkin lymphoma	3.46E-03		6	DHFR,NR3C1,PIK3CD,POLB,POLE,RRM1
Selection of lymphocytes	3.46E-03	1.936	6	CCR7,ELK1,IL6ST,NF1,TNFSF8,TXK
Stage I-IV nonsquamous non-small cell lung carcinoma	3.46E-03		6	AGO2,CTLA4,DHFR,NR3C1,POLE,RRM1
Uptake of D-hexose	3.46E-03	0.140	13	ADRB2,CSF1,CSF1R,ELK1,GNAS,NOD2,PDPK1,PLAT,PLD1,SKP2,SLC27A1,SLC5A10,SNX27
Depletion of lymphatic system cells	3.53E-03	0.372	5	CSF1,CTLA4,FASLG,IL15,SELL
Recurrent central nervous system tumor	3.53E-03		5	CSF1R,CTLA4,DHFR,NR3C1,POLB
Cell death of kidney cell lines	3.56E-03	-1.452	17	AAK1,ATP13A2,BIRC2,CD2,CUL4B,FASLG,FOXO1,GLS,GNA13,GNAQ,PDPK1,PRKCB,RASSF5,SLC22A2,SSRP1,TFRC,VOPP1
Mitosis	3.66E-03	1.144	25	AKAP8,APAF1,BUB3,CKAP2,CLASP1,CSF1,CSF1R,DBF4,DYNC1LI2,GEM,GNA13,GNL3,IGHG1,MATK,NEK4,NEK6,PDS5A,PPP2CA,PRKCB,S1PR1,SKP2,STAT1,TERF1,UBA7,USP3
Leukocyte migration	3.68E-03	1.421	40	ADAM8,ADRB2,AIF1,ATRN,CCR7,CD2,CD200,CD302,CSF1,CSF1R,CTLA4,CXCL13,CXCR5,ELMO1,FASLG,FOXO1,GNA13,GNAS,GPC3,IGHG1,IKBKE,IL15,IL6ST,MMP1,NEDD9,NOD2,NR3C1,PDPK1,PIK3CD,PLAT,PLCG2,PRKCB,RASSF5,RICTOR,S1PR1,SELL,STAT1,TNFSF8,TXK,VNN2
Thoracic neoplasm	3.70E-03	-0.854	66	ABCB1,ADAM8,AGO2,APH1A,ARHGAP35,ATF7IP,CBX7,CHD6,CKS1B,COPB2,CSF1,CSF1R,CTLA4,CUL4B,CXCL13,DHFR,DNMT3A,DSCAML1,FANCD2,FASLG,FDPS,GEM,GLS,GNAQ,GNAS,HMGA2,IGHG1,IKBKE,IL15,IL6ST,KIR3DL1,KLHL4,MGA,MMP1,NAB1,NEDD9,NEK6,NF1,NR3C1,PASK,PDPK1,PHF3,PIFO,PLAT,POLB,POLE,PRKAR1A,PRKCB,PRR14L,RABGAP1L,RASSF5,RBL1,RP9,RRM1,S1PR1,SEC24C,SKP2,SSTR3,STAG2,STAT1,TARS2,TERF1,TLE3,UBAP2L,UBN1,ZZZ3
Apoptosis of B lymphocytes	3.70E-03	-1.149	9	CSF1,FASLG,FCMR,GZMB,IKBKE,PIK3CD,PLCG2,PRKCB,S1PR1
Interstitial lung disease	3.70E-03		9	AGO2,CSF1R,DHFR,ELK1,FASLG,HMGA2,NFATC3,NR3C1,PLAT
Mobilization of Ca2+	3.73E-03	0.668	18	ADRB2,ATP2B1,BRMS1,CCR7,CD2,CXCL13,CXCR5,GNA13,GNAQ,GNAS,IGHG1,NFATC3,PIK3CD,PILRA,PLCG2,RICTOR,SELL,TXK
Remodeling of bone	3.73E-03	-1.420	12	ADAM8,ADRB2,AGO2,CSF1,CSF1R,DYNLL1,NF1,RASSF2,S1PR1,STAT1,TFRC,TPP1
Pituitary lesion	3.76E-03	-1.964	10	CTLA4,DHFR,DKC1,GNAS,HMGA2,NF1,NR3C1,RBL1,SKP2,SSTR3
Cell viability of macrophages	3.82E-03	-0.110	4	CSF1,CSF1R,IL15,PLAT
Chemotaxis of natural killer cells	3.82E-03	1.974	4	GNA13,GNAS,PIK3CD,S1PR1
Proliferation of mast cells	3.82E-03	-0.762	4	IL15,IL6ST,NF1,PIK3CD
Refractory acute lymphocytic leukemia, type L3	3.82E-03		4	DHFR,POLB,POLE,RRM1
Relapsed classical Hodgkin lymphoma	3.82E-03		4	NR3C1,POLB,POLE,RRM1
Secondary myelodysplastic syndrome	3.82E-03		4	DHFR,NR3C1,POLB,RRM1
Stage IV bladder cancer	3.82E-03		4	CTLA4,DHFR,POLE,RRM1
Swelling of joint	3.82E-03		4	CCR7,CXCR5,IL6ST,PLAT
Accumulation of antigen presenting cells	3.83E-03	0.937	7	CCR7,CD200,CSF1,FASLG,IGHG1,PLAT,STAT1
Polycystic ovary syndrome	3.83E-03		7	BUB3,DHFR,IGFBP4,NEK4,NR3C1,PRKAR1A,TLE3
Progression of carcinoma	3.87E-03		9	CKS1B,CSF1R,CTLA4,DHFR,FOXO1,NR3C1,POLE,RRM1,SKP2
Replication of virus	3.95E-03	1.415	26	AGFG1,AGO2,ATP2C1,ATP6V1A,CD200,COPB2,EIF3G,EIF4A3,EIF4EBP2,FASLG,FAU,FDPS,IFITM1,IKBKE,IL15,IRF9,LONP1,NLRC5,PLD1,SSRP1,STAT1,TRIM14,TRIM5,UBA7,VNN2,ZBP1
Development of macrophages	3.98E-03	-1.482	6	CSF1,CSF1R,FASLG,FOXO1,PLCG2,STAT1
Shape change of blood cells	3.99E-03	0.201	8	ATRN,CCR7,CSF1,GNA13,GZMB,PDPK1,PLCG2,SELL
Relapsed acute leukemia	4.06E-03		7	ABCB1,CSF1R,DHFR,NR3C1,POLB,POLE,RRM1
Apoptosis of thymoma cell lines	4.18E-03		3	FASLG,FAU,NR3C1
Bullous pemphigoid	4.18E-03		3	DHFR,NR3C1,PLAT

Cellular infiltration by CD8+ T lymphocyte	4.18E-03		3	CTLA4,IL15,STAT1
Development of invariant natural killer T cells	4.18E-03		3	CCR7,RICTOR,TXK
Response of CD8+ T lymphocyte	4.18E-03		3	FOXO1,HSDL1,IL15
Cell movement of leukocytes	4.20E-03	↑ 2.083	36	ADAM8,ADRB2,AIF1,ATRN,CCR7,CD2,CD302,CSF1,CSF1R,CTLA4,CXCL13,CXCR5,ELMO1,FASLG,FOXO1,GNA13,GNAS,IGHG1,IKBKE,IL15,IL6ST,NEDD9,NOD2,NR3C1,PDPK1,PIK3CD,PLAT,PLCG2,PRKCB,RASSF5,RICTOR,S1PR1,SELL,STAT1,TNFSF8,TXK
Apoptosis of neural tube cells	4.20E-03		2	ATP2C1,FOXO1
Arrest in development of B lymphocytes	4.20E-03		2	FOXO1,PLCG2
Cell death of peripheral blood monocytes	4.20E-03		2	CSF1,FASLG
Colony formation of liver cells	4.20E-03		2	CSF1,CSF1R
Cytotoxic reaction of kidney cell lines	4.20E-03		2	ABCB1,SLC22A2
Fibrous dysplasia	4.20E-03		2	FDPS,GNAS
Formation of mesenteric lymph node	4.20E-03		2	CCR7,CXCR5
Humoral hypercalcemia of malignancy	4.20E-03		2	FDPS,NR3C1
Invasion of neuroglia	4.20E-03		2	CSF1,NF1
Lack of Langerhans cells	4.20E-03		2	CSF1R,PDPK1
Localization of green fluorescent protein	4.20E-03		2	SATB1,TFRC
Migration of osteoclast precursor cells	4.20E-03		2	CSF1,FOXO1
Movement of lymphoma cells	4.20E-03		2	CXCL13,PARP9
Phosphorylation of phosphatidylinositol-3,4,5-triphosphate	4.20E-03		2	PIK3CD,SYNJ1
Relaxation of ventricular myocytes	4.20E-03		2	ADRB2,SLC27A1
Size of lung carcinoma	4.20E-03		2	IL6ST,MMP1
Steroidogenesis of Leydig cells	4.20E-03		2	CSF1,IGFBP4
Transformation of gonadal cell lines	4.20E-03		2	DHFR,NR3C1
ALK fusion negative T-cell lymphoma	4.30E-03		4	DHFR,NR3C1,POLE,RRM1
Pemphigus	4.30E-03		4	CTLA4,DHFR,NR3C1,PLAT

[a] Absence of a Z-score entry indicates that Z-score cannot be calculated by IPA.

Table S6. Ingenuity Canonical Pathways enriched among DEGs in SG CD4+CD45RA- T cells of pSS cases versus nSS controls

Canonical Pathway	p-value	Z-score[a]	Pathway Molecules Present in DEG Dataset
B Cell Receptor Signaling	1.32E-04	1.732	NFATC3,PDPK1,IKBKE,IGHG1,NFKBID,DAPP1,SYNJ1,FOXO1,PLCG2,PIK3CD,RASSF5,ELK1,PRKCB
PI3K Signaling in B Lymphocytes	1.35E-03	↑ 2.333	NFKBID,DAPP1,NFATC3,PLCG2,PDPK1,IKBKE,PIK3CD,ELK1,PRKCB
Molecular Mechanisms of Cancer	1.48E-03		GNAS,TAB2,APAF1,GNAQ,RBL1,NFKBID,FOXO1,NF1,FANCD2,APH1A,ARHGEF18,PIK3CD,GNA13,ELK1,FASLG,PRKCB,BIRC2,PRKAR1A
Role of PKR in Interferon Induction and Antiviral Response	1.58E-03		NFKBID,TAB2,APAF1,IKBKE,STAT1
Prolactin Signaling	1.78E-03	1.633	PLCG2,PDPK1,PIK3CD,STAT1,TCF7,NR3C1,PRKCB
Gαq Signaling	1.95E-03	1.667	NFKBID,GNAS,NFATC3,PLCG2,GNAQ,IKBKE,PIK3CD,ELK1,PLD1,PRKCB
G-Protein Coupled Receptor Signaling	2.00E-03		GNAS,SSTR3,GNAQ,PDPK1,IKBKE,NFKBID,FFAR3,PDE3B,GDE1,S1PR1,PIK3CD,ADRB2,PRKAR1A,PRKCB
Small Cell Lung Cancer Signaling	2.04E-03		NFKBID,CKS1B,APAF1,IKBKE,PIK3CD,SKP2,BIRC2
G Beta Gamma Signaling	2.51E-03	1.890	GNAS,PLCG2,GNAQ,PDPK1,GNA13,PRKAR1A,PRKCB
Lymphotoxin β Receptor Signaling	2.75E-03	↑ 2.000	NFKBID,APAF1,PDPK1,IKBKE,PIK3CD,BIRC2
IL-1 Signaling	3.24E-03	↑ 2.646	NFKBID,GNAS,TAB2,GNAQ,IKBKE,GNA13,PRKAR1A
PI3K/AKT Signaling	4.17E-03	0.816	NFKBID,FOXO1,SYNJ1,PPP2CA,LIMS1,PDPK1,IKBKE,PIK3CD
Relaxin Signaling	5.25E-03	1.342	NFKBID,GNAS,PDE3B,GDE1,GNAQ,PIK3CD,GNA13,ELK1,PRKAR1A
Oncostatin M Signaling	5.37E-03	1.000	IL6ST,ELK1,STAT1,MMP1
TWEAK Signaling	5.37E-03	0	NFKBID,APAF1,IKBKE,BIRC2
p70S6K Signaling	6.46E-03	0.707	PPP2CA,EEF2,PLCG2,GNAQ,PDPK1,PIK3CD,PLD1,PRKCB
Erythropoietin Signaling	7.08E-03		NFKBID,PLCG2,PDPK1,PIK3CD,ELK1,PRKCB
Activation of IRF by Cytosolic Pattern Recognition Receptors	7.24E-03	0.447	NFKBID,ZBP1,IKBKE,IRF9,STAT1
Tec Kinase Signaling	7.59E-03	1.633	TXK,GNAS,PLCG2,GNAQ,PIK3CD,GNA13,STAT1,FASLG,PRKCB
Corticotropin Releasing Hormone Signaling	7.76E-03	1.633	GNAS,PLCG2,GNAQ,ELK1,FASLG,PRKAR1A,PRKCB
Insulin Receptor Signaling	7.94E-03	0	PPP1CC,FOXO1,SYNJ1,PDE3B,PDPK1,PIK3CD,VAMP2,PRKAR1A
Induction of Apoptosis by HIV1	8.32E-03	-0.447	NFKBID,APAF1,IKBKE,FASLG,BIRC2
Growth Hormone Signaling	8.32E-03	1.633	PLCG2,PDPK1,PIK3CD,ELK1,STAT1,PRKCB
April Mediated Signaling	8.71E-03	1.000	NFKBID,NFATC3,IKBKE,ELK1
α-Adrenergic Signaling	8.91E-03	0.816	PHKB,GNAS,PLCG2,GNAQ,PRKAR1A,PRKCB
GPCR-Mediated Nutrient Sensing in Enteroendocrine Cells	8.91E-03		FFAR3,GNAS,PLCG2,GNAQ,PRKAR1A,PRKCB
Cardiac β-adrenergic Signaling	9.12E-03	0.816	PPP1CC,GNAS,AKAP8,PPP2CA,PDE3B,GDE1,PRKAR1A,AKAP17A
IL-7 Signaling Pathway	9.33E-03	↑ 2.000	FOXO1,PDPK1,PIK3CD,IGHG1,CXCR5,STAT1
PEDF Signaling	1.00E-02	1.342	NFKBID,IKBKE,PIK3CD,ELK1,TCF7,FASLG
B Cell Activating Factor Signaling	1.05E-02		NFKBID,NFATC3,IKBKE,ELK1
Apoptosis Signaling	1.10E-02	0	NFKBID,PLCG2,APAF1,IKBKE,FASLG,BIRC2
PDGF Signaling	1.15E-02	1.633	SYNJ1,PLCG2,PIK3CD,ELK1,STAT1,PRKCB
Role of NFAT in Regulation of the Immune Response	1.17E-02	1.890	NFKBID,GNAS,NFATC3,PLCG2,CSNK1G3,GNAQ,IKBKE,PIK3CD,GNA13
Death Receptor Signaling	1.29E-02	0	NFKBID,APAF1,IKBKE,PARP9,FASLG,BIRC2
Tumoricidal Function of Hepatic Natural Killer Cells	1.35E-02		GZMB,APAF1,FASLG
Renin-Angiotensin Signaling	1.38E-02	1.633	PLCG2,GNAQ,PIK3CD,ELK1,STAT1,PRKAR1A,PRKCB
Regulation of eIF4 and p70S6K Signaling	1.45E-02		EIF3G,EIF4EBP2,PPP2CA,EIF4A3,AGO2,PDPK1,PIK3CD,FAU
Protein Kinase A Signaling	1.55E-02	↑ 2.309	GNAS,AKAP8,NFATC3,GNAQ,AKAP17A,NFKBID,PPP1CC,PHKB,PDE3B,GDE1,PLCG2,GNA13,ELK1,PRKCB,PRKAR1A
ErbB4 Signaling	1.58E-02	1.000	PLCG2,APH1A,PDPK1,PIK3CD,PRKCB
ErbB Signaling	1.62E-02		FOXO1,PLCG2,PDPK1,PIK3CD,ELK1,PRKCB
Cytotoxic T Lymphocyte-mediated Apoptosis of Target Cells	1.66E-02		GZMB,APAF1,FASLG
IL-15 Production	1.66E-02		TXK,IL15,STAT1
GPCR-Mediated Integration of Enteroendocrine Signaling Exemplified by an L Cell	1.74E-02		GNAS,PLCG2,GNAQ,PRKAR1A,ADRB2
Calcium Transport I	1.78E-02		ATP2B1,ATP2C1
TNFR1 Signaling	1.78E-02	1.000	NFKBID,APAF1,IKBKE,BIRC2
RANK Signaling in Osteoclasts	1.86E-02	↑ 2.236	NFKBID,TAB2,IKBKE,PIK3CD,ELK1,BIRC2
Dopamine-DARPP32 Feedback in cAMP Signaling	2.00E-02	1.414	PPP1CC,GNAS,PPP2CA,PLCG2,CSNK1G3,GNAQ,PRKAR1A,PRKCB
cAMP-mediated signaling	2.00E-02	0	FFAR3,GNAS,AKAP8,PDE3B,GDE1,SSTR3,S1PR1,PRKAR1A,AKAP17A,ADRB2
Type I Diabetes Mellitus Signaling	2.04E-02	0.816	NFKBID,GZMB,APAF1,IKBKE,STAT1,FASLG
Methylthiopropionate Biosynthesis	2.09E-02		ADI1
mTOR Signaling	2.14E-02	0.816	EIF3G,PPP2CA,EIF4A3,PDPK1,PIK3CD,RICTOR,PLD1,PRKCB,FAU
TNFR2 Signaling	2.24E-02		NFKBID,IKBKE,BIRC2
Role of Osteoblasts, Osteoclasts and Chondrocytes in Rheumatoid Arthritis	2.24E-02		NFKBID,FOXO1,CSF1,NFATC3,TAB2,IKBKE,PIK3CD,CSF1R,MMP1,BIRC2
Regulation of IL-2 Expression in Activated and Anergic T Lymphocytes	2.29E-02		NFKBID,NFATC3,PLCG2,IKBKE,ELK1
IGF-1 Signaling	2.34E-02	↑ 2.000	IGFBP4,FOXO1,PDPK1,PIK3CD,ELK1,PRKAR1A
eNOS Signaling	2.34E-02	1.414	HSPA4,GNAS,PLCG2,GNAQ,PDPK1,PIK3CD,PRKAR1A,PRKCB

Docosahexaenoic Acid (DHA) Signaling	2.34E-02		FOXO1,APAF1,PDPK1,PIK3CD
T Cell Receptor Signaling	2.40E-02		TXK,NFATC3,IKBKE,PIK3CD,ELK1,CTLA4
Role of Macrophages, Fibroblasts and Endothelial Cells in Rheumatoid Arthritis	2.51E-02		IL6ST,NFKBID,CSF1,NFATC3,PLCG2,IL15,GNAQ,IKBKE,PIK3CD,IGHG1,MMP1,PRKCB
Hematopoiesis from Multipotent Stem Cells	2.51E-02		CSF1,IL15
BER pathway	2.51E-02		POLE,POLB
PPARα/RXRα Activation	2.57E-02	0.447	NFKBID,GNAS,PLCG2,GNAQ,IKBKE,SLC27A1,PRKAR1A,PRKCB
Telomerase Signaling	2.63E-02	1.000	PPP2CA,DKC1,TPP1,PDPK1,PIK3CD,TERF1
G Protein Signaling Mediated by Tubby	2.69E-02		GNAS,PLCG2,GNAQ
Androgen Signaling	2.75E-02		HSPA4,GNAS,GNAQ,GNA13,PRKAR1A,PRKCB
Macropinocytosis Signaling	2.75E-02	0.447	CSF1,PLCG2,PIK3CD,CSF1R,PRKCB
Breast Cancer Regulation by Stathmin1	2.75E-02		PPP1CC,GNAS,PPP2CA,GNAQ,ARHGEF18,PIK3CD,GNA13,PRKAR1A,PRKCB
Cell Cycle Control of Chromosomal Replication	2.95E-02		MCM6,POLE,DBF4,RPA2
IL-3 Signaling	3.02E-02	1.342	FOXO1,PIK3CD,ELK1,STAT1,PRKCB
Dendritic Cell Maturation	3.24E-02	1.414	NFKBID,PLCG2,IL15,IKBKE,PIK3CD,IGHG1,STAT1,CCR7
EIF2 Signaling	3.24E-02	↑ 2.236	EIF3G,PPP1CC,EIF4A3,RPL19,AGO2,PDPK1,PIK3CD,RPL18,FAU
Neuregulin Signaling	3.31E-02	1.342	PLCG2,PDPK1,ELK1,MATK,PRKCB
Leptin Signaling in Obesity	3.31E-02		FOXO1,PDE3B,PLCG2,PIK3CD,PRKAR1A
Interferon Signaling	3.39E-02		IRF9,STAT1,IFITM1
Huntington's Disease Signaling	3.63E-02	1.342	HSPA4,GLS,CLTB,APAF1,GNAQ,RCOR1,PDPK1,PIK3CD,GOSR1,PRKCB
LPS-stimulated MAPK Signaling	3.63E-02	↑ 2.236	NFKBID,IKBKE,PIK3CD,ELK1,PRKCB
NF-κB Activation by Viruses	3.63E-02	↑ 2.236	NFKBID,IKBKE,PIK3CD,CXCR5,PRKCB
Autoimmune Thyroid Disease Signaling	3.63E-02		GZMB,IGHG1,FASLG
IL-17A Signaling in Fibroblasts	3.63E-02		NFKBID,IKBKE,MMP1
AMPK Signaling	3.80E-02	0	GNAS,FOXO1,PPP2CA,EEF2,PDPK1,ILKAP,PIK3CD,PRKAR1A,ADRB2
PTEN Signaling	3.80E-02	-1.633	FOXO1,SYNJ1,PDPK1,IKBKE,PIK3CD,FASLG
PXR/RXR Activation	3.89E-02		ABCB1,FOXO1,NR3C1,PRKAR1A
CCR5 Signaling in Macrophages	3.89E-02		GNAS,PLCG2,FASLG,PRKCB
CREB Signaling in Neurons	4.07E-02	1.633	GNAS,PLCG2,GNAQ,PIK3CD,GNA13,ELK1,PRKAR1A,PRKCB
Formaldehyde Oxidation II (Glutathione-dependent)	4.17E-02		ESD
Glutamine Degradation I	4.17E-02		GLS
Notch Signaling	4.17E-02		CNTN1,APH1A,MAML2
Granzyme B Signaling	4.37E-02		GZMB,APAF1
fMLP Signaling in Neutrophils	4.37E-02	1.633	NFKBID,GNAS,NFATC3,PIK3CD,ELK1,PRKCB
CD28 Signaling in T Helper Cells	4.37E-02	1.342	NFKBID,NFATC3,PDPK1,IKBKE,PIK3CD,CTLA4
Gap Junction Signaling	4.79E-02		GNAS,PLCG2,CSNK1G3,GNAQ,PIK3CD,PRKAR1A,PRKCB,GJA4
Neuroinflammation Signaling Pathway	4.79E-02	0.905	CD200,NFATC3,PLCG2,GLS,APH1A,IKBKE,PIK3CD,STAT1,CSF1R,FASLG,BIRC2
Thrombopoietin Signaling	4.79E-02	1.000	PLCG2,PIK3CD,STAT1,PRKCB
Cell Cycle: G1/S Checkpoint Regulation	4.79E-02		FOXO1,GNL3,RBL1,SKP2
Phospholipase C Signaling	4.90E-02	1.890	GNAS,NFATC3,PLCG2,GNAQ,ARHGEF18,IGHG1,GNA13,PLD1,PRKCB

[a] Absence of a Z-score entry indicates that Z-score cannot be calculated by IPA.

Table S7. Ingenuity Predicted Upstream Regulators of DEGs in SG CD4+CD45RA- T cells of pSS cases versus nSS controls

Upstream Regulator	Regulator Type	p-value	Z-score[a]	Downstream Target Molecules Present in DEG Dataset
Interferon alpha	group	6.28E-05	↑2.172	CCR7,EPSTI1,FASLG,GLS,GZMB,IFITM1,IGFBP4,IKBKE,IL15,IL6ST,IRF9,OAS2,PARP9,PLAT,RABGAP1L,RBCK1,SPRY1,STAT1,TRIM5,UBA7,ZBP1
FAS	transmembrane receptor	1.57E-04	-1.695	AKAP8,BIRC2,CD200,CLASP1,CLTB,CSF1,DNAH17,FASLG,FOXO1,GDE1,MMP1,NR3C1,P2RY10,PIK3CD,PLD1,PPFIA1,PPP1CC,PRKCB,TFRC
IL10	cytokine	1.80E-04	1.884	CCR7,CD2,CSF1,CSF1R,CTLA4,CXCL13,FASLG,GZMB,IGFBP4,IL6ST,MMP1,NEDD9,NFKBID,NOD2,SELL,STAT1,TFRC
Creb	group	2.58E-04	0.199	CSF1R,DDX42,DYNC1LI2,EIF4EBP2,GNAS,MMP1,NDFIP2,NEDD9,NF1,NR3C1,OSBPL3,PDE3B,PLAT,POLE,PPP2CA,ZBP1
IFNA2	cytokine	2.73E-04	↑2.921	CXCL13,GEM,GNAS,GPATCH11,IFITM1,IRF9,OAS2,PARP9,PLAT,RBL1,STAT1,TRIM14,UBA7,ZBP1
TCR	complex	4.00E-04	0.965	CCR7,CD200,CTLA4,CXCL13,CXCR5,DKC1,FASLG,GZMB,KIR3DL1,NR3C1,PASK,PDE3B,PGAM1,PIK3CD,SELL,STAT1,TLE3,TXK
MID1	other	4.47E-04		PDPK1,PPP2CA
miR-6740-3p (miRNAs w/seed GUCUUCU)	mature microrna	5.07E-04	-1.265	ADI1,AIF1,C18orf25,CHD6,CRNN,EIF3G,FASLG,GCFC2,NDFIP2,RILPL2
miR-589-5p (and other miRNAs w/seed GAGAACC)	mature microrna	5.50E-04	↓-2.121	BABAM1,DHFR,FAM86B2/FAM86KP,PGAM1,PHKB,PNPO,SIRPG,SLC25A14
IFN Beta	group	6.27E-04	0.775	CCR7,IFITM1,IL15,IRF9,OAS2,SPRY1,STAT1,TRIM14,ZBP1
IFNL1	cytokine	6.29E-04	↑2.646	CCR7,GPATCH11,IFITM1,IRF9,OAS2,STAT1,TRIM14
CXCL13	cytokine	7.18E-04		CXCR5,MMP1,NFATC3
IL15	cytokine	8.06E-04	-0.487	CD2,CKS1B,COPB2,CSF1,FASLG,GALM,GNAS,GZMB,IFITM1,KIR3DL1,LYPLA1,PLCG2,PLD1,S1PR1,SELL,TCF7,TFRC,TRIM5,VBP1
IRF7	transcriptional	8.97E-04	1.709	CTLA4,IFITM1,IL15,IRF9,OAS2,PLAC8,STAT1,TRIM5,UBA7,ZBP1
mir-21	microrna	1.01E-03	-1.095	AIF1,FASLG,HMGA2,IGHG1,NIFK,NLRC5,OAS2,PGAM1,PLAT,PPP4R2,SPRY1,STAT1,UBA7
IL4	cytokine	1.16E-03	0.412	APH1A,CCR7,CD2,CD200,CSF1,CSF1R,CXCR5,DDHD1,DDX42,EIF3G,FASLG,IGFBP4,IGHG1,IL15,IL6ST,IRF9,MATK,MMP1,NEDD9,NR3C1,PLD1,SELL,ST6GAL1,STAT1,TCF7,TNFSF8,TXK,ZZZ3
ERBB2	kinase	1.27E-03	1.928	ADAM8,ATP6V1A,CHD6,CKS1B,CSF1R,DHFR,ELK1,EPSTI1,FEZ2,FOXO1,GLS,HMGA2,IGFBP4,IL6ST,MCM6,MMP1,MSMB,NEDD9,PDPK1,PIK3CD,PLAC8,PLAT,POLB,POLE,POLR3E,RPA2,RRM1,SKP2
FBXL5	enzyme	1.32E-03		SLC11A2,TFRC
RRP1B	other	1.45E-03		APAF1,DBF4,DHFR,FOXO1,MCM6,RBL1,RPA2,RPL19
miR-718 (miRNAs w/seed UUCCGCC)	mature microrna	1.51E-03	-0.816	DNMT3A,GOSR1,NLRC5,PDPK1,PLEKHG3,POLR3E
CD44	other	1.68E-03	0.926	AAK1,ABCB1,ADAM8,ADRB2,BIRC2,CCR7,COMMD3,FASLG,HDGF,NECAP2,SELL
CDKN2A	transcriptional	1.79E-03	0.503	APAF1,CCR7,CXCL13,CXCR5,DHFR,GNA13,GNAS,GNL3,IL15,MMP1,NR3C1,RRM1,SKP2,TPP1
KRAS	enzyme	1.86E-03	-1.026	AGO2,DHFR,DNMT3A,ELK1,FASLG,FKBP11,GNA13,GPC3,IFITM1,IRF9,MTRR,NAB1,NR3C1,PLAT,PLD1,PSMC6,SKP2,STAT1,TLE3,VMP1
miR-6513-3p (miRNAs w/seed CAAGUGU)	mature microrna	2.28E-03	-1.342	ADH7,BIRC2,EEF2,GGCT,SMARCA5
BCR (complex)	complex	2.35E-03	0.901	CCR7,CXCR5,FCMR,GZMB,IGHG1,S1PR1,SELL,TNFSF8
IL3	cytokine	2.39E-03	0.687	CSF1,CSF1R,CTLA4,EIF3G,FKBP3,GNA13,GNAS,HDGF,MMP1,NEDD9,NFATC3,NIFK,SELL,STT3A,TNFSF8
XBP1	transcriptional	2.57E-03	1.943	BET1,COG3,COPB2,EDEM1,ERP44,FKBP11,GGCX,MCFD2,S1PR1,SEC11A,VAMP2
Foxo	group	2.88E-03		ABCB1,FASLG,RICTOR
IFNA1/IFNA13	cytokine	3.06E-03	1.480	GEM,IFITM1,OAS2,STAT1,ZBP1
IL21	cytokine	3.13E-03	0.618	CCR7,CD2,CXCL13,CXCR5,FASLG,GZMB,IGHG1,OAS2,SELL
IL12 (complex)	complex	3.13E-03	-0.308	CCR7,CSF1,FASLG,GZMB,IL15,PLAC8,RPA2,SELL,TFRC,TXK
FOXP3	transcriptional	3.18E-03	-1.000	CTLA4,CXCL13,FASLG,PDE3B,SATB1,SELL,SKP2
IRF1	transcriptional	3.28E-03	0.490	ADAM8,EIF4A3,FASLG,IFITM1,IL15,IRF9,OAS2,SELL,STAT1
ZC3H14	other	3.54E-03		IL15,PLAT,RBL1
ERN1	kinase	3.63E-03	1.342	CNTN1,CREB3L2,EDEM1,FKBP11,IFITM1,OAS2,OSBP,SEC11A
miR-4637 (miRNAs w/seed ACUAACU)	mature microrna	3.67E-03	-1.342	CD200,GCFC2,MLF1,MRPL35,PLD1
let-7	microrna	4.12E-03	-1.265	CCR7,CKS1B,DBF4,FANCD2,HMGA2,MCM6,RRM1,SKP2,TAB2
MTOR	kinase	4.13E-03	-1.309	ATP2B1,CCR7,DHFR,ETFA,FDPS,PPP2CA,PRKAR1A,RAB4A,S1PR1,SELL,SKP2,ST6GAL1,STAT1
CD40LG	cytokine	4.19E-03	1.207	BIRC2,CCR7,CSF1,DAPP1,DDX42,FASLG,GZMB,IFITM1,IGHG1,IL15,RASSF2,SELL,STAT1,TFRC,TNFSF8,ZZZ3
OPRD1	g-protein coupled receptor	4.28E-03		ELK1,GZMB
PHLPP1	enzyme	4.29E-03		FOXO1,IL15,PRKCB
miR-6823-5p (miRNAs w/seed CAGGGUU)	mature microrna	4.44E-03	-1.414	APAF1,CASTOR2,COX7B,HDGF,MUC7,STAG2,TPP1,VAMP1
ELF4	transcriptional	4.44E-03	-1.000	ABCB1,CCR7,SELL,TAF7
CD3	complex	4.47E-03	-1.544	CD2,CKS1B,COPB2,CTLA4,EIF4A3,FASLG,FDPS,FKBP3,GNAS,GZMB,IFITM1,IRF9,LONP1,LYPLA1,MBD1,NDFIP2,PGAM1,PSMD12,STAT1,TCF7,TFRC,TRIM5,VBP1
TLR7	transmembrane receptor	4.67E-03	1.949	CCR7,CXCL13,FCMR,IFITM1,IRF9,OAS2,PLAT,STAT1
TP53	transcriptional	4.75E-03	0.461	ABCB1,ACOT11,ADAM8,ADRB2,APAF1,BIRC2,CCNL1,CKAP2,CKS1B,CSF1,CSF1R,DBF4,DHFR,ETFA,FASLG,FDPS,FKBP3,FOXO1,GNA13,GNL3,GTF3C2,IGFBP4,IPO7,IRF9,LYPLA1,MCM6,MMP1,NAB1,PDS5A,POLB,POLR3E,PPP1CC,PPP2CA,PPP4R2,PRKCB,PSMD12,PVT1,RASSF2,RBL1,RRM1,STAT1,TPP1,TRAP1,VMP1
miR-3140-3p (miRNAs w/seed GCUUUUG)	mature microrna	4.77E-03	↓-2.449	GCFC2,MCFD2,RBM18,SNX3,THRAP3,TSTD1
APP	other	4.93E-03	-1.745	AIF1,APH1A,ATP6V1A,CLTB,CSF1,CSF1R,DSCAML1,FASLG,FBXL20,IL6ST,MMP1,NDUFS6,NF1,PDPK1,PGAM1,PLAT,PPP2CA,PRKCB,RASSF2,RBL1,S1PR1,SATB1,SLC11A2,SSRP1,ST6GAL1,SUN5,TPP1,VAMP2

CD4	transmembrane receptor	4.97E-03	-1.941	CXCL13,CXCR5,FASLG,IL15
GAPDH	enzyme	4.97E-03	-1.964	GEM,IFITM1,OAS2,STAT1
miR-4533 (miRNAs w/seed GGAAGGA)	mature microrna	5.09E-03	↓-2.887	ARFIP1,CEP68,EIF4EBP2,HDGF,IGFBP4,MGA,OAS2,SELL,SIRPG,ST6GAL1,TPP1,VAMP2
TCF4	transcriptional	5.10E-03		ARHGEF18,CD2,CREB3L2,EDEM1,FASLG,FKBP11,MMP1,PLAC8,SELL,ZBP1
BNIP3L	other	5.16E-03	-1.387	CKAP2,FANCD2,PIK3CD,RBL1,TFRC
IL2	cytokine	5.22E-03	-0.233	ADAM8,CCR7,CD2,CSF1,CSF1R,CTLA4,CXCR5,DKC1,FASLG,FCMR,FOXO1,GNAQ,GNL3,GZMB,KIR3DL1,NIFK,PDE3B,PLAC8,SATB1,SELL
SET	phosphatase	5.55E-03	↓-2.000	BET1,FDPS,GZMB,ZCCHC17
EGR3	transcriptional	6.16E-03	-0.330	B3GALT6,ESD,FASLG,NF1
E2F1	transcriptional	6.29E-03	1.377	ABCB1,APAF1,BUB3,CBX7,DBF4,DHFR,DLEU1,FANCD2,FKBP3,FOXO1,MCM6,MMP1,RBL1,RCOR1,RPA2,RRM1,SMARCA5,TRAP1
TNFSF13B	cytokine	6.52E-03		CCR7,CXCR5,FOXO1,IL15,SELL
miR-155-5p (miRNAs w/seed UAAUGCU)	mature microrna	6.78E-03	↓-3.375	APAF1,ARFIP1,BET1,CSF1R,CTLA4,CUL4B,GNA13,HSDL1,IKBKE,RCOR1,SATB1,TAB2
FSH	complex	6.80E-03	0.721	ARHGAP35,ATP2B1,FILIP1L,FOXO1,GDE1,GEM,GNAS,GZMB,IGFBP4,PIK3CD,PLAT,PRKAR1A,RAB4A,RASSF2,STAT1,TFRC
miR-145-5p (and other miRNAs w/seed UCCAGUU)	mature microrna	6.84E-03	↓-2.155	AGFG1,BIRC2,COX16,EIF4EBP2,FKBP3,MMP1,PPP4R2,TARS2,TFRC
miR-3607-3p (miRNAs w/seed CUGUAAA)	mature microrna	7.03E-03	↓-2.236	CHN1,DBF4,DCTN6,IL6ST,P2RY10
TGFB1	growth factor	7.10E-03	1.688	ADI1,ARHGAP35,CCR7,CKS1B,CSF1,CSF1R,CTLA4,CXCL13,CXCR5,DKC1,DNMT3A,DYNLL1,EIF4A3,ELMO1,FASLG,FILIP1L,FOXO1,GALM,GEM,GNA13,GNAS,GZMB,HDGF,HMGA2,IGFBP4,IL15,LIMS1,MBD1,MMP1,MSMB,MTRR,NAB1,NEDD9,PIK3CD,PILRA,PLAT,PPP2CA,RBL1,RICTOR,RPA2,S1PR1,SLC25A14,SPRY1,SSRP1,STAT1,TAB2,TFRC,UBE3A
IRF3	transcriptional	7.12E-03	0.317	BIRC2,IL15,NLRC5,NR3C1,OAS2,PLAC8,PLCG2,STAT1,ZBP1
miR-6746-5p (miRNAs w/seed CGGGAGA)	mature microrna	7.12E-03	-0.333	ADAM8,ADI1,ARX,CCDC127,COX7B,GNAS,GNL3L,PHOSPHO1,PYCARD-AS1
STAT6	transcriptional	7.43E-03	-0.728	CCR7,CD2,DDHD1,IGHG1,IL15,IL6ST,IRF9,NEDD9,PLD1,ST6GAL1,TCF7,TXK
ARHGAP21	other	7.51E-03	1.000	IL15,PLAT,RBL1,TNFSF8
miR-634 (miRNAs w/seed ACCAGCA)	mature microrna	7.70E-03	-1.000	ADRB2,CKS1B,GNAQ,GNL3L,HSDL1,MBIP,PLD1,PNPO,TFRC
NFKB1	transcriptional	7.88E-03	1.302	ABCB1,AGO2,CSF1,CXCR5,FANCD2,FASLG,IGHG1,IKBKE,NOD2,POLB,STAT1
PLK4	kinase	8.20E-03		IL15,PLAT,RBL1
CD2	transmembrane receptor	8.26E-03	1.067	CCR7,FASLG,SELL,STAT1
miR-4272 (miRNAs w/seed AUUCAAC)	mature microrna	8.26E-03	-1.000	IL15,P2RY10,PSMC6,TFRC
RARB	and-dependent nuclear recep	8.31E-03		BIRC2,IFITM1,MMP1,SATB1,STAT1,UBA7
miR-329-3p (and other miRNAs w/seed ACACACC)	mature microrna	8.48E-03	-1.134	CCDC127,EPSTI1,FASLG,GZMB,HSPA4,PLAT,VOPP1
miR-6967-5p (and other miRNAs w/seed AGGGAGG)	mature microrna	8.56E-03	-0.894	COX7B,CREB3L2,EIF4EBP2,ELK1,ELMO1,FFAR3,GOSR1,IGFBP4,PGAM1,PHYHD1,PLD1,PNPO,RAB30,RASSF5,RHBDD2,SIRPG,SLC27A1,TMEM104,TPP1,VAMP2
DOCK8	other	8.71E-03	1.342	IL15,PLAT,RBL1,STAT1,TNFSF8
SENP7	peptidase	8.74E-03		BIRC2,DHFR
IL21R	transmembrane receptor	8.74E-03		CCR7,IGHG1
Rb	group	9.33E-03	-1.941	APAF1,ARX,DHFR,RBL1,RPA2
PLK2	kinase	9.42E-03		IL15,PLAT,RBL1
RC3H1	enzyme	9.42E-03		CCR7,CXCL13,IL6ST
HGF	growth factor	9.90E-03	↑2.440	APAF1,BIRC2,CKS1B,CSF1,DBF4,DKC1,DYNLL1,EDA,FASLG,GEM,GNA13,HDGF,IGFBP4,IL6ST,MMP1,NEK4,PGAM1,SKP2
ADORA2A	g-protein coupled receptor	1.02E-02	1.195	ATP2B1,CSF1R,EEF2,PGAM1,PPP2CA,PRKAR1A,SSRP1
TNFSF10	cytokine	1.04E-02	-0.436	BIRC2,FASLG,IFITM1,IRF9,STAT1,TFRC
miR-4268 (miRNAs w/seed GCUCCUC)	mature microrna	1.07E-02	-1.134	CLTB,IL6ST,MAML2,OSBP,RASSF5,SLC22A2,ZC3H8
BAK1	other	1.07E-02		APAF1,EDEM1,IL15
MAPK1	kinase	1.10E-02	↓-2.168	FASLG,FCMR,GLS,GRAMD1B,HIC2,IFITM1,IRF9,MMP1,OAS2,OSBPL3,PLD1,STAT1,TRIM14,TRIM5
TNFSF11	cytokine	1.11E-02	0.053	AIF1,CSF1R,DMPK,FOXO1,IKBKE,IL15,MMP1,NFATC3,PLD1,SLC11A2,STAT1
SASH1	other	1.14E-02	1.342	IL15,PLAT,RBL1,STAT1,TNFSF8
miR-3923 (miRNAs w/seed ACUAGUA)	mature microrna	1.14E-02	↓-2.236	AGPS,CKS1B,IL6ST,MLF1,UCKL1
TCIRG1	enzyme	1.15E-02		CTLA4,SELL
ZNF382	transcriptional	1.15E-02		HMGA2,IKBKE
miR-153-3p (miRNAs w/seed UGCAUAG)	mature microrna	1.21E-02	↓-2.121	FOXO1,LIMS1,NFATC3,SATB1,TAF7,TES,UCKL1,VAMP2
LCK	kinase	1.22E-02		CD2,ELK1,FASLG
BAX	transporter	1.27E-02		APAF1,BIRC2,EDEM1,IL15
miR-421-3p (and other miRNAs w/seed UCAACAG)	mature microrna	1.28E-02	↓-2.646	ADI1,CBX7,CD200,PPP1CC,RAB30,SPRY1,TFRC
miR-361-5p (miRNAs w/seed UAUCAGA)	mature microrna	1.29E-02	↓-2.236	CKS1B,DCTN6,RAB30,SPRTN,VBP1
RBL1	transcriptional	1.37E-02	-1.467	DHFR,MCM6,RBL1,RRM1,SKP2
HNF4A	transcriptional	1.37E-02	0	ADRB2,APH1A,BET1,CDKAL1,COPB2,DCAF13,EIF4EBP2,ELMO1,FBXW2,FDPS,FKBP3,FOXO1,GALM,GGCX,GNL3,IL15,IL6ST,LIMS1,MRPL33,MSMB,NAT10,NECAP1,NEK4,NOD2,NONO,OSBP,PARP9,PDE4DIP,PHKB,PNPO,POLB,POLR3E,RAB30,RASSF5,RBAK,RBL1,RBM18,RPA2,RPAIN,RPL18,RRM1,SEC11A,SKP2,SMARCA5,SNX3,STAT1,STT3A,TCF7,TFRC,THRAP3,TLE3,TPP2,UROS,VAMP1,VMP1,WTAP,ZNF410
TLR4	transmembrane receptor	1.45E-02	0.225	CCR7,CD200,DNMT3A,FASLG,IL15,MMP1,NFKBID,NOD2,PLAT,RBL1,STAT1,TFRC,TNFSF8
miR-4715-5p (miRNAs w/seed AGUUGGC)	mature microrna	1.45E-02	-1.890	FAM184B,FAU,INTS5,MLF1,MOCS1,PHKB,TFRC

FBXO42	other	1.46E-02		CSF1,STAT1
IFNL4	cytokine	1.46E-02		OAS2,STAT1
KCNA3	ion channel	1.46E-02		CTLA4,FOXO1
miR-19b-3p (and other miRNAs w/seed GUGCAAA)	mature microrna	1.48E-02	↓-3.317	AGFG1,ARFIP1,C5orf56,CACUL1,CCNL1,MRPL35,NDFIP2,S1PR1,SNX3,TXK,VMP1
miR-520d-5p (and other miRNAs w/seed UACAAAG)	mature microrna	1.49E-02	-1.000	DCTN6,HMGA2,RAB12,TFRC
miR-627-3p (miRNAs w/seed CUUUUCU)	mature microrna	1.51E-02	-1.890	DGKH,GLS,LOC107985899,PDS5A,PRKCB,RAB30,VBP1
miR-554 (miRNAs w/seed CUAGUCC)	mature microrna	1.60E-02	0	DCAF13,GZMB,HMGA2,MAN1C1
IFNG	cytokine	1.62E-02	↑2.052	ABCB1,AIF1,BIRC2,CD2,CD200,CSF1,CSF1R,ELK1,FASLG,FOXO1,GLS,GNA13,GNAS,GZMB,IFITM1,IGFBP4,IGHG1,IKBKE,IL15,IRF9,MMP1,NEDD9,NF1,NLRC5,NOD2,OAS2,PARP9,PLD1,RAB12,RBCK1,SEC11A,SELL,SLC11A2,STAT1,TFRC,TXK
CD28	transmembrane receptor	1.64E-02	-0.352	CTLA4,CXCL13,FASLG,FDPS,GZMB,IFITM1,MBD1,NDFIP2,PGAM1,PSMD12,TCF7,TFRC,VBP1
miR-4316 (miRNAs w/seed GUGAGGC)	mature microrna	1.70E-02	-1.633	DGKH,EPSTI1,EVC,FAM86B2/FAM86KP,SNX3,TPRN
BRAF	kinase	1.72E-02		BIRC2,CSF1,CSF1R,MMP1
miR-1252-5p (miRNAs w/seed GAAGGAA)	mature microrna	1.72E-02	↓-3.000	AAK1,DGKH,EIF4EBP2,IGFBP4,LPXN,SELL,SNX27,SPRY1,TFRC
miR-4288 (and other miRNAs w/seed UGUCUGC)	mature microrna	1.78E-02	↓-2.646	CCDC127,GCFC2,GGCT,METTL23,PLAC8,TFRC,TOMM6
miR-4778-5p (miRNAs w/seed AUUCUGU)	mature microrna	1.78E-02	-1.633	ABCB1,DBF4,DCTN6,GGCT,IL6ST,TAB2
miR-662 (miRNAs w/seed CCCACGU)	mature microrna	1.78E-02	↓-2.449	ARFIP1,CBX7,HSDL1,MCFD2,NLRC5,SIRPG
miR-320b (and other miRNAs w/seed AAAGCUG)	mature microrna	1.78E-02	↓-2.449	AGFG1,ARFIP1,ETFA,MBIP,MLF1,TFRC
Ifn	group	1.78E-02		IFITM1,IL15,OAS2,STAT1,TFRC,ZBP1
miR-93-3p (miRNAs w/seed CUGCUGA)	mature microrna	1.89E-02	↓-3.000	IL6ST,MCFD2,NFATC3,PLAC8,RASSF2,SNX27,TFRC,TOMM6,ZCCHC17
SATB1	transcriptional	1.94E-02	-1.450	DNMT3A,ENOSF1,EPSTI1,FASLG,FEZ2,PRKCB,S1PR1,SELL
BTK	kinase	1.95E-02	-1.633	CCR7,CD2,IFITM1,IRF9,OAS2,STAT1
TGM2	enzyme	1.96E-02	1.320	CXCR5,DLEU1,FFAR3,IRF9,MDN1,OAS2,PARP9,SELL,STAT1,UBA7
BCL6	transcriptional	2.01E-02	0.152	CCR7,CD2,CSF1,CTLA4,CXCR5,HNRNPLL,IRF9,UBA7,VWA5A
miR-609 (miRNAs w/seed GGGUGUU)	mature microrna	2.08E-02	0	GEM,GOSR1,HDGF,PLEKHF1,PNPO,PRKCB,SLC22A2,TMEM263
MGEA5	enzyme	2.08E-02	-0.832	CD302,CDKAL1,CREB3L2,CSF1R,ELK1,FDPS,HSDL1,IGFBP4,IL6ST,LIMS3/LIMS4,LPXN,PHKB,SLC27A1,STAT1
IRF9	transcriptional	2.08E-02		IFITM1,OAS2,STAT1
ARRDC3	other	2.12E-02		ADRB2
TFIIF	complex	2.12E-02		POLB
tubulin (complex)	complex	2.12E-02		GNAQ
ALG2	enzyme	2.12E-02		CREB3L2
RHOBTB2	enzyme	2.12E-02		BRMS1
FCRL4	other	2.12E-02		ELK1
FOXD2	transcriptional	2.12E-02		PRKAR1A
Integrin alpha V beta 3	complex	2.12E-02		MMP1
GRSF1	other	2.12E-02		AGO2
H2BFM	other	2.12E-02		DNMT3A
DNAJC24	other	2.12E-02		EEF2
HDLBP	transporter	2.12E-02		CSF1R
GGN	other	2.12E-02		ADRB2
RDH11	enzyme	2.12E-02		NR3C1
GGT7	enzyme	2.12E-02		ADRB2
FAF2	other	2.12E-02		NF1
TCFL5	transcriptional	2.12E-02		CD2
DNAJC15	other	2.12E-02		ABCB1
LCMT1	enzyme	2.12E-02		PPP2CA
RIC8A	other	2.12E-02		GNAQ
DPH2	enzyme	2.12E-02		EEF2
PCGF5	other	2.12E-02		MCC
ACBD3	other	2.12E-02		SLC11A2
TRPC5	ion channel	2.12E-02		ABCB1
MED25	other	2.12E-02		MMP1
SRSF9	enzyme	2.12E-02		NR3C1
SNAPC5	transcriptional	2.12E-02		ADRB2
SYP	transporter	2.12E-02		VAMP2
PCAT6	other	2.12E-02		KLHL12
CCNB1	kinase	2.12E-02		FASLG
mir-184	microrna	2.12E-02		AGO2
SOS2	other	2.12E-02		SELL
GBP1	enzyme	2.12E-02		MMP1

DPF2	other	2.12E-02		CXCL13
FDPS	enzyme	2.12E-02		ADRB2
PTGES2	transcriptional	2.12E-02		IRF9
ELP2	other	2.12E-02		HSPA4
NPIPA7 (includes others)	other	2.12E-02		ADRB2
PAGR1	other	2.12E-02		NR3C1
USP13	peptidase	2.12E-02		STAT1
SMARCD1	transcriptional	2.12E-02		MMP1
P4HA1	enzyme	2.12E-02		AGO2
PAQR3	other	2.12E-02		ELK1
PIM1	kinase	2.12E-02	1.000	ABCB1,CTLA4,GNL3,SKP2
miR-3074-5p (miRNAs w/seed UUCCUGC)	mature microrna	2.12E-02	0	LOC107985899,PREB,RILPL2,RP9
TBX21	transcriptional	2.12E-02		CXCR5,GZMB,IL6ST,SELL
BRCA1	transcriptional	2.15E-02	0.563	FOXO1,HMGA2,IFITM1,NEDD9,NR3C1,POLR3E,RBL1,STAT1
CDCA7L	other	2.17E-02		DHFR,RBL1
MDK	growth factor	2.17E-02		GZMB,PLAT
mir-137	microrna	2.17E-02		CKS1B,NECAP1
LTB	cytokine	2.17E-02		CXCL13,SELL
miR-1287-5p (miRNAs w/seed GCUGGAU)	mature microrna	2.23E-02	-1.912	AAK1,CHD6,CHN1,LIMS3/LIMS4,MBIP,MCC,TNRC6C
TBX2	transcriptional	2.25E-02	1.342	CCNL1,CKAP2,CKS1B,MCM6,RBL1
Focal adhesion kinase	group	2.29E-02		FASLG,SELL,SKP2
NFKBIA	transcriptional	2.34E-02	-1.664	BIRC2,CKS1B,CSF1,FASLG,GZMB,IL15,MATK,MMP1,NOD2,PLAT,POLB,RNF19A,RRM1,TFRC,UBE3A
miR-4699-5p (miRNAs w/seed GAAGAUU)	mature microrna	2.36E-02	↓-2.219	CDKAL1,LIMS3/LIMS4,MBIP,SPRTN,TFRC
IL18	cytokine	2.40E-02	-1.127	CCR7,CSF1,FASLG,GZMB,MMP1,SELL,TXK
TNFRSF8	transmembrane receptor	2.41E-02	↑2.000	CCR7,FASLG,GZMB,KIR3DL1
TFDP1	transcriptional	2.50E-02		APAF1,DHFR,RBL1
PDPK1	kinase	2.50E-02		CCR7,S1PR1,SELL
miR-3136-5p (and other miRNAs w/seed UGACUGA)	mature microrna	2.52E-02	-1.633	ABCB1,COX7B,FAU,GPATCH11,IL6ST,NEDD9
miR-4433b-3p (miRNAs w/seed AGGAGUG)	mature microrna	2.52E-02	↓-2.449	BABAM1,GCFC2,IGFBP4,SELENON,SHPRH,TFRC
ORMDL3	other	2.56E-02		ADAM8,OAS2
RGS1	other	2.56E-02		IL15,RBL1
NFATC2	transcriptional	2.58E-02	-0.218	CTLA4,FASLG,IL15,PLAT,PLD1,STAT1,TNFSF8
miR-143-3p (and other miRNAs w/seed GAGAUGA)	mature microrna	2.58E-02	↓-2.611	ATP6V1A,DNMT3A,GNL3,NECAP1,RHBDD2,SELL,TPP1
SAMSN1	other	2.60E-02	1.342	IL15,PLAT,RBL1,STAT1,TNFSF8
CREBBP	transcriptional	2.61E-02	-0.152	CCR7,CSF1,DHFR,EPN1,EPSTI1,FASLG,FDPS,GNAS,KLHL4,MGA,NR3C1,PDE3B,PLEKHF1,ST6GAL1
miR-140-3p (and other miRNAs w/seed ACCACAG)	mature microrna	2.62E-02	↓-2.449	AGPS,COX7B,FBXL20,STAG2,TFRC,UBAP2L
miR-5583-5p (and other miRNAs w/seed AACUAAU)	mature microrna	2.73E-02		BET1,RABGAP1L,VBP1
CNOT7	transcriptional	2.73E-02		IFITM1,OAS2,STAT1
CYP27B1	enzyme	2.73E-02		ATP2B1,CTLA4,IL15
IL10RA	transmembrane receptor	2.73E-02	-1.929	ABCB1,CCR7,CTLA4,DDHD1,FASLG,IKBKE,NLRC5,S1PR1,SELL,STAT1,ZBP1
26s Proteasome	complex	2.76E-02	-1.914	APAF1,BIRC2,FOXO1,MLF1,PPP2CA,S1PR1,STAT1
SP1	transcriptional	2.98E-02	0.670	ABCB1,DBF4,DHFR,DNMT3A,EDA,FASLG,GNAS,IGFBP4,IL15,MAT2B,MMP1,NF1,POLB,PRKCB,RBL1,SELL,SLC11A2,STAT1
YWHAQ	other	2.99E-02		APAF1,MMP1
LAT	other	2.99E-02		CSF1,CTLA4
TNFAIP2	other	2.99E-02		PLAT,RBL1
miR-4709-5p (miRNAs w/seed CAACAGU)	mature microrna	3.00E-02	↓-2.236	BET1,HSDL1,PPP1CC,SPRY1,TFRC
miR-5008-3p (and other miRNAs w/seed CUGUGCU)	mature microrna	3.03E-02	↓-2.121	CD2,CLASP1,GDE1,GJA4,TAB3,TFRC,TOMM5,VOPP1
ADAM10	peptidase	3.14E-02		APH1A,CSF1,GNA13,GNAQ,IL6ST
TNF	cytokine	3.16E-02	1.668	AAK1,ADAM8,ADRB2,APAF1,ATP2B1,BIRC2,CCR7,CLASP1,CSF1,CSF1R,CTLA4,CXCL13,CXCR5,DLEU1,ELK1,FASLG,FDPS,GEM,GLS,HSPA4,IFITM1,IGFBP4,IKBKE,IL15,KIAA1671,MAN1C1,MMP1,NEDD9,NOD2,NR3C1,OAS2,PIK3CD,PLAT,RRM1,SELL,SLC11A2,SLC27A1,ST6GAL1,STAT1,TFRC,TNFSF8,TPP2,VMP1,WTAP
miR-3551-5p (and other miRNAs w/seed CUUCACC)	mature microrna	3.18E-02	-0.816	AKAP8,FANCD2,GALM,GZMB,RBM8A,SLC22A2
ID3	transcriptional	3.21E-02	-0.651	APAF1,CCR7,CSF1,CXCR5,FOXO1,GNL3,SELL,TNFSF8
TSC1	other	3.21E-02		ATP6V1A,FOXO1,STAT1
CRP	other	3.21E-02		PRKCB,PSMC6,SELL
miR-4698 (miRNAs w/seed CAAAAUG)	mature microrna	3.24E-02	↓-2.000	AGFG1,DGKH,GGCT,PGAP1
NFKB2	transcriptional	3.24E-02		CCR7,CXCL13,CXCR5,SELL
E2f	group	3.30E-02	1.951	DBF4,DHFR,DLEU1,FANCD2,MCM6,RBL1
TLR9	transmembrane receptor	3.40E-02	1.947	CCR7,CSF1,FCMR,IFITM1,IRF9,OAS2,SPRY1,STAT1

HOXD10	transcriptional	3.42E-02	0	DAPP1,ERP44,SELENON,TNFSF8
E2F6	transcriptional	3.42E-02		APAF1,DHFR,PRKAR1A,RPA2
TRIM24	transcriptional	3.43E-02	↓-2.177	EPSTI1,IRF9,PLAC8,STAT1,UBA7
SP2	transcriptional	3.44E-02		DHFR,MAT2B
Hsp70	group	3.47E-02		GZMB,IL15,NOD2
CD40	transmembrane receptor	3.50E-02	0.068	CSF1,CTLA4,DAPP1,EDEM1,FASLG,IGHG1,IL15,SELL
IFNAR1	transmembrane receptor	3.55E-02	1.131	CSF1,IFITM1,IL15,OAS2,PLAT,STAT1
miR-1284 (and other miRNAs w/seed CUAUACA)	mature microrna	3.58E-02	-1.342	ADRB2,CARMIL1,TES,TFRC,VAMP1
mir-155	microrna	3.58E-02	-1.964	APAF1,IKBKE,RICTOR,S1PR1,TAB2
miR-3714 (miRNAs w/seed AAGGCAG)	mature microrna	3.61E-02	↓-2.000	ADI1,DLEU1,GCFC2,RAB43
miR-6838-3p (miRNAs w/seed AGUCCUG)	mature microrna	3.73E-02	-0.447	ATP6V1A,CRNN,HMGA2,MRPL35,NECAP1
ABL1	kinase	3.73E-02		CSF1,DHFR,MMP1
CD3E	transmembrane receptor	3.81E-02		CTLA4,FASLG,NFATC3,PIK3CD
miR-148a-3p (and other miRNAs w/seed CAGUGCA)	mature microrna	3.89E-02	↓-3.162	AGFG1,ARFIP1,C18orf25,FEZ2,S1PR1,SNX27,SNX3,TNRC6C,UBAP2L,VMP1
miR-1233-5p (and other miRNAs w/seed GUGGGAG)	mature microrna	3.90E-02	-0.775	BRMS1,CSF1R,FFAR3,HDGF,INTS5,MCFD2,MRPL35,PYCARD-AS1,RBCK1,RP9,SELENON,TCAF2,TMEM104,VAMP1,VAMP2
Cyclin E	group	3.91E-02		DHFR,RBL1
miR-6856-3p (miRNAs w/seed ACAGCCC)	mature microrna	3.94E-02	-0.378	FASLG,GNAQ,PHYHD1,RABGAP1L,TRMO,TXK,ZNF692
ITGB3	transmembrane receptor	4.01E-02	1.698	BIRC2,CSF1,LIMS1,MMP1
miR-1306-3p (miRNAs w/seed CGUUGGC)	mature microrna	4.01E-02	-1.000	MAT2B,STAT1,VBP1,ZNF285
miR-513c-5p (and other miRNAs w/seed UCUCAAG)	mature microrna	4.01E-02	↓-2.000	CKS1B,GGCT,TFRC,UBE3A
miR-3929 (and other miRNAs w/seed AGGCUGA)	mature microrna	4.01E-02		CKS1B,COX7B,DYNLL1
BACH2	transcriptional	4.01E-02		FOXO1,PDE3B,SATB1
miR-4474-3p (miRNAs w/seed UGUGGCU)	mature microrna	4.02E-02	↓-2.121	DCTN6,GOSR1,HDGF,MCFD2,NAB1,PRR14L,RAB30,RASSF2
miR-219b-5p (and other miRNAs w/seed GAUGUCC)	mature microrna	4.09E-02	-1.633	CCR7,CRNN,GNAS,PDE3B,UCKL1,USP3
Rab5	group	4.19E-02		ADRB2
ZFAT	transcriptional	4.19E-02		FOXO1
ADGRL2	g-protein coupled receptor	4.19E-02		ADRB2
FBXO44	enzyme	4.19E-02		ADRB2
2-Mar	enzyme	4.19E-02		TFRC
SENP5	peptidase	4.19E-02		BIRC2
GLRX3	enzyme	4.19E-02		TFRC
FZD1	g-protein coupled receptor	4.19E-02		ABCB1
LGALS8	other	4.19E-02		FASLG
RPL13A	other	4.19E-02		CXCL13
LAMA3	other	4.19E-02		MMP1
TPPP3	other	4.19E-02		ADRB2
NUBP1	other	4.19E-02		TFRC
RPL15	other	4.19E-02		HMGA2
GLUL	enzyme	4.19E-02		GLS
DR1	transcriptional	4.19E-02		DHFR
VAMP3	other	4.19E-02		VAMP2
ARHGEF28	other	4.19E-02		GNAQ
ZNF410	other	4.19E-02		MMP1
SOS1	other	4.19E-02		SELL
FBXO4	enzyme	4.19E-02		TERF1
RBMX	other	4.19E-02		SMN1/SMN2
GEMIN2	other	4.19E-02		SMN1/SMN2
KHDRBS2	other	4.19E-02		STAT1
MLF1	other	4.19E-02		SKP2
TFPI2	other	4.19E-02		ABCB1
DUSP2	phosphatase	4.19E-02		MMP1
APPL1	other	4.19E-02		VAMP2
DPH1	other	4.19E-02		EEF2
USP1	peptidase	4.19E-02		FANCD2
ANGPTL4	other	4.19E-02		STAT1
CRABP2	transporter	4.19E-02		APAF1
APH1B	peptidase	4.19E-02		APH1A
SNRPA	other	4.19E-02		SMN1/SMN2
RELB	transcriptional	4.22E-02	1.154	ABCB1,CXCL13,FANCD2,IGHG1

miR-2052 (miRNAs w/seed GUUUUGA)	mature microrna	4.22E-02	0	DGKH,HMGA2,IL15,IL6ST
miR-4301 (miRNAs w/seed CCCACUA)	mature microrna	4.22E-02	↓-2.000	BET1,CACUL1,KLHL12,MRPL35
MAP2K3	kinase	4.22E-02		FASLG,IRF9,MMP1,STAT1
miR-5009-3p (miRNAs w/seed CCUAAAU)	mature microrna	4.30E-02		NDFIP2,PPP4R2,VBP1
ZEB2	transcriptional	4.30E-02		CXCR5,PLCG2,SELL
miR-3605-5p (and other miRNAs w/seed GAGGAUG)	mature microrna	4.31E-02	-1.134	C18orf25,FFAR3,FKBP11,RAB30,SNX27,TIGIT,UCKL1
VHL	transcriptional	4.38E-02		AIF1,FILIP1L,NEDD9,PREB,SKP2,TFRC
miR-924 (miRNAs w/seed GAGUCUU)	mature microrna	4.39E-02	↓-2.236	CXCL13,ELK1,MRPL35,ZCCHC17,ZNF410
E2F4	transcriptional	4.40E-02		BUB3,CKS1B,DBF4,DHFR,FANCD2,MCM6,RBL1,RRM1,SMARCA5
CBX4	transcriptional	4.41E-02		DHFR,RBL1
DAXX	transcriptional	4.41E-02		FASLG,MMP1
miR-4703-3p (miRNAs w/seed GUAGUUG)	mature microrna	4.43E-02	0	ADH7,KLHL12,MCFD2,MUC7
miR-6835-3p (miRNAs w/seed AAAGCAC)	mature microrna	4.43E-02	↓-2.000	BIRC2,PARP9,TFRC,TMEM248
miR-4638-3p (and other miRNAs w/seed CUGGACA)	mature microrna	4.44E-02	-1.134	GALM,IL6ST,NDFIP2,SLC22A2,SLC5A10,TFRC,WTAP
EPO	cytokine	4.45E-02	1.524	DCTN6,FASLG,GCC2,NR3C1,PRKCB,RAB4A,SKP2,SLC11A2,SSRP1,TFRC
SPI1	transcriptional	4.47E-02	0.955	CCR7,CSF1,CSF1R,FOXO1,IRF9,MMP1,P2RY10,RPL18
miR-6786-3p (miRNAs w/seed GACGCCC)	mature microrna	4.57E-02	-0.447	ARX,BEST1,FANCD2,GNAS,PDPK1
miR-6814-5p (miRNAs w/seed CCCAAGG)	mature microrna	4.57E-02	↓-2.236	BABAM1,ELK1,FEZ2,IGFBP4,PARP9
IFN type 1	group	4.60E-02		CXCL13,STAT1,UBA7
IFNAR2	transmembrane receptor	4.60E-02		IFITM1,OAS2,UBA7
miR-8066 (miRNAs w/seed AAUGUGA)	mature microrna	4.65E-02	↓-2.000	BET1,IL6ST,TFRC,TRIM5
ZBTB16	transcriptional	4.68E-02	-1.000	CD2,FASLG,IL6ST,SATB1,SELL,TNFSF8
miR-4771 (miRNAs w/seed GCAGACU)	mature microrna	4.68E-02	-1.633	FILIP1L,GCFC2,KIR3DL1,LDLRAP1,ZCCHC17,ZZZ3
miR-6864-5p (miRNAs w/seed UGAAGGG)	mature microrna	4.70E-02	-1.890	CEP68,DHFR,ENOSF1,MEAF6,RILPL2,RPL18,VAMP2
IFNB1	cytokine	4.72E-02	1.871	IFITM1,IRF9,MCM6,NOD2,OAS2,SPRY1,STAT1,UBA7,ZBP1
WT1	transcriptional	4.72E-02	0.270	CSF1,CSF1R,CUL4B,ETFA,FDPS,RPL19,SPRY1,ST6GAL1,TRAP1
miR-760-3p (and other miRNAs w/seed GGCUCUG)	mature microrna	4.82E-02	-0.302	CLASP1,CSF1,GJA4,HDGF,HMGA2,MCC,PDPK1,PLD1,RAB43,SELENON,TCF7
IL7	cytokine	4.83E-02	0.271	CD2,CSF1,FOXO1,S1PR1,SELL,TNFSF8
TNFRSF1B	transmembrane receptor	4.88E-02		BIRC2,CXCL13,MMP1,SELL
ZBTB20	transcriptional	4.91E-02		GPC3,NEDD9,NR3C1
miR-6506-3p (miRNAs w/seed CGUAUCA)	mature microrna	4.91E-02		GNA13,TERF1,TMEM248
SST	other	4.91E-02		ABCB1,NR3C1,SSTR3
KLRC4-KLRK1/KLRK1	transmembrane receptor	4.93E-02		FASLG,TCF7
HELLS	enzyme	4.93E-02		PDS5A,RBL1
miR-616-3p (miRNAs w/seed GUCAUUG)	mature microrna	4.93E-02	-0.447	BRD1,FFAR3,TFRC,TOMM5,TXK
miR-4774-5p (miRNAs w/seed CUGGUAU)	mature microrna	4.93E-02	↓-2.236	ATP6V1A,DHFR,DYNLL1,TRIM5,USP3
miR-1184 (miRNAs w/seed CUGCAGC)	mature microrna	4.95E-02	-1.414	ADH7,FAM86B2/FAM86KP,KIR3DL1,NFKBID,PGAM1,RILPL2,STAT1,TFRC
PTGER4	g-protein coupled receptor	1.23E-01	↑2.000	CCR7,FBXL20,RASSF2,S1PR1,ST6GAL1
CREB1	transcriptional	3.18E-01	↑3.124	ARHGAP35,ESD,GEM,GLS,HSPA4,MCM6,MMP1,NF1,NFKBID,PDE3B,PLAT,SMN1/SMN2,TFRC
NUPR1	transcriptional	3.72E-01	↓-2.309	ACTR3B,ADRB2,CARMIL1,CASTOR2,FANCD2,GTF3C2,LOC100133315,PARP9,RILPL2,SKP2,SPRTN,TCAF2
NKX2-3	transcriptional	4.54E-01	↓-2.236	PARP9,PLD1,STAT1,TRIM5,UBA7

[a] Absence of a Z-score entry indicates that Z-score cannot be calculated by IPA.

Table S8. Gene Set Enrichment Analyses

Gene Set[a]	Reference	Size[d]	ES[e]	NES[f]	Nom p-value	FDR q-value
Up in $CXCR5^{+}CD57^{+}$ germinal center Tfh (vs. Th1, Th2, Tcm, Tem)	Chtanova, J Immunol 173 (1): 68-78	314	0.51	1.86	***0.000***	***0.003***
Up in $CXCR5^{+}CD57^{+}$ germinal center Tfh (vs. naïve, $CXCR5^{+}$ memory, Tem)	Kim, Blood 104 (7): 1952-60	55	0.56	1.64	***0.020***	***0.020***
Down in Th17 (vs. Th0)[b]	Tuomela, Oncotarget 7(12):13416-28	61	0.52	1.58	***0.012***	0.121
Up in Tcm (vs. Tem)	Chtanova, J Immunol 173 (1): 68-78	10	0.65	1.51	***0.053***	0.148
Up in activated Treg (vs. $CD45RA^{-}$ memory T)	Miyara, Immunity 30(6): 899-911	139	0.36	1.43	***0.034***	0.192
Up in $CD69^{+}$ Trm (vs. $CD69^{-}$ non-Trm)	Kumar, Cell Rep 20(12): 2921-34	12	0.53	1.25	0.235	0.351
Up in Th1 (vs. Th2)[c]	Rogge, Nat Genet 25(1): 96-101	112	0.29	1.17	0.244	0.437
Up in Tcm (vs. Tem and naive)	Novershtern, Cell 144(2): 296-309	13	0.41	1.10	0.351	0.500
Down in activated Treg (vs. $CD45RA^{-}$ memory T)	Miyara, Immunity 30(6): 899-911	110	0.21	1.05	0.330	0.541
Up in Tem (vs. Tcm and naive)	Novershtern, Cell 144(2): 296-309	16	-0.44	-1.36	0.111	0.640
Up in Th17 (vs. non-Th17)	Zhang, PLoS One 7(6): e38510	124	-0.20	-0.83	0.753	0.714
Up in Th1 (vs. Th2)	Chtanova, J Immunol 173 (1): 68-78	16	0.27	0.82	0.721	0.739
Down in $CD69^{+}$ Trm (vs. $CD69^{-}$ Trm)	Kumar, Cell Rep 20(12): 2921-34	13	0.30	0.84	0.667	0.759
Up in Th17 (vs. Th0)[b]	Tuomela, Oncotarget 7(12):13416-28	184	0.18	0.88	0.798	0.766
Up in Tem (vs. Tcm)	Chtanova, J Immunol 173 (1): 68-78	14	-0.33	-0.94	0.533	0.771
Up in Th2 (vs. Th1)[c]	Rogge, Nat Genet 25(1): 96-101	37	-0.24	-0.86	0.677	0.783
Up in Th2 (vs. Th1)	Chtanova, J Immunol 173 (1): 68-78	20	0.32	0.89	0.589	0.823

[a]Tfh= T follicular helper, Tcm = T central memroy, Tem = T effector memroy, Trm = T resident memory;

[b]Inclusion required reads per kilobase of transcript (RPKM)>10, RPKM>mean, logFC≥1.0;

[c]Inclusion required FC ≥2.0;

[d]Number of genes in gene set;

[e]Enrichment Score;

[f]Normalized Enrichment Score
